# The global epidemiology and health burden of the autism spectrum: findings from the Global Burden of Disease Study 2021

**DOI:** 10.1016/S2215-0366(24)00363-8

**Published:** 2025-02

**Authors:** Global Burden of Disease Study 2021 Autism Spectrum Collaborators, Damian F Santomauro, Holly E Erskine, Ana M Mantilla Herrera, Paul Anthony Miller, Jamileh Shadid, Hailey Hagins, Isaac Yeboah Addo, Qorinah Estiningtyas Sakilah Adnani, Bright Opoku Ahinkorah, Ayman Ahmed, Fadwa Naji Alhalaiqa, Mohammed Usman Ali, Sabah Al-Marwani, Joseph Uy Almazan, Sami Almustanyir, Farrukh Jawad Alvi, Yasser Sami Abdel Dayem Amer, Edward Kwabena Ameyaw, Sohrab Amiri, Catalina Liliana Andrei, Dhanalakshmi Angappan, Catherine M Antony, Aleksandr Y Aravkin, Tahira Ashraf, Jose L Ayuso-Mateos, Amadou Barrow, Kavita Batra, Maryam Bemanalizadeh, Akshaya Srikanth Bhagavathula, Sonu Bhaskar, Jasvinder Singh Singh Bhatti, Srinivasa Rao Bolla, Gabrielle Britton, Joao Mauricio Castaldelli-Maia, Ferrán Catalá-López, Arthur Caye, Vijay Kumar Chattu, Yuen Yu Chong, Liliana G Ciobanu, Samuele Cortese, Natalia Cruz-Martins, Berihun Assefa Dachew, Xiaochen Dai, Amira Hamed Darwish, Mohsen Dashti, Alejandro de la Torre-Luque, Daniel Diaz, Delaney D Ding, Angel Belle Cheng Dy, Arkadiusz Marian Dziedzic, Sepideh Ebrahimi Meimand, Omar Abdelsadek Abdou El Meligy, Iman El Sayed, Frank J Elgar, Adeniyi Francis Fagbamigbe, Pawan Sirwan Faris, Andre Faro, Nuno Ferreira, Irina Filip, Florian Fischer, Aravind P Gandhi, Balasankar Ganesan, Miglas Welay Gebregergis, Mesfin Gebrehiwot, Bardiya Ghaderi Yazdi, Mohammad-Reza Ghasemi, Afsaneh Ghasemzadeh, Sasidhar Gunturu, Veer Bala Gupta, Vivek Kumar Gupta, Sobia Ahsan Halim, Brian J Hall, Fulei Han, Josep Maria Haro, Ahmed I Hasaballah, Simon I Hay, Darren Hedley, Bartosz Helfer, Md Mahbub Hossain, Bing-Fang Hwang, Umar Idris Ibrahim, Mehran Ilaghi, Md Rabiul Islam, Sheikh Mohammed Shariful Islam, Mahalaxmi Iyer, Khushleen Jaggi, Haitham Jahrami, Elham Jamshidi, Ali Khaleghi, Abdul Aziz Khan, Mohammad Jobair Khan, Feriha Fatima Khidri, Kwanghyun Kim, Hyun Yong Koh, Manasi Kumar, Iván Landires, Long Khanh Dao Le, Seung Won Lee, Zhihui Li, Stephen S Lim, Jose Martinez-Raga, Roy Rillera Marzo, Indu Liz Matthew, Andrea Maugeri, Tomislav Mestrovic, Philip B Mitchell, Salahuddin Mohammed, Ali H Mokdad, Lorenzo Monasta, Fateme Montazeri, Matías Mrejen, Faraz Mughal, Christopher J L Murray, Woojae Myung, Javaid Nauman, Charles Richard James Newton, Huong Lan Thi Nguyen, Chisom Adaobi Nri-Ezedi, Vincent Ebuka Nwatah, Adeolu Olufunso Oladunjoye, Isaac Iyinoluwa Olufadewa, Michal Ordak, Nikita Otstavnov, Raul Felipe Palma-Alvarez, Romil R Parikh, Seoyeon Park, Maja Pasovic, Jay Patel, Marcos Pereira, Maria Odete Pereira, Michael R Phillips, Guilherme V Polanczyk, Mohammad Pourfridoni, Jagadeesh Puvvula, Amir Radfar, Fakher Rahim, Mosiur Rahman, Muhammad Aziz Rahman, Amir Masoud Rahmani, Masoud Rahmati, Zubair Ahmed Ratan, Taeho Gregory Rhee, Luca Ronfani, Priyanka Roy, Basema Ahmad Saddik, Amene Saghazadeh, Joseph W Sakshaug, Sana Salehi, Vijaya Paul Samuel, Senthilkumar Sankararaman, Aswini Saravanan, Maheswar Satpathy, Austin E Schumacher, David C Schwebel, Mario Šekerija, Arman Shafiee, Saeed Shahabi, Muhammad Aaqib Shamim, João Pedro Silva, Yonatan Solomon, Lourdes Bernadette C Sumpaico-Tanchanco, Chandan Kumar Swain, Rafael Tabarés-Seisdedos, Mohamad-Hani Temsah, Samuel Joseph Tromans, Lilian Tzivian, Ravi Prasad Varma, Andres Fernando Vinueza Veloz, Maria Fernanda Vinueza Veloz, Mandaras Tariku Walde, Muhammad Waqas, Nuwan Darshana Wickramasinghe, Renjulal Yesodharan, Dong Keon Yon, Yoosik Youm, Burhan Abdullah Zaman, Youjie Zeng, Magdalena Zielińska, Harvey A Whiteford, Traolach Brugha, James G Scott, Theo Vos, Alize J Ferrari

## Abstract

**Background:**

High-quality estimates of the epidemiology of the autism spectrum and the health needs of autistic people are necessary for service planners and resource allocators. Here we present the global prevalence and health burden of autism spectrum disorder from the Global Burden of Diseases, Injuries, and Risk Factors Study (GBD) 2021 following improvements to the epidemiological data and burden estimation methods.

**Methods:**

For GBD 2021, a systematic literature review involving searches in PubMed, Embase, PsycINFO, the Global Health Data Exchange, and consultation with experts identified data on the epidemiology of autism spectrum disorder. Eligible data were used to estimate prevalence via a Bayesian meta-regression tool (DisMod-MR 2.1). Modelled prevalence and disability weights were used to estimate health burden in years lived with disability (YLDs) as the measure of non-fatal health burden and disability-adjusted life-years (DALYs) as the measure of overall health burden. Data by ethnicity were not available. People with lived experience of autism were involved in the design, preparation, interpretation, and writing of this Article.

**Findings:**

An estimated 61·8 million (95% uncertainty interval 52·1–72·7) individuals (one in every 127 people) were on the autism spectrum globally in 2021. The global age-standardised prevalence was 788·3 (663·8–927·2) per 100 000 people, equivalent to 1064·7 (898·5–1245·7) autistic males per 100 000 males and 508·1 (424·6–604·3) autistic females per 100 000 females. Autism spectrum disorder accounted for 11·5 million (7·8–16·3) DALYs, equivalent to 147·6 (100·2–208·2) DALYs per 100 000 people (age-standardised) globally. At the super-region level, age-standardised DALY rates ranged from 126·5 (86·0–178·0) per 100 000 people in southeast Asia, east Asia, and Oceania to 204·1 (140·7–284·7) per 100 000 people in the high-income super-region. DALYs were evident across the lifespan, emerging for children younger than age 5 years (169·2 [115·0–237·4] DALYs per 100 000 people) and decreasing with age (163·4 [110·6–229·8] DALYs per 100 000 people younger than 20 years and 137·7 [93·9–194·5] DALYs per 100 000 people aged 20 years and older). Autism spectrum disorder was ranked within the top-ten causes of non-fatal health burden for people younger than 20 years.

**Interpretation:**

The high prevalence and high rank for non-fatal health burden of autism spectrum disorder in people younger than 20 years underscore the importance of early detection and support to autistic young people and their caregivers globally. Work to improve the precision and global representation of our findings is required, starting with better global coverage of epidemiological data so that geographical variations can be better ascertained. The work presented here can guide future research efforts, and importantly, decisions concerning allocation of health services that better address the needs of all autistic individuals.

**Funding:**

Queensland Health and the Bill & Melinda Gates Foundation.

## Introduction

Autism spectrum disorder is a developmental condition characterised by persistent difficulties in social communication and interaction, challenges related to sensory processing, repetitive behaviours, interests, or activities, and in some instances intellectual disability, all of which occur at varying levels of severity.^1^ Autistic people are at an increased risk of social isolation, academic or employment difficulties, and might require psychosocial support into adulthood.^2^ Early diagnosis and intervention can improve outcomes for autistic people, but many do not receive early support.^3^, ^4^, ^5^, ^6^ Accurate epidemiological estimates are essential for strategic service planning and resource allocation for autistic people and are generated as part of the Global Burden of Diseases, Injuries, and Risk Factors Study (GBD).^7^

In this Article, we use identity-first language, as preferred by most people with lived experience of the autism spectrum.^8^, ^9^ This approach puts any reference to the autism spectrum first within a statement. However, we do acknowledge that some people with a diagnosis of autism spectrum disorder prefer person-first language, which emphasises the person first within a statement**.**

**Research in context**

**Evidence before this study**

Before this study, the most recent comprehensive review of the epidemiological modelling, prevalence, and health burden of the autism spectrum led by the Global Burden of Disease Collaborative Network was published on Aug 1, 2014 and reported on estimates from the 2010 iteration of the Global Burden of Diseases, Injuries, and Risk Factors Study (GBD). Each subsequent iteration of the GBD produced updated estimates of autism spectrum disorder disability, each with a series of improvements to the methods used, as well as the datasets underpinning disability estimates. We searched PubMed on June 5, 2024 with the following search terms: (((pervasive[Title/Abstract] AND disorder*[Title/Abstract]) OR (asperger*[Title/Abstract])) OR (autis*[Title/Abstract])) AND (global[Title/Abstract] AND 2021[Title/Abstract] AND (“GBD 2021”[Title/Abstract] OR disability[Title/Abstract] OR prevalence[Title/Abstract] OR burden[Title/Abstract])). This search yielded 14 studies; however, no studies covered GBD 2021 findings for the autism spectrum by location, age, sex, and year.

**Added value of this study**

This study presents updated global estimates of the prevalence and health burden of autism spectrum disorder for GBD 2021 following substantial revisions to their estimation process from previous iterations of the GBD. We present key methodological improvements in GBD 2021, such as exclusion of studies relying on passive case finding, new data from a systematic review update, and revisions to the estimation of disability weights. These updates contributed to substantial changes in the estimated prevalence and health burden of autism spectrum disorder. Globally, we estimated that one in 127 individuals in 2021 were autistic with prevalence and health burden persisting across the lifespan. Autism spectrum disorder was most common among children and adolescents younger than 20 years, for whom it ranked within the top ten causes of non-fatal health burden. Our analysis expands on the GBD 2021 capstone publication that presented the emerging trends in burden across all 371 diseases and injuries, but did not cover in detail the epidemiology and health burden of the autism spectrum.

**Implications of all the available evidence**

The revised estimation process and resulting prevalence and health burden estimates of autism spectrum disorder from GBD 2021 have important implications for future research, health-care provision, and policy planning. Our estimates highlight the necessity for early detection and lifelong support services for individuals on the autism spectrum. The persistence of the health burden across the lifespan demonstrates the need for policy planning and health-care provision that caters to autistic individuals at all stages of life. The limitations of our study, including the scarcity of epidemiological data, point to the need for more diagnostic surveys in many parts of the world. Our study highlights the pressing need for more comprehensive research and policy initiatives that can better meet the diverse needs of the global autistic population and improve their overall quality of life.

GBD is the largest scientific effort estimating prevalence and health burden of disorders, diseases, and injuries.^7^ Seven iterations of the GBD reporting on the autism spectrum have been completed, each quantifying the health burden using the disability-adjusted life-year (DALY). Each DALY represents a year of healthy life lost due to a cause. The term health burden in this Article refers specifically to DALY as it is used within GBD.

The global age-standardised prevalence of the autism spectrum reported by GBD 2019 was 369·4 per 100 000 people,^10^ which was relatively low compared with the prevalence of autism spectrum disorder from active case-finding methods (in which prevalence is estimated using a diagnostic instrument in a general population survey supplemented by a search in case registries or special education facilities).^11^, ^12^, ^13^ This low prevalence was largely attributed to the incorporation of estimates relying on passive case finding (in which prevalence is estimated using the number of pre-existing diagnoses identified through databases such as administrative or educational records). This type of prevalence estimation relies on autistic people being correctly identified through existing health-care practices within the population, which have historically underestimated prevalence.^14^ Bias corrections for these studies were implemented, but a method to incorporate how this bias varies by geography and time was not possible given the scarce available data reporting the proportion of autistic people represented using passive case-finding methods. For GBD 2021, estimates that made use of passive case finding were removed from the epidemiological modelling. This change, together with new data and revisions to the estimation of disability weights, has substantially changed estimates of the prevalence and health burden of autism spectrum disorder. These findings therefore supersede those from GBD 2019 and all previous GBD iterations. This Article summarises the revised process for estimating the prevalence and DALYs of autism spectrum disorder in 2021 and presents an overview of the prevalence and DALYs by age, sex, and geography. This Article was produced as part of the GBD Collaborator Network and in accordance with the GBD protocol.^15^

## Methods

This study adhered to the Guidelines for Accurate and Transparent Health Estimates Reporting (appendix 2 pp 12–13).^16^ A graphical overview of the process to estimate the prevalence and DALYs of autism spectrum disorder is presented in appendix 2 (p 5). GBD 2021 followed a cause hierarchy with four levels, with autism spectrum disorder placed at level 3 (appendix 2 p 2).^7^ Prevalence and DALYs in GBD 2021 were estimated by sex (male and female), 25 age groups, year (1990 to 2021), and 204 countries and territories that were grouped into 21 regions and seven super-regions. For all estimates, the 95% uncertainty intervals (UIs) were calculated as the 2·5th and 97·5th percentiles of the 500 draws from the posterior distribution of each step in the estimation process. Age-standardised rates were estimated using the GBD world population age standard.^17^ People with lived experience of autism were involved in the design, preparation, interpretation, and writing of this Article. Ethical approval and participant consent were not sought. No primary data collection was undertaken, and our dataset included non-identifiable and pre-aggregated data from existing published and grey literature sources.

### Search strategy

A systematic review was first done on Aug 11, 2017 to capture all available data sources on the epidemiology of autism spectrum disorder for GBD 2017, when autism spectrum disorder was first modelled as a unifying diagnosis for individuals previously meeting criteria for DSM-IV autism and Asperger's syndrome and other autism spectrum disorders, to be consistent with DSM-5.^1^ No start date was specified in the initial search. An update to this systematic review was done on April 12, 2021 as part of the GBD 2021 Study. Electronic databases PubMed, Embase, and PsycINFO were searched using a search string developed with a research librarian (appendix 2 p 14). Reference lists of sourced reviews were searched for relevant studies. The Global Health Data Exchange was searched and GBD collaborators were consulted for additional sources. Both reviews adhered to Preferred Reporting Items for Systematic Reviews and Meta-Analyses^18^, ^19^ (PRISMA) statement guidelines (appendix 2 pp 6–7, 15–17). Titles and abstracts were screened then full-text studies passing the initial screening were assessed. Two reviewers checked studies for eligibility and disagreements were resolved by DS, HE, and AF.

### Inclusion criteria and data extraction

To be included, data sources needed to report the prevalence, incidence, or excess mortality of autism spectrum disorder in a sample representative of the general population. We followed DSM-5 and ICD-11 criteria for autism spectrum disorder. Estimates from older criteria (eg, DSM-IV) were accepted, and their utility were assessed. Studies using criteria established before the DSM-III were excluded. No language restrictions were applied. Studies had to report enough data to calculate uncertainty surrounding estimates (eg, CI or sample size). For GBD 2021, prevalence estimates relying on passive case finding (eg, from administrative records) known to underestimate prevalence were excluded. The extent of underestimation in prevalence from these studies varies between locations and over time and was therefore difficult to adjust accordingly. Because of an overall scarcity of data on excess mortality, we were unable to apply the same exclusion criteria to the excess mortality data included within our analysis, which continued to rely on passive case finding.

Data extracted included location, age or ages, sex, number of autistic people, sample size, uncertainty, parameter type, year or years of data collection, diagnostic instruments, criteria, and sampling methods.

### Estimation of epidemiology

Estimates of prevalence, incidence, and excess mortality were input into the Bayesian meta-regression tool, DisMod-MR 2.1.^20^ DisMod-MR 2.1 pools heterogeneous epidemiological estimates and generates internally consistent estimates of prevalence, incidence, and excess mortality. DisMod-MR 2.1 generates estimates for locations that are missing raw data by drawing on estimates from surrounding locations. The tool achieves this result by generating estimates across five levels, comprising global, super-region, region, country, and subnational locations, with prevalence from the higher level acting as a prior for the lower geographical level. More detail on DisMod-MR 2.1 is described elsewhere.^7^, ^20^

Two adjustments were applied to estimates before analysis in DisMod-MR 2.1. First, data aggregated across males and females within the study sample were split into sex-specific estimates using available data on the sex ratio of autism spectrum disorder (appendix 2 p 2). Second, known sources of bias in the input prevalence data were adjusted using network meta-regressions by Meta-Regression—Bayesian, Regularised, Trimmed (MR-BRT). More detail on MR-BRT is available elsewhere.^21^ Prevalence data were considered reference (optimal) if the study included a general population survey with additional case finding (eg, household survey supplemented with an investigation in special education services) or population screening*.* General population surveys without additional case finding were adjusted with a bias correction (appendix 2 p 2). Studies reporting the prevalence of DSM-IV autistic disorder and ICD-10 childhood autism without reporting the prevalence of autism spectrum disorder were included with an upwards adjustment to reflect estimates of autism spectrum disorder (appendix 2 p 2).

Several prior settings informed the DisMod-MR 2.1 analysis on the basis of the available data and feedback from experts (appendix 2 p 2). We allowed a time window of 15 years because of the scarcity of data across time, which meant estimates informed prevalence 15 years before and after they were collected. DisMod-MR 2.1 produced age-sex-specific prevalence and excess mortality estimates across 204 countries and territories between 1990 and 2021.

### Estimation of severity distribution

In GBD 2021, health burden was estimated across varying levels of severity by cause. The term sequalae referred to mutually exclusive health states differing by levels of severity. A cause could have one or several sequalae, each with its own corresponding disability weight. Autism spectrum disorder contributed to the intellectual disability envelope and therefore the sequelae for autism spectrum disorder comprised six levels of intellectual disability: none (intelligence quotient [IQ] >84); borderline (IQ 70–84); mild (IQ 50–69); moderate (IQ 35–49); severe (IQ 20–34); and profound (IQ <20). Intellectual disability is considered to be an impairment, for which the prevalence is modelled and treated as an envelope. The prevalences of all conditions that contribute to the envelope are adjusted not to exceed the total prevalence of the impairment.^7^

19 studies using the aforementioned reference-case definition and reporting the proportion of autistic people with intellectual disability were sourced from the systematic reviews. A series of meta-analyses were done in R using the metafor package^22^ to estimate the proportion of autistic people by each level of intellectual disability. The hierarchy of meta-analyses that we did is shown in appendix 2 (pp 3–4, 8). This process produced severity proportions to apportion prevalence estimates into sequela-specific prevalence by age, sex, year, and location (table 1).

**Table 1:** Severity proportions and disability weights for autism spectrum sequelae

	**Severity proportion (95% UI)**	**Disability weight (95% UI)**
Autism spectrum disorder without intellectual disability	0·446 (0·395–0·496)	0·169 (0·114–0·236)
Autism spectrum disorder with borderline intellectual disability	0·197 (0·159–0·235)	0·178 (0·123–0·244)
Autism spectrum disorder with mild intellectual disability	0·149 (0·110–0·191)	0·205 (0·149–0·273)
Autism spectrum disorder with moderate intellectual disability	0·139 (0·101–0·182)	0·252 (0·192–0·318)
Autism spectrum disorder with severe intellectual disability	0·056 (0·034–0·084)	0·302 (0·236–0·373)
Autism spectrum disorder with profound intellectual disability	0·014 (0·006–0·026)	0·336 (0·261–0·418)

UI=uncertainty interval.

### Estimation of disability weights

Disability weights represent health loss due to a cause on a scale of zero (no health loss) to one (death). Disability weights were derived from surveys of the general population done in Bangladesh, Hungary, Indonesia, Italy, Peru, Sweden, Tanzania, the Netherlands, and the USA, and an open-access internet survey available in English, Spanish, and Mandarin.^23^, ^24^ Surveys contained lay descriptions of GBD sequalae that used non-clinical language to describe each state in 35 words or fewer. Participants were asked to identify the healthier option within pairs of lay descriptions. Their responses were anchored between zero and one using questions on population health equivalence comparing the benefits of lifesaving and disease-prevention programmes across sequalae. The disability weights generated for autism spectrum disorder were across the six levels of intellectual disability (table 1) and were calculated as a multiplicative function of the autism spectrum disorder disability weights, intellectual disability weights, and the proportion of autistic people estimated with each level of intellectual disability (appendix 2 pp 4, 18). Disability weights and severity proportions were consistently applied across age, sex, and location.

### Estimation of DALYs

Health burden was quantified using DALYs, which is the aggregate of two health metrics: years lived with disability (YLD), capturing the disability (non-fatal burden) of a cause; and years of life lost (YLL), capturing the fatal burden of a cause.^7^ There were no deaths attributable to autism spectrum disorder as the underlying cause in GBD 2021, and therefore DALYs were composed entirely of YLDs.

Sequela-specific prevalence estimates were multiplied by their respective disability weights to calculate YLDs. Since an individual can have several sequelae simultaneously (ie, more than one cause at a given time), we needed to adjust YLDs for comorbidity. The co-occurrence of sequelae was simulated within a population of 20 000 simulated individuals by age, sex, location, and year, and YLDs were adjusted accordingly to ensure cumulative disability weights for any simulant did not exceed one (appendix 2 p 4).

### Role of the funding source

The funder of the study had no role in study design, data collection, data analysis, data interpretation, or writing of the report.

## Results

**Figure 1:**
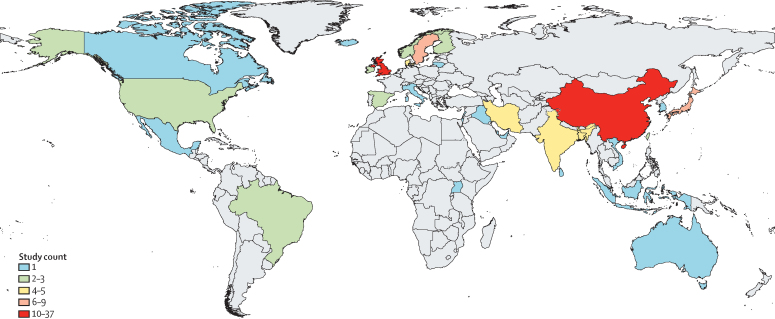
Number of studies per country used to inform the estimation of the prevalence of autism spectrum disorder Locations coloured grey did not have eligible epidemiological data on autism spectrum disorder. Dotted lines indicate disputed territories.

DisMod-MR 2.1 was informed by 105 studies reporting 228 prevalence estimates across 33 countries and 11 of 21 regions (PRISMA diagrams in appendix 2 pp 6–7; figure 1). Most prevalence studies focused on childhood and adolescence with only six studies reporting prevalence for adults aged 20 years or older. There were six studies reporting 24 estimates of excess mortality across six countries within two regions. Eligible studies are available via the GBD 2021 Data Input Sources Tool.^25^

**Figure 2:**
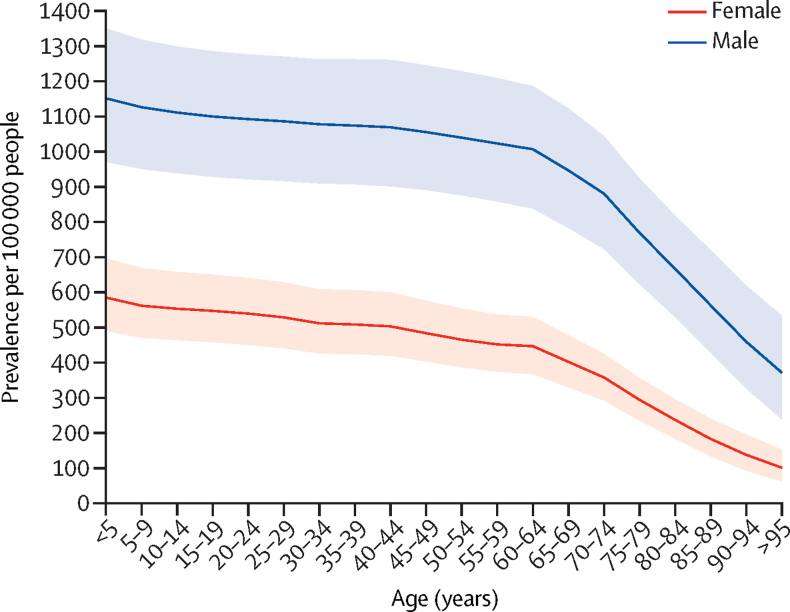
Prevalence of autism spectrum disorder per 100 000 people, by age and sex, 2021 The thick line represents the prevalence of autism spectrum disorder by age and the ribbon around this line represents its 95% UI. UI=uncertainty interval.

The global age-standardised prevalence of autism spectrum disorder in 2021 was 788·3 (95% UI 663·8–927·2) per 100 000 people. This finding equates to 61·8 (52·1–72·7) million autistic people globally. The age-standardised prevalence in 1990 was 773·2 (651·3–914·7) per 100 000 people. In 2021 the age-standardised prevalence for males was significantly higher than for females, with 1064·7 (898·5–1245·7) autistic males per 100 000 males compared with 508·1 (424·6–604·3) autistic females per 100 000 females. Prevalence was highest at birth and decreased with age (figure 2). Between birth and age 59 years, prevalence ranged between 169·2 (115·0–237·4) per 100 000 people in children younger than 5 years to 132·0 (90·9–183·6) in adults aged 55–59 years. The rate of decline in prevalence increased from aged 60 years with prevalence ranging between 127·6 (87·8–178·2) per 100 000 people in adults aged 60–64 years and 27·9 (17·1–42·8) in adults aged 95 years and older. The estimated excess mortality among autistic people was 564·8 (474·4–666·8) per 100 000 autistic males per year and 740·1 (633·4–854·9) per 100 000 autistic females per year globally (age-standardised) in 2021.

The highest estimated prevalence was in the high-income super-region at 1090·2 (95% UI 916·3–1279·3) autistic people per 100 000 people. Within this super-region, the highest prevalence was estimated in high-income Asia Pacific, with 1559·5 (1311·3–1832·4) autistic people per 100 000 people, where the highest prevalence was estimated for Japan (1586·9 [1333·2–1864·1] autistic people per 100 000 people). The super-region with the lowest prevalence was southeast Asia, east Asia, and Oceania at 669·2 (560·7–791·5) autistic people per 100 000 people. Within this super-region, the lowest prevalence was estimated in east Asia, with 660·7 (549·4–785·9) autistic people per 100 000 people, where the lowest prevalence was estimated in China (655·7 [545·1–780·4] autistic people per 100 000 people). The region with the lowest prevalence was tropical Latin America (614·5 [514·7–732·3] autistic people per 100 000 people), and the lowest prevalence globally was estimated for Bangladesh (588·2 [486·7–696·6] autistic people per 100 000 people) within south Asia. Prevalence by region and sex (table 2) and by country and sex are shown in appendix 2 (pp 19–29). Sequelae-specific prevalence by country is shown in appendix 2 (pp 30–49).

**Table 2:** Age-standardised prevalence of ASD per 100 000 persons by region, super region, and globally, by sex, 2021

		**Total (95% UI)**	**Female (95% UI)**	**Male (95% UI)**
Global		788·3 (663·8–927·2)	508·1 (424·6–604·3)	1064·7 (898·5–1245·7)
Central Europe, eastern Europe, and central Asia		927·5 (778·5–1095·3)	644·2 (538·2–764·5)	1218·6 (1021·9–1440·2)
	Central Asia	886·0 (744·2–1044·4)	621·5 (517·0–731·8)	1154·4 (971·2–1363·8)
	Central Europe	964·4 (810·0–1140·6)	662·3 (552·2–785·0)	1263·3 (1058·4–1489·7)
	Eastern Europe	928·5 (779·8–1102·3)	645·6 (535·4–773·4)	1226·2 (1031·6–1457·2)
High-income		1090·2 (916·3–1279·3)	649·8 (543·4–771·1)	1526·5 (1283·2–1786·5)
	Australasia	1191·0 (993·0–1422·7)	709·4 (586·1–862·2)	1670·0 (1387·5–2012·1)
	High-income Asia Pacific	1559·5 (1311·3–1832·4)	938·8 (790·5–1107·1)	2161·1 (1815·5–2536·3)
	High-income north America	1097·2 (919·2–1296·7)	707·3 (589·5–838·0)	1486·9 (1244·5–1746·3)
	Southern Latin America	1056·5 (885·9–1245·4)	662·3 (548·0–787·7)	1458·7 (1216·8–1729·8)
	Western Europe	896·6 (751·6–1054·5)	477·6 (397·7–572·1)	1309·1 (1099·7–1533·1)
Latin America and Caribbean		689·5 (579·7–820·3)	464·9 (386·3–557·9)	920·2 (775·0–1087·0)
	Andean Latin America	684·3 (571·6–814·1)	464·5 (385·3–555·9)	902·4 (751·0–1069·7)
	Caribbean	682·5 (572·1–813·8)	464·7 (382·7–558·3)	902·7 (763·6–1069·7)
	Central Latin America	758·6 (639·0–897·8)	510·3 (423·1–611·4)	1017·1 (855·1–1196·5)
	Tropical Latin America	614·5 (514·7–732·3)	413·4 (340·9–495·8)	820·7 (687·6–974·5)
North Africa and Middle East		771·8 (648·5–910·7)	524·8 (436·1–624·1)	1001·3 (845·9–1179·2)
South Asia		686·2 (576·6–802·0)	466·8 (389·9–557·3)	897·0 (753·8–1038·9)
Southeast Asia, East Asia, and Oceania		669·2 (560·7–791·5)	373·6 (308·4–447·9)	950·5 (797·1–1122·2)
	East Asia	660·7 (549·4–785·9)	324·6 (267·3–391·9)	972·2 (811·3–1152·9)
	Oceania	673·2 (566·1–807·0)	433·5 (356·8–523·6)	898·2 (751·4–1078·0)
	Southeast Asia	683·0 (574·6–809·7)	458·6 (380·6–551·4)	905·9 (762·1–1069·0)
Sub-Saharan Africa		890·2 (748·6–1043·1)	628·4 (526·6–743·1)	1164·7 (980·2–1358·6)
	Central sub-Saharan Africa	885·4 (739·7–1046·5)	624·5 (517·9–742·2)	1152·0 (963·2–1364·4)
	Eastern sub-Saharan Africa	893·5 (752·4–1045·2)	629·0 (529·0–744·7)	1165·9 (980·2–1363·2)
	Southern sub-Saharan Africa	903·6 (760·1–1069·2)	637·3 (533·0–765·6)	1184·9 (995·9–1388·5)
	Western sub-Saharan Africa	886·2 (745·1–1045·6)	626·9 (524·2–740·0)	1162·9 (979·1–1363·7)

UI=Uncertainty interval. Prevalence estimates were modelled for all locations using DisMod-MR 2.1.

Autism spectrum disorder accounted for 11·5 million (95% UI 7·8–16·3) DALYs globally in 2021. Because of population growth, the DALYs attributable to autism spectrum disorder has increased from 7·9 million (5·4–11·1) DALYs globally in 1990. However, the age-standardised DALY rate remained largely unchanged between 1990 (144·5 [98·3–203·3] per 100 000 people) and 2021 (147·6 [100·2–208·2] DALYs per 100 000 people). In 2021 the age-standardised DALY rate varied significantly by sex, with 199·8 (136·3–282·0) DALYs per 100 000 males compared with 94·5 (64·5–133·0) DALYs per 100 000 females.

**Figure 3:**
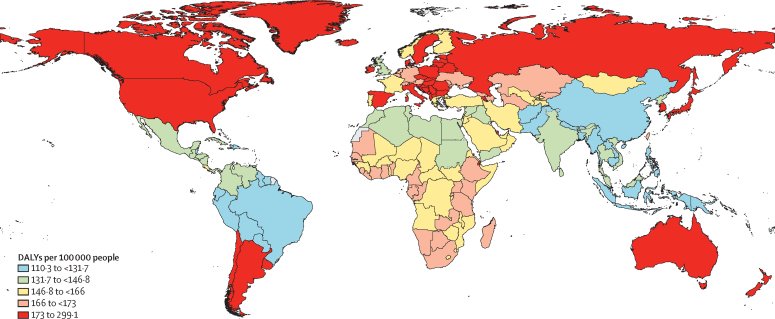
Age-standardised DALY rates per 100 000 people for autism spectrum disorder by quintile, 2021 Dotted lines indicate disputed territories. Geographical variation in DALYs were informed by location-specific prevalence estimates modelled within DisMod-MR 2.1. DALY=disability-adjusted life-year.

The age and geographical distribution of DALYs mirrored that of prevalence. DALYs emerged for children younger than age 5 years (169·2 [95% UI 115·0–237·4] DALYs per 100 000 people for children younger than 1 year) then decreased with age (163·4 [110·6–229·8] DALYs per 100 000 people younger than 20 years and 137·7 [93·9–194·5] per 100 000 people aged 20 years and older). At the super-region level, age-standardised DALY rates ranged from 126·5 (95% UI 86·0–178·0) per 100 000 people in southeast Asia, east Asia, and Oceania to 204·1 (140·7–284·7) per 100 000 people in the high-income super-region. At the region level, age-standardised DALY rates ranged between 114·4 (77·1–160·3) per 100 000 people in tropical Latin America and 293·9 (203·2–413·0) per 100 000 people in high-income Asia Pacific. The lowest age-standardised DALY rate was estimated for Bangladesh (110·3 [75·4–154·6] per 100 000 people), whereas the highest was estimated for Japan (299·1 [207·2–420·0] per 100 000 people; figure 3; appendix 2 pp 50–60).

**Figure 4:**
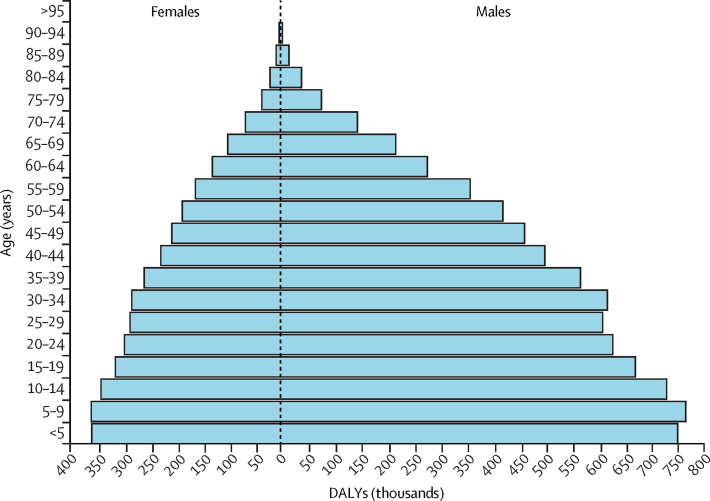
Global DALYs attributable to autism spectrum disorder, by age and sex, 2021 Blue bars represent the estimated number of DALYs (in thousands) attributable to autism spectrum disorder by age. DALY=disability-adjusted life-year.

Autism spectrum disorder was ranked 54th in DALYs in 2021 across all ages globally (0·4% [95% UI 0·3–0·6] of total DALYs). Autism spectrum disorder was the 44th leading cause of DALYs for males (0·5% [0·4–0·7]) and the 67th leading cause of DALYs for females (0·3% [0·2–0·4]). DALYs were highest in young people and decreased with age for both sexes (figure 4). Autism spectrum disorder DALYs comprised entirely YLDs. Autism spectrum disorder was ranked seventh leading cause of YLDs for children younger than 5 years (4·3% [2·7–6·5] of YLDs), eighth leading cause for children and adolescents aged 5 years to 14 years (3·4% [2·1–5·1] of YLDs), and tenth leading cause for adolescents aged 15 years to 19 years (2·2% [1·4–3·3] of YLDs). Across all ages, autism spectrum disorder was ranked 21st in YLDs (1·3% [0·8–2·0] of YLDs), was the 16th leading cause of YLDs for males (2·0% [1·3–3·0] of YLDs), and the 31st leading cause of YLDs for females (0·7% [0·5–1·1] of YLDs).

## Discussion

This Article presents global estimates of prevalence and health burden for the autism spectrum from GBD 2021, following revision to the estimation process. In 2021, one in 127 people globally were estimated to be autistic, substantially higher than the one in 271 estimated by GBD 2019. This difference is mainly attributed to the change in GBD methods, with the exclusion of studies relying on passive case finding (eg, registry or administrative prevalence estimates) that probably underestimated the prevalence of the autism spectrum.^14^ The large increase in the estimated prevalence of the autism spectrum reflects necessary improvements in its epidemiological modelling and aligns global estimates with estimates derived from high-quality epidemiological surveys.^11^, ^12^, ^13^

Despite the increase in prevalence of autism spectrum disorder in GBD 2021 compared with GBD 2019, the prevalence of autism spectrum disorder e stimated for the USA remains more conservative than findings from the US Centers for Disease Control and Prevention that indicate that one in 36 children aged 8 years in the USA were autistic in 2020.^26^ This higher prevalence was derived from a review of case notes from clinical and educational records to establish whether individuals likely met diagnostic criteria for probable autism spectrum disorder. Because individuals were not clinically evaluated for autism spectrum disorder (as is done in population diagnostic surveys), this method can overestimate the prevalence of autism spectrum disorder.

The prevalence and DALYs attributable to autism spectrum disorder in GBD 2021 were higher in males than in females, with a global age-standardised sex ratio of 2·1 to 1. The removal of prevalence data relying on passive case finding resulted in a substantial decrease in the estimated sex ratio. This result was consistent with a previous meta-analysis which showed that studies reporting registry and administrative prevalence produced a substantially higher sex ratio than studies relying on active case finding.^27^ This meta-analysis illustrated a potential sex bias for receiving diagnoses of autism spectrum disorder and, together with our findings, highlights the need for more consideration into how screening procedures and services can be altered to ensure that both autistic female and male individuals receive support. However, there are caveats to the age-standardised global sex ratio estimated by DisMod-MR 2.1. Data-rich regions tended to have higher sex ratios than regions with minimal or no data informing prevalence. For data-sparce locations (eg, in sub-Saharan Africa), the sex ratio in prevalence was more conservative, contributing to a lower sex ratio. This area is one of ongoing review as more sex-specific prevalence estimates across more geographical locations become available.

Prevalence varied substantially by region, from one in 163 people in tropical Latin America to one in 65 people in high-income Asia Pacific. The high prevalence in high-income Asia Pacific was driven by high-quality data from South Korea and Japan indicating high prevalence in this region.^12^, ^13^, ^28^, ^29^ There are many factors contributing to the geographical variation in prevalence, including varying exposure to risk factors, cultural variation in behavioural norms, validity and choice of screening and diagnostic tools, participants' responses to survey questions, or even their choice to participate.^30^, ^31^

Prevalence did not vary substantially over time. Studies reporting an increase in the prevalence of the autism spectrum have often relied on registries or administrative records to determine prevalence. Studies using random sampling or consistent active-case finding did not show this trend. This finding aligns with previous work suggesting autistic characteristics in the population have remained stable over time despite a rise in registered diagnoses.^32^ Nonetheless, the absence of temporal trends in our analysis should be interpreted with caution as we relied on a 15-year time window (reduced from 25 years) to model prevalence data. This time window might have limited our ability to explore temporal trends, but a further reduction was not possible because of data sparsity.

Autism spectrum disorder ranked within the top-ten causes of non-fatal health burden for young people (age <20 years), emphasising the need for early detection and developmental support for autistic people.^3^, ^4^, ^33^ Most epidemiological investigations into the autism spectrum have been predominantly centred on children and adolescents, leaving a gap in our understanding of the autism spectrum in adults. The prevalence and health burden of autism spectrum disorder persisted across the lifespan, beginning to decline from age 60 years. DisMod MR 2.1 estimated prevalence while also taking into consideration data available from other epidemiological parameters. In this case, with most of our raw prevalence data limited to younger cohorts, the age pattern in prevalence was informed by excess mortality data modelled by DisMod-MR 2.1 because of limited available prevalence data in adulthood. Because of limited data availability, all mortality data sourced from the systematic review relied on passive case finding (eg, from administrative records). This method might overestimate excess mortality for all autistic people, leading to an underestimation of prevalence in adulthood.

Changes in the burden of autism spectrum disorder across age highlight several considerations for how mental health services can better tailor the support available for autistic people. Given the higher burden estimated among children and adolescents, caregivers can be better supported by working closely with health professionals to monitor the development of their autistic child to jointly identify areas in which additional support is beneficial. Early intervention facilitating learning and behavioural support for young autistic children and programmes enhancing parental understanding are encouraged. School-age autistic children and adolescents could benefit from programmes addressing social communication difficulties, social skills training, or technology-based augmentative communication systems. Given the health burden of autistic spectrum disorder estimated during adulthood, autistic adults could benefit from programmes enhancing independence, such as life skills and employment training, but more research is required to identify the full range of effective services during adulthood. Despite their availability, many autistic people and their caregivers residing in high-income settings cannot access services in a timely or sustained manner, and many residing in low-income and middle-income settings cannot access these services entirely.^4^, ^34^, ^35^

There are several limitations to our estimation process that need to be considered. First, we were not able to capture all variation in the prevalence and burden of autism spectrum disorder across countries, which were often presented within large and overlapping bounds of uncertainty. Our analyses were informed by epidemiological data from 34 countries (of 204) across 12 (of 21) GBD regions. Despite additional data from the review update, this method reflects a small drop in the geographical coverage from GBD 2019 due to the removal of estimates relying on passive case finding. Including estimates relying on passive case finding would improve geographical coverage; however, accurately quantifying the bias within these estimates remains challenging because of temporal and geographical heterogeneity in autism spectrum awareness, service coverage, referral pathways, and diagnostic practices.^36^, ^37^ We strongly encourage researchers and other stakeholders to initiate population-representative diagnostic surveys and active case finding methods to investigate the prevalence of autism spectrum disorder, and to not rely solely on passive case finding methods, which we found can underestimate the prevalence of autism spectrum disorder within the population. These surveys should also investigate what proportion of autistic people are represented by passive case finding methods to help inform methods to incorporate existing data derived from passive case finding.

Second, it was difficult to quantify the amount of variation due to true differences in prevalence or due to other methodological differences between studies. We adjusted for differences in case definition and sampling methods through bias corrections, but acknowledge that these adjustments represent a small sample of the between-study variation in methods. Studies differed in screening and diagnostic tools used, tool thresholds, and methods for accounting for non-response. These are avenues for future work to better respond to remaining biases within the epidemiological data.

Third, disability weights in GBD do not vary by location and are generated by the reactions of survey participants to lay descriptions of relevant health states. A separate analysis of GBD disability weights indicated that they remained relatively stable across locations; however this work was limited to the nine countries in which disability weights surveys were done.^23^, ^24^ Additionally, for the autism spectrum, the lay descriptions used in these surveys could be considered simplistic, incomplete, and potentially stigmatising for autistic people.^38^ Many autistic people might embrace their diagnosis as an essential part of their identity and object to the view that their diagnosis is a disability or disorder. Regardless, many autistic people still require support and services, which are frequently delayed or unavailable.^4^, ^34^ Without disability weights and subsequent DALY estimates, there is a risk of inadequate prioritisation of these essential resources for autistic people. Previous work suggests that lay people from the general public provide the most balanced responses when evaluating lay descriptions of health states, as respondents tend to underestimate the disability of their health states.^23^ Future disability-weight surveys should consider generating lay descriptions and disability weights for the autism spectrum derived in consultation with autistic people to adequately capture the disability and service needs of this population.

Fourth, the levels of severity within autism spectrum disorder were derived from a meta-analysis of studies capturing the proportion of autistic people by levels of intellectual disability. This analysis only produced an overall pooled severity distribution, with insufficient data to explore variations by location, age, or sex. We expect to see variation in the severity of health loss experienced by autistic people, for instance depending on the quality and availability of care received. New processes are under development within the GBD framework to incorporate variations in health-care access globally within disease severity. We hope to apply these processes to autism spectrum disorder in future GBD cycles.

Fifth, GBD does not yet estimate prevalence and DALYs by gender (as opposed to sex). There is emerging evidence indicating greater variation in gender diversity and sexual orientation amongst autistic people.^39^ The implications of not estimating prevalence by gender on the service needs of autistic people will not be reflected within our estimates and must be considered by service planners.

Sixth, it is important to consider the health burden experienced by autistic people that is not captured in GBD 2021. The comorbidity adjustment used within GBD assumes independent comorbidity, which underestimates the comorbidity between mental disorders in which the comorbidity distribution changes depending on the combination of disorders experienced. Autistic individuals are often at an increased risk of experiencing a range of other mental and physical health conditions compared with people who are not autistic.^35^ Additionally, although YLLs for autism spectrum disorder could not be estimated via GBD methods, there is strong evidence of an elevated risk of premature mortality within autistic people, with an increased risk of self-harm and suicide compared with the general population. In a separate analysis to GBD 2021, we explored the fatal burden attributable to the elevated risk of suicide among autistic people. We estimated 13 400 excess suicide deaths among autistic people globally in 2021, equivalent to 1·8% of all suicide deaths and 621 000 excess YLLs attributable to autism spectrum disorder.^40^ Collectively, the presence of comorbid conditions and elevated risk of mortality negatively affect the health of autistic individuals, in ways in which GBD 2021 burden estimates cannot yet quantify. This limitation needs to be addressed by future research and more importantly, through increased use of prevention, early identification, and management strategies that can mitigate the effects of comorbid health conditions and reduce the risk of mortality among autistic people.

In conclusion, GBD 2021 underlined several considerations for researchers, policy makers, and communities alike as they respond to the health burden experienced by autistic people. We estimated one in 127 individuals worldwide in 2021 was autistic, placing the autism spectrum within the top-ten causes for non-fatal health burden for children and adolescents younger than 20 years. Although the importance of early detection and intervention cannot be overstated, we must also reconsider how the service needs of autistic people evolve across the lifespan. Addressing not only the needs of autistic children and adolescents, but also those of adults, who often remain under-represented in research and service provision, is imperative. With epidemiological data only available for a limited number of global regions and countries, we urge researchers to initiate more inclusive population representative diagnostic surveys with active case finding to enhance geographical coverage. We hope that this study provides a foundation for future research and policy interventions, so that key stakeholders work to ensure that the unique needs of all autistic people are met, contributing to a better, more inclusive, and more understanding future.

### Contributors

### Data sharing

To download the data used in these analyses, please visit the Global Health Data Exchange GBD 2021 website (https://ghdx.healthdata.org/gbd-2021).

## Declaration of interests

SB reports grants or contracts from the Japan Society for the Promotion of Science (JSPS), the Japanese Ministry of Education, Culture, Sports, Science and Technology, the Australian Academy of Science, Grant-in-Aid for Scientific Research (23KF0126), and JSPS International Fellowship (P23712), leadership or fiduciary roles in board, society, committee, or advocacy groups, paid or unpaid with Rotary District 9675, Sydney, Australia, the Global Health and Migration Hub Community, Global Health Hub Germany, Berlin, Germany, PLOS One, *BMC Neurology, Frontiers in Neurology, Frontiers in Stroke, Frontiers in Public Health*, the *Journal of Aging Research, Neurology International, Diagnostics*, and *BMC Medical Research Methodology*, College of Reviewers, Canadian Institutes of Health Research, Government of Canada, the World Headache Society, Bengaluru, India, the Cariplo Foundation, Milan, Italy, the National Cerebral and Cardiovascular Center, Department of Neurology, Suita, Osaka, Japan, the Cardiff University Biobank, Cardiff, UK, and the Rotary Reconciliation Action Plan, all outside the submitted work. AC reports consulting fees from Knight Therapeutics, outside the submitted work. SC reports support for the present manuscript from the National Institute for Health and Care Research, grants or contracts from the National Institute for Health and Care Research and the European Research Agency, payment or honoraria for lectures, presentations, speakers bureaus, manuscript writing, or educational events from the Association for Child and Adolescent Mental Health, the British Association of Psychopharmacology, the The Canadian ADHD Resource Alliance, and Medice, all outside the submitted work. AF reports support for the present manuscript from the National Council for Scientific and Technological Development, Brazil. IF reports financial and logistic support from the Avicenna Medical and Clinical Research Institute, outside the submitted work. LM reports support for the present manuscript from the Italian Ministry of Health (Ricerca Corrente 34/2017) and payments made to their institution. FM reports support for the present manuscript from the National Institute for Health and Care Research Doctoral Fellowship (300957). RFP-A reports payment or honoraria for lectures, presentations, speakers bureaus, manuscript writing, or educational events from Angelini, Casen-Recordati, Exeltis, Rubió, Servier, Lundbeck, Takeda, and Neuraxpharm, patents planned, issued, or pending with Angelini, Italfarmaco, Advanz Pharma, Takeda, and Lundabeck, all outside the submitted work. GP reports royalties or licences from Editora Manole, consulting fees from EMS Pharmaceuticals, Aspen, and Medice, and payment or honoraria for lectures, presentations, speakers bureaus, manuscript writing, or educational events from Ache, Abbott, Adium, Libbs, and Takeda, all outside the submitted work. AR reports other financial and non-financial support from Avicenna Medical and Clinical Research Institute, outside the submitted work. LR reports support for the present manuscript from the Italian Ministry of Health (Ricerca Corrente 34/2017) and payments made to their institution. JPS reports support for the present manuscript from the Portuguese Foundation for Science and Technology. LBS-T reports grants or contracts from the University Research Council of the Ateneo de Manila University (PhP429,500, P2,000,000, Php 597250, Php1,500,000) and Nestlé Clinical Development Unit (Php8,788,216.70), payment or honoraria for lectures, presentations, speakers bureaus, manuscript writing, or educational events from the Department of Science and Technology, Philippines, the Department of Health, Philippines, Wyeth Philippines, and Bayer Philippines, support for attending meetings or travel from the Institute of Functional Medicine to attend the 2024 Annual International Conference, leadership or fiduciary roles in board, society, committee, or advocacy groups, paid or unpaid with Philippines Society for Developmental and Behavioral Pediatrics and Circle of Hope Community Services, and stock or stock options in The Medical City, Great Valley Medical Center, OTSRI, all outside the submitted work. RT-S reports grants or contracts from the Valencian Regional Government's Ministry of Education (PROMETEO/CIPROM/2022/58) and the Spanish Ministry of Science, Innovation, and Universities (PID2021-129099OB-I00), all outside of the submitted work. ST reports grants or contracts from the Department of Health and Social Care, NHS Digital (England), leadership or fiduciary roles in board, society, committee, or advocacy groups, paid or unpaid with the Academic Secretary for the Neurodevelopmental Psychiatry Special Interest Group and Psychiatry of Intellectual Disability Faculty at the Royal College of Psychiatrists, is an Editorial Board Member for *BMC Psychiatry, Advances in Autism, Advances in Mental Health and Intellectual Disability*, and *Progress in Neurology and Psychiatry*, and is the Editor of *Psychiatry of Intellectual Disability Across Cultures* (Oxford University Press), all outside the submitted work. MZ is an AstraZeneca employee. All other authors declare no competing interests. The views expressed in this Article are those of the authors and not necessarily those of UK National Health Service (NHS), National Institute for Health and Care Research, or the Department of Health and Social Care.
