# Supplementary material for: The global epidemiology and health burden of the autism spectrum: findings from the Global Burden of Disease Study 2021
Source: Lancet Psychiatry. 2025 Feb;12(2):111–21. doi: 10.1016/S2215-0366(24)00363-8 (PMC11750762; doi:10.1016/S2215-0366(24)00363-8)
Supplement: Supplementary appendix 1 [file mmc1.pdf]

THE LANCET
Psychiatry

Supplementary appendix 1

This appendix formed part of the original submission and has been peer reviewed. We post it as supplied by the authors.

Supplement to: Global Burden of Disease Study 2021 Autism Spectrum Collaborators. The global epidemiology and health burden of the autism spectrum: findings from the Global Burden of Disease Study 2021. *Lancet Psychiatry* 2024; published online Dec 19. https://doi.org/10.1016/S2215-0366(24)00363-8.

Appendix 1: Authorship appendix to "The global epidemiology and health burden of the autism spectrum: findings from the Global Burden of Disease Study 2021"

This appendix provides further authorship detail for "The global epidemiology and health burden of the autism spectrum: findings from the Global Burden of Disease Study 2021"

Table of Contents

Appendix 1: Authorship appendix to "The global epidemiology and health burden of the autism spectrum: findings from the Global Burden of Disease Study 2021" 1

GBD 2021 Autism Spectrum Collaborators 2

Affiliations 2

Authors' Contributions 8

- Managing the overall research enterprise 8
- Writing the first draft of the manuscript 8
- Primary responsibility for applying analytical methods to produce estimates 8
- Primary responsibility for seeking, cataloguing, extracting, or cleaning data; designing or coding figures and tables 8
- Providing data or critical feedback on data sources 8
- Developing methods or computational machinery 8
- Providing critical feedback on methods or results 8
- Drafting the work or revising it critically for important intellectual content 9
- Managing the estimation or publications process 10

GBD 2021 Autism Spectrum Collaborators

Damian F Santomauro, Holly E Erskine, Ana M Mantilla Herrera, Paul Anthony Miller, Jamileh Shadid, Hailey Hagins, Isaac Yeboah Addo, Qorinah Estiningtyas Sakilah Adnani, Bright Opoku Ahinkorah, Ayman Ahmed, Fadwa Naji Alhalaiqa, Mohammed Usman Ali, Sabah Al-Marwani, Joseph Uy Almazan, Sami Almustanyir, Farrukh Jawad Alvi, Yasser Sami Abdel Dayem Amer, Edward Kwabena Ameyaw, Sohrab Amiri, Catalina Liliana Andrei, Dhanalakshmi Angappan, Catherine M Antony, Aleksandr Y Aravkin, Tahira Ashraf, Jose L Ayuso-Mateos, Amadou Barrow, Kavita Batra, Maryam Bemanalizadeh, Akshaya Srikanth Bhagavathula, Sonu Bhaskar, Jasvinder Singh Singh Bhatti, Srinivasa Rao Bolla, Gabrielle Britton, Joao Mauricio Castaldelli-Maia, Ferrán Catalá-López, Arthur Caye, Vijay Kumar Chattu, Yuen Yu Chong, Liliana G Ciobanu, Samuele Cortese, Natalia Cruz-Martins, Berihun Assefa Dachew, Xiaochen Dai, Amira Hamed Darwish, Mohsen Dashti, Alejandro de la Torre-Luque, Daniel Diaz, Delaney D Ding, Angel Belle Cheng Dy, Arkadiusz Marian Dziedzic, Sepideh Ebrahimi Meimand, Omar Abdelsadek Abdou El Meligy, Iman El Sayed, Frank J Elgar, Adeniyi Francis Fagbamigbe, Pawan Sirwan Faris, Andre Faro, Nuno Ferreira, Irina Filip, Florian Fischer, Aravind P Gandhi, Balasankar Ganesan, Miglas Welay Gebregergis, Mesfin Gebrehiwot, Bardiya Ghaderi Yazdi, Mohammad-Reza Ghasemi, Afsaneh Ghasemzadeh, Sasidhar Gunturu, Veer Bala Gupta, Vivek Kumar Gupta, Sobia Ahsan Halim, Brian J Hall, Fulei Han, Josep Maria Haro, Ahmed I Hasaballah, Simon I Hay, Darren Hedley, Bartosz Helfer, Md Mahbub Hossain, Bing-Fang Hwang, Umar Idris Ibrahim, Mehran Ilaghi, Md Rabiul Islam, Sheikh Mohammed Shariful Islam, Mahalaxmi Iyer, Khushleen Jaggi, Haitham Jahrami, Elham Jamshidi, Ali Khaleghi, Abdul Aziz Khan, Mohammad Jobair Khan, Feriha Fatima Khidri, Kwanghyun Kim, Hyun Yong Koh, Manasi Kumar, Iván Landires, Long Khanh Dao Le, Seung Won Lee, Zhihui Li, Stephen S Lim, Jose Martinez-Raga, Roy Rillera Marzo, Indu Liz Matthew, Andrea Maugeri, Tomislav Mestrovic, Philip B Mitchell, Salahuddin Mohammed, Ali H Mokdad, Lorenzo Monasta, Fateme Montazeri, Matías Mrejen, Faraz Mughal, Christopher J L Murray, Woojae Myung, Javaid Nauman, Charles Richard James Newton, Huong Lan Thi Nguyen, Chisom Adaobi Nri-Ezedi, Vincent Ebuka Nwatah, Adeolu Olufunso Oladunjoye, Isaac Iyinoluwa Olufadewa, Michal Ordak, Nikita Otstavnov, Raul Felipe Palma-Alvarez, Romil R Parikh, Seoyeon Park, Maja Pasovic, Jay Patel, Marcos Pereira, Maria Odete Pereira, Michael R Phillips, Guilherme V Polanczyk, Mohammad Pourfridoni, Jagadeesh Puvvula, Amir Radfar, Fakher Rahim, Mosiur Rahman, Muhammad Aziz Rahman, Amir Masoud Rahmani, Masoud Rahmati, Zubair Ahmed Ratan, Taeho Gregory Rhee, Luca Ronfani, Priyanka Roy, Basema Ahmad Saddik, Amene Saghazadeh, Joseph W Sakshaug, Sana Salehi, Vijaya Paul Samuel, Senthilkumar Sankararaman, Aswini Saravanan, Maheswar Satpathy, Austin E Schumacher, David C Schwebel, Mario Šekerija, Arman Shafiee, Saeed Shahabi, Muhammad Aaqib Shamim, João Pedro Silva, Yonatan Solomon, Lourdes Bernadette C Sumpaico-Tanchanco, Chandan Kumar Swain, Rafael Tabarés-Seisdedos, Mohamad-Hani Temsah, Samuel Joseph Tromans, Lilian Tzivian, Ravi Prasad Varma, Andres Fernando Vinueza Veloz, Maria Fernanda Vinueza Veloz, Mandaras Tariku Walde, Muhammad Waqas, Nuwan Darshana Wickramasinghe, Renjulal Yesodharan, Dong Keon Yon, Yoosik Youm, Burhan Abdullah Zaman, Youjie Zeng, Magdalena Zielińska, Harvey A Whiteford, Traolach Brugha, James G Scott, Theo Vos, and Alize J Ferrari.

Affiliations

West Moreton Hospital Health Services (A M Mantilla Herrera PhD), Queensland Centre for Mental Health Research, Wacol, QLD, Australia (D F Santomauro PhD, H E Erskine PhD, P A Miller PhD, J Shadid BSc, Prof H A Whiteford PhD, A J Ferrari PhD); School of Public Health (D F Santomauro PhD, H E Erskine PhD, A M Mantilla Herrera PhD, P A Miller PhD, J Shadid BSc, Prof H A Whiteford PhD, A J Ferrari PhD),

The University of Queensland, Brisbane, QLD, Australia; Institute for Health Metrics and Evaluation (D F Santomauro PhD, P A Miller PhD, J Shadid BSc, H Hagins MSPH, C M Antony MA, A Y Aravkin PhD, X Dai PhD, Prof S I Hay FMedSci, Prof S S Lim PhD, T Mestrovic PhD, Prof A H Mokdad PhD, Prof C J L Murray DPhil, M Pasovic Med, A E Schumacher PhD, Prof H A Whiteford PhD, Prof T Vos PhD, A J Ferrari PhD), Department of Applied Mathematics (A Y Aravkin PhD), Department of Health Metrics Sciences, School of Medicine (A Y Aravkin PhD, X Dai PhD, Prof S I Hay FMedSci, Prof S S Lim PhD, Prof A H Mokdad PhD, Prof C J L Murray DPhil, Prof T Vos PhD), University of Washington, Seattle, WA, USA; School of Medicine (I Y Addo PhD), Sydney Medical School (S Islam PhD), University of Sydney, Sydney, NSW, Australia; Centre for Social Research in Health (I Y Addo PhD), School of Psychiatry (Prof P B Mitchell MD), School of Population Health (Prof B A Saddik PhD), University of New South Wales, Sydney, NSW, Australia; Department of Public Health (Q Adnani PhD), Universitas Padjadjaran (Padjadjaran University), Bandung, Indonesia; School of Public Health (B O Ahinkorah MPhil), University of Technology Sydney, Sydney, NSW, Australia; Institute of Endemic Diseases (A Ahmed MSc), University of Khartoum, Khartoum, Sudan; Swiss Tropical and Public Health Institute (A Ahmed MSc), University of Basel, Basel, Switzerland; College of Nursing (Prof F N Alhalaiqa PhD), Qatar University, Doha, Qatar; Department of Medical Rehabilitation (Physiotherapy) (M U Ali MSc), University of Maiduguri, Maiduguri, Nigeria; Department of Rehabilitation Sciences (M U Ali MSc, M Khan MPH), Hong Kong Polytechnic University, Hong Kong, China; Independent Consultant, Amman, Jordan (S Al-Marwani MSc); Department of Medicine (J U Almazan PhD), Department of Biomedical Sciences (S Bolla PhD), Nazarbayev University, Astana, Kazakhstan; College of Medicine (S Almustanyir MD), Alfaisal University, Riyadh, Saudi Arabia; Ministry of Health, Riyadh, Saudi Arabia (S Almustanyir MD); University Institute of Public Health (F J Alvi MPH), University Institute of Radiological Sciences and Medical Imaging Technology (T Ashraf MS), The University of Lahore, Lahore, Pakistan; Quality Management Department (Y S A Amer MSc), Pediatric Intensive Care Unit (Prof M Temsah MD), King Saud University, Riyadh, Saudi Arabia; Alexandria Center for Evidence-based Clinical Practice Guidelines (Y S A Amer MSc), Pediatric Dentistry and Dental Public Health Department (Prof O A A El Meligy PhD), Biomedical Informatics and Medical Statistics Department (I El Sayed PhD), Alexandria University, Alexandria, Egypt; School of Graduate Studies (E K Ameyaw MPhil), Lingnan University, Hong Kong, China; Spiritual Health Research Center (S Amiri PhD), Baqiyatallah University of Medical Sciences, Tehran, Iran; Department of Cardiology (Prof C Andrei PhD), Carol Davila University of Medicine and Pharmacy, Bucharest, Romania; Department of Child Neurology (D Angappan MD), Oregon Health and Science University, Portland, OR, USA; Department of Psychiatry (Prof J L Ayuso-Mateos PhD), Universidad Autónoma de Madrid (Autonomous University of Madrid), Madrid, Spain; Centre for Biomedical Research in Mental Health Network (CIBERSAM) (Prof J L Ayuso-Mateos PhD, F Catalá-López PhD), Department of Mental Health (J M Haro MD) Institute of Health Carlos III (Prof R Tabarés-Seisdedos PhD), Madrid, Spain; Department of Public and Environmental Health (A Barrow MPH), University of The Gambia, Banjul, The Gambia; Department of Epidemiology (A Barrow MPH, D D Ding BS), University of Florida, Gainesville, FL, USA; Department of Medical Education (K Batra PhD), University of Nevada Las Vegas, Las Vegas, NV, USA; Department of Pediatrics (M Bemanalizadeh MD), Isfahan University of Medical Sciences, Isfahan, Iran; Department of Pediatric Neurology (M Bemanalizadeh MD), Medical school (S Ebrahimi Meimand MD), Department of Neurology (B Ghaderi Yazdi MD), Psychiatry and Psychology Research Center (A Khaleghi PhD), Research Center for Immunodeficiencies (A Saghazadeh MD), Tehran University of Medical Sciences, Tehran, Iran (M Pourfridoni MD); Department of Public Health (A S Bhagavathula PhD), North Dakota State University, Fargo, ND, USA; Division of Gastroenterology and Hepatology (A S Bhagavathula PhD), Mayo Clinic,

Jacksonville, FL, USA; Global Health Neurology Lab (S Bhaskar MD), NSW Brain Clot Bank, Sydney, NSW, Australia; Division of Cerebrovascular Medicine and Neurology (S Bhaskar MD), National Cerebral and Cardiovascular Center, Suita, Japan; Department of Human Genetics and Molecular Medicine (Prof J S Bhatti PhD), Department of Microbiology (M Iyer PhD), Central University of Punjab, Bathinda, India; Center for Neuroscience (G Britton PhD), Institute for Scientific Research and High Technology Services, Panama City, Panama; Gorgas Memorial Institute for Health Studies, Panama City, Panama (G Britton PhD); Department of Psychiatry (Prof J Castaldelli-Maia PhD, A Caye PhD), University of São Paulo, São Paulo, Brazil; Center for Human and Social Sciences (CCHS) (F Catalá-López PhD), Spanish National Research Council (CSIC), Madrid, Spain; Department of Psychiatry (A Caye PhD), Federal University of Rio Grande do Sul, Porto Alegre, Brazil; Temerty Faculty of Medicine (V Chattu MD), University of Toronto, Toronto, ON, Canada; Department of Community Medicine (V Chattu MD), Datta Meghe Institute of Medical Sciences, Sawangi, India; The Nethersole School of Nursing (Y Chong PhD), The Chinese University of Hong Kong, Hong Kong, China; Adelaide Medical School (L G Ciobanu PhD), University of Adelaide, Adelaide, SA, Australia; School of Pharmacy and Medical Sciences (L G Ciobanu PhD), University of South Australia, Adelaide, SA, Australia; School of Psychology (Prof S Cortese PhD), University of Southampton, Southampton, UK; Department of Child and Adolescent Psychiatry (Prof S Cortese PhD), Institute for Excellence in Health Equity (M Kumar PhD), New York University, New York, NY, USA; Department of Diagnostic and Therapeutic Technologies (Prof N Cruz-Martins PhD), Cooperativa de Ensino Superior Politécnico e Universitário (Polytechnic and University Higher Education Cooperative), Vila Nova de Famalicão, Portugal; Institute for Research and Innovation in Health (i3S) (Prof N Cruz-Martins PhD), Research Unit on Applied Molecular Biosciences (UCIBIO) (J Silva PhD), University of Porto, Porto, Portugal; School of Public Health (B A Dachew PhD), Curtin University, Perth, WA, Australia; Department of Epidemiology (B A Dachew PhD), University of Gondar, Gondar, Ethiopia; Department of Pediatrics (A H Darwish MD), Tanta University, Tanta, Egypt; Immunology Research Center (M Dashti MD, A Ghasemzadeh MD), Tabriz University of Medical Sciences, Tabriz, Iran; Department of Legal Medicine, Psychiatry and Pathology (A de la Torre-Luque PhD), Universidad Complutense de Madrid (Complutense University of Madrid), Madrid, Spain; Faculty of Science (Prof D Diaz PhD), National Autonomous University of Mexico, Mexico City, Mexico; Ateneo Center for Research and Innovation (A C Dy MD), Center for Research and Innovation (L C Sumpaico-Tanchanco MD), Ateneo De Manila University, Pasig City, Philippines; Demography and Health (A C Dy MD), London School of Hygiene & Tropical Medicine, London, UK; Department of Conservative Dentistry with Endodontics (A M Dziedzic DSc), Medical University of Silesia, Katowice, Poland; Department of Pediatric Dentistry (Prof O A A El Meligy PhD), King Abdulaziz University, Jeddah, Saudi Arabia; School of Population and Global Health (Prof F J Elgar PhD), McGill University, Montreal, QC, Canada; Department of Epidemiology and Medical Statistics (A F Fagbamigbe PhD), Faculty of Public Health (I I Olufadewa MHS), University of Ibadan, Ibadan, Nigeria; Research Centre for Healthcare and Community (A F Fagbamigbe PhD), Coventry University, Coventry, UK; Department of Biology (P S Faris PhD), Salahaddin University-Erbil, Erbil, Iraq; Department of Biology (P S Faris PhD), Cihan University-Erbil, Erbil, Iraq; Department of Psychology (A Faro PhD), Federal University of Sergipe, São Cristóvão, Brazil; Department of Social Sciences (Prof N Ferreira PhD), University of Nicosia, Nicosia, Cyprus; Department of Psychiatry (I Filip MD), Kaiser Permanente, Mission Viejo, CA, USA; School of Health Sciences (I Filip MD), A.T. Still University, Mesa, AZ, USA; Institute of Public Health (F Fischer PhD), Charité Medical University Berlin, Berlin, Germany; Department of Community Medicine and Family Medicine (A P Gandhi MD), All India Institute of Medical Sciences, Nagpur, India; Institute of Health and Wellbeing (B Ganesan PhD),

Federation University Australia, Churchill, VIC, Australia; Department of Midwifery (M W Gebregergis MSc), Adigrat University, Adigrat, Ethiopia; Department of Environmental Health (M Gebrehiwot DSc), Wollo University, Dessie, Ethiopia; Department of Medical Genetics (M Ghasemi PhD), Center for Comprehensive Genetic Services (M Ghasemi PhD), Shahid Beheshti University of Medical Sciences, Tehran, Iran; Department of Psychiatry (S Gunturu MD), Bronxcare Health System, Bronx, NY, USA; Department of Psychiatry (S Gunturu MD), Icahn School of Medicine at Mount Sinai, New York, NY, USA; School of Medicine (V Gupta PhD), Deakin University, Geelong, VIC, Australia; Faculty of Medicine Health and Human Sciences (Prof V K Gupta PhD), Macquarie University, Sydney, NSW, Australia; Natural and Medical Sciences Research Center (S A Halim PhD), University of Nizwa, Nizwa, Oman; NYU Shanghai, Shanghai, China (B J Hall PhD); School of Public Health (F Han MS), Qingdao University, Qingdao, China; Research Unit (J M Haro MD), Parc Sanitari Sant Joan de Deu, Barcelona, Spain; Department of Zoology and Entomology (A I Hasaballah PhD), Al-Azhar University, Cairo, Egypt; Psychology and Public Health (D Hedley PhD), School of Nursing and Midwifery (Prof M Rahman PhD), La Trobe University, Melbourne, VIC, Australia; Institute of Psychology (B Helfer PhD), University of Wroclaw, Wroclaw, Poland; Meta Research Centre (B Helfer PhD), University of Wrocław, Wroclaw, Poland; Department of Decision and Information Sciences (M Hossain DrPH), University of Houston, Houston, TX, USA; Public Health Research Group (M Hossain DrPH), Nature Study Society of Bangladesh, Khulna, Bangladesh; Department of Occupational Safety and Health (Prof B Hwang PhD), China Medical University, Taiwan, Taichung, Taiwan; Department of Occupational Therapy (Prof B Hwang PhD), Asia University, Taiwan, Taichung, Taiwan; Faculty of Pharmacy, Sultan Zainal Abidin University, Malaysia (U I Ibrahim PhD), Sultan Zainal Abidin University, Malaysia, Terengganu, Malaysia; Neurology Research Center (M Ilaghi MD), Kerman Neuroscience Research Center (M Ilaghi MD), Kerman University of Medical Sciences, Kerman, Iran; School of Pharmacy (M Islam PhD), BRAC University, Dhaka, Bangladesh; Institute for Physical Activity and Nutrition (S Islam PhD), Deakin University, Burwood, VIC, Australia; Department of Nephrology (K Jaggi MD), San Mateo Medical Center, San Mateo, CA, USA; Department of Nephrology (K Jaggi MD), Mills Peninsula Medical Center, Burlingame, CA, USA; College of Medicine and Medical Sciences (H Jahrami PhD), Arabian Gulf University, Manama, Bahrain; Ministry of Health, Manama, Bahrain (H Jahrami PhD); Johns Hopkins University, Baltimore, MD, USA (E Jamshidi PharmD); Bio-X Institute, School of Life Sciences (A Khan MS), Shanghai Jiao Tong University, P.R. China, Minhang, China; Department of Animal Sciences (A Khan MS), Quaid-i-Azam University, Islamabad, Islamabad, Pakistan; Department of Biochemistry (F Khidri PhD), Liaquat University Of Medical and Health Sciences, Jamshoro, Pakistan; Graduate School of Public Health (K Kim PhD), Yonsei University, Busan, South Korea; Department of Neurology (H Koh PhD), Baylor College of Medicine, Houston, TX, USA; Department of Psychiatry (M Kumar PhD), University of Nairobi, Nairobi, Kenya; Unidad de Genética y Salud Pública (Prof I Landires MD), Instituto de Ciencias Médicas, Las Tablas, Panama; Ministry of Health (Prof I Landires MD), Hospital Joaquín Pablo Franco Sayas, Las Tablas, Panama; Health Economics Division (L K D Le PhD), Monash University, Burwood, VIC, Australia; Department of Precision Medicine (Prof S Lee MD), Sungkyunkwan University, Suwon-si, South Korea; Tsinghua Vanke School of Public Health (Z Li PhD), Tsinghua University, Beijing, China; Department of Global Health and Population (Z Li PhD), Harvard University, Boston, MA, USA; Psychiatry Department (J Martinez-Raga PhD), Hospital Universitario Doctor Peset, Valencia, Spain; Department of Medicine (J Martinez-Raga PhD, Prof R Tabarés-Seisdedos PhD), University of Valencia, Valencia, Spain; Faculty of Humanities and Health Sciences (Prof R R Marzo MD), Curtin University, Sarawak, Malaysia; Jeffrey Cheah School of Medicine and Health Sciences (Prof R R Marzo MD), Monash University, Subang Jaya, Malaysia; Pediatrics (I L

Matthew MD), Emory University, Atlanta, GA, USA; Department of Medical and Surgical Sciences and Advanced Technologies "GF Ingrassia" (A Maugeri PhD), University of Catania, Catania, Italy; University Centre Varazdin (T Mestrovic PhD), University North, Varazdin, Croatia; Department of Pharmaceutical Sciences (S Mohammed PhD), Notre Dame of Maryland University, Baltimore, MD, USA; Department of Pharmacy (S Mohammed PhD), Mizan-Tepi University, Mizan, Ethiopia; Clinical Epidemiology and Public Health Research Unit (L Monasta DSc, L Ronfani PhD), Burlo Garofolo Institute for Maternal and Child Health, Trieste, Italy; Department of Ophthalmology & Vision Science (F Montazeri MD), University of California Davis, Sacramento, CA, USA; Non-Communicable Diseases Research Center (NCDRC), Tehran, Iran (F Montazeri MD); Department of Economics (M Mrejen PhD), Fluminense Federal University, Niterói - Rio de Janeiro, Brazil; School of Medicine (F Mughal FRCGP), Keele University, Keele, UK; Division of Psychology and Mental Health (F Mughal FRCGP), University of Manchester, Manchester, UK; Department of Psychiatry (W Myung PhD), Seoul National University, Seoul, South Korea; Department of Neuropsychiatry (W Myung PhD), Seoul National University Bundang Hospital, Seongnam, South Korea; College of Medicine and Health Sciences (J Nauman PhD), United Arab Emirates University, Al Ain, United Arab Emirates; Department of Circulation and Medical Imaging (J Nauman PhD), Norwegian University of Science and Technology, Trondheim, Norway; Department of Psychiatry (Prof C R J Newton MD), University of Oxford, Oxford, UK; Department of Neurosciences (Prof C R J Newton MD), Kenya Medical Research Institute/Wellcome Trust Research Programme, Kilifi, Kenya; Institute for Global Health Innovations (H L T Nguyen MPH), Duy Tan University, Hanoi, Viet Nam; Department of Paediatrics (C A Nri-Ezedi PhD), Nnamdi Azikiwe University, Awka, Nigeria; Department of Pediatrics (V E Nwatah MD), National Hospital Abuja, Abuja, Nigeria; Department of International Public Health (V E Nwatah MD), University of Liverpool, Liverpool, UK; Department of Psychiatry and Behavioral Sciences (A O Oladunjoye MD), Baylor College of Medicine, Houston, FL, USA; Medicine Critical Care (A O Oladunjoye MD), Boston Children's Hospital, Boston, USA; Slum and Rural Health Initiative Research Academy (I I Olufadewa MHS), Slum and Rural Health Initiative, Ibadan, Nigeria; Department of Pharmacotherapy and Pharmaceutical Care (M Ordak PhD), Department of Biochemistry and Pharmacogenomics (M Zielińska MPharm), Medical University of Warsaw, Warsaw, Poland; Laboratory of Public Health Indicators Analysis and Health Digitalization (N Otstavnov BA), Moscow Institute of Physics and Technology, Dolgoprudny, Russia; Department of Mental Health (R F Palma-Alvarez PhD), Hospital Universitari Vall d'Hebron (CIBERSAM), Barcelona, Spain; Biomedical Network Research Centre on Mental Health (CIBERSAM), Barcelona, Spain (R F Palma-Alvarez PhD); Department of Epidemiology and Community Health (R R Parikh MD), University of Minnesota, Minneapolis, MN, USA; Department of Biomedical Data Science (S Park MD), Stanford University, Stanford, CA, USA; Global Health Governance Programme (J Patel BSc), University of Edinburgh, Edinburgh, UK; School of Dentistry (J Patel BSc), University of Leeds, Leeds, UK; Institute of Collective Health (Prof M Pereira PhD), Federal University of Bahia, Salvador, Brazil; Department of Applied Nursing (Prof M O Pereira PhD), Federal University of Minas Gerais, Belo Horizonte, Brazil; Shanghai Mental Health Center (Prof M R Phillips MD), Shanghai Jiao Tong University, Shanghai, China; Departments of Psychiatry and Epidemiology (Prof M R Phillips MD), Columbia University, New York, NY, USA; Department of Psychiatry (Prof G V Polanczyk MD), University of Sao Paulo, São Paulo, Brazil; Department of Biostatistics, Epidemiology, and Informatics (J Puvvula PhD), University of Pennsylvania, Philadelphia, PA, USA; College of Medicine (A Radfar MD), University of Central Florida, Orlando, FL, USA; Avicenna Medical and Clinical Research Institute, Encino, CA, USA (A Radfar MD); PhD in Medical biotechnology (Prof F Rahim PhD), Osh state university, Osh, Kyrgyzstan; Director of Central Asia Research Collaboration Group (Prof F Rahim PhD), Asfendiyarov

Kazakh National Medical University, Almaty, Kazakhstan; Department of Population Science and Human Resource Development (Prof M Rahman DrPH), University of Rajshahi, Rajshahi, Bangladesh; Institute of Health and Wellbeing (Prof M Rahman PhD), Federation University Australia, Berwick, VIC, Australia; Future Technology Research Center (A Rahmani PhD), National Yunlin University of Science and Technology, Yunlin, Taiwan; Health Service Research and Quality of Life Center (CEReSS) (Prof M Rahmati PhD), Aix-Marseille University, Marseille, France; Department of Biomedical Engineering (Z Ratan MSc), Khulna University of Engineering and Technology, Khulna, Bangladesh; School of Health and Society (Z Ratan MSc), University of Wollongong, Wollongong, NSW, Australia; Department of Public Health Sciences (T Rhee PhD), University of Connecticut, Farmington, CT, USA; Department of Psychiatry (T Rhee PhD), Yale University, New Haven, CT, USA; Department of Labour (P Roy PhD), Government of West Bengal, Kolkata, India; College of Medicine (Prof B A Saddik PhD), University of Sharjah, Sharjah, United Arab Emirates; LMU-Munich, Munich, Germany (J W Sakshaug PhD); Institute for Employment Research, Nuremberg, Germany (J W Sakshaug PhD); Mark and Mary Stevens Neuroimaging and Informatics Institute (S Salehi MD), University of Southern California, Los Angeles, CA, USA; Department of Anatomy (Prof V P Samuel PhD), Ras Al Khaimah Medical and Health Sciences University, Ras Al Khaimah, United Arab Emirates; Department of Pediatrics (S Sankararaman MD), University Hospitals Rainbow Babies & Children's Hospital, Cleveland, OH, USA; Department of Pediatrics (S Sankararaman MD), Case Western Reserve University, Cleveland, OH, USA; Department of Pharmacology (A Saravanan MD, M Shamim MBBS), All India Institute of Medical Sciences, Jodhpur, India; Indira Gandhi Medical College and Research Institute, Puducherry, India (A Saravanan MD); UGC Centre of Advanced Study in Psychology (Prof M Satpathy PhD), Department of Analytical and Applied Economics (C Swain MPhil), Utkal University, Bhubaneswar, India; Udyam-Global Association for Sustainable Development, Bhubaneswar, India (Prof M Satpathy PhD); Department of Psychology (D C Schwebel PhD), University of Alabama at Birmingham, Birmingham, AL, USA; Department of Medical Statistics (M Šekerija PhD), University of Zagreb, Zagreb, Croatia; Department of Epidemiology and Prevention of Chronic Noncommunicable Diseases (M Šekerija PhD), Croatian Institute of Public Health, Zagreb, Croatia; Non-communicable Diseases Research Center (A Shafiee MD), Alborz University of Medical Sciences, Karaj, Iran; Health Policy Research Center (S Shahabi PhD), Shiraz University of Medical Sciences, Shiraz, Iran; Department of Nursing (Y Solomon MSc), Dire Dawa University, Dire Dawa, Ethiopia; Department of Health Sciences (S J Tromans PhD, Prof T Brugha MD), University of Leicester, Leicester, UK; Adult Learning Disability Service (S J Tromans PhD), Leicestershire Partnership National Health Service Trust, Leicester, UK; Institute of Clinical and Preventive Medicine (Prof L Tzivian PhD), University of Latvia, Riga, Latvia; Achutha Menon Centre for Health Science Studies (R P Varma MD), Sree Chitra Tirunal Institute for Medical Sciences and Technology, Trivandrum, India; Pograma de doctorado IPK (A Vinueza Veloz MSc), Institute of Tropical Medicine, La Habana, Cuba; Community Medicine and Global Health (M Vinueza Veloz PhD), Oslo University, Oslo, Norway; Department of Neuroscience (M Vinueza Veloz PhD), Erasmus University Medical Center, Rotterdam, Netherlands; Department of Psychiatry (M T Walde MSc), Haramaya University, Harar, Ethiopia; Key Laboratory of Computer-Aided Drug Design (M Waqas PhD), Guangdong Medical University, Dongguan, China; Department of Biotechnology and Genetic Engineering (M Waqas PhD), Hazara University Mansehra, Mansehra, Pakistan; Department of Community Medicine (N D Wickramasinghe MD), Rajarata University of Sri Lanka, Anuradhapura, Sri Lanka; Manipal College of Nursing (R Yesodharan MSc), Manipal Academy of Higher Education, Udupi, India; Department of Pediatrics (Prof D Yon MD), Kyung Hee University, Seoul, South Korea; Department of Sociology (Prof Y Youm PhD), Yonsei University, Seoul, South Korea; Basic Sciences Department (B A

Zaman PhD), University of Duhok, Duhok, Iraq; Department of Anesthesiology (Y Zeng MD), Third Xiangya Hospital of Central South University, Changsha, China; Child and Youth Mental Health Service (CYMHS) (Prof J G Scott PhD), Children's Health Queensland Hospital and Health Service, South Brisbane, QLD, Australia.

Authors' Contributions

Authors' contributions for Global Burden of Disease papers are listed in alphabetical order.

Managing the overall research enterprise

Alize J Ferrari, Damian F Santomauro, Christopher J L Murray, Theo Vos, and Harvey A Whiteford.

Writing the first draft of the manuscript

Alize J Ferrari and Damian F Santomauro.

Primary responsibility for applying analytical methods to produce estimates

Damian F Santomauro.

Primary responsibility for seeking, cataloguing, extracting, or cleaning data; designing or coding figures and tables

Holly E Erskine, Ana M Mantilla Herrera, Paul A Miller, Damian F Santomauro, and Jamileh Shadid.

Providing data or critical feedback on data sources

Qorinah Estiningtyas Sakilah Adnani, Bright Opoku Ahinkorah, Ayman Ahmed, Joseph Uy Almazan, Sami Almustanyir, Edward Kwabena Ameyaw, Tahira Ashraf, Jose L Ayuso-Mateos, Akshaya Srikanth Bhagavathula, Sonu Bhaskar, Jasvinder Singh Singh Bhatti, Gabrielle Britton, Traolach Brugha, Joao Mauricio Castaldelli-Maia, Ferrán Catalá-López, Natalia Cruz-Martins, Xiaochen Dai, Alejandro de la Torre-Luque, Iman El Sayed, Adeniyi Francis Fagbamigbe, Alize J Ferrari, Bardiya Ghaderi Yazdi, Vivek Kumar Gupta, Brian J Hall, Josep Maria Haro, Simon I Hay, Darren Hedley, Sheikh Mohammed Shariful Islam, Mahalaxmi Iyer, Khushleen Jaggi, Haitham Jahrami, Feriha Fatima Khidri, Manasi Kumar, Long Khanh Dao Le, Seung Won Lee, Stephen S Lim, Ana M Mantilla Herrera, Roy Rillera Marzo, Indu Liz Matthew, Andrea Maugeri, Paul A Miller, Salahuddin Mohammed, Ali H Mokdad, Faraz Mughal, Christopher J L Murray, Charles Richard James Newton, Huong Lan Thi Nguyen, Adeolu Olufunso Oladunjoye, Romil R Parikh, Maja Pasovic, Maria Odete Pereira, Jagadeesh Puvvula, Fakher Rahim, Amir Masoud Rahmani, Zubair Ahmed Ratan, Taeho Gregory Rhee, Luca Ronfani, Priyanka Roy, Basema Ahmad Saddik, Vijaya Paul Samuel, Damian F Santomauro, Maheswar Satpathy, James G Scott, Jamileh Shadid, Arman Shafiee, Muhammad Aaqib Shamim, Yonatan Solomon, Lourdes Bernadette C Sumpaico-Tanchanco, Chandan Kumar Swain, Rafael Tabarés-Seisdedos, Andres Fernando Vinueza Veloz, Mandaras Tariku Walde, and Harvey A Whiteford.

Developing methods or computational machinery

Aleksandr Y Aravkin, Xiaochen Dai, Alize J Ferrari, Simon I Hay, Paul A Miller, Ali H Mokdad, Christopher J L Murray, Maja Pasovic, Damian F Santomauro, Austin E Schumacher, and Theo Vos.

Providing critical feedback on methods or results

Isaac Yeboah Addo, Qorinah Estiningtyas Sakilah Adnani, Bright Opoku Ahinkorah, Ayman Ahmed, Mohammed Usman Ali, Sabah Al-Marwani, Joseph Uy Almazan, Sami Almustanyir, Farrukh Jawad Alvi, Yasser Sami Abdel Dayem Amer, Edward Kwabena Ameyaw, Sohrab Amiri, Catalina Liliana Andrei, Tahira

Ashraf, Jose L Ayuso-Mateos, Amadou Barrow, Kavita Batra, Maryam Bemanalizadeh, Akshaya Srikanth Bhagavathula, Sonu Bhaskar, Jasvinder Singh Singh Bhatti, Gabrielle Britton, Traolach Brugha, Joao Mauricio Castaldelli-Maia, Ferrán Catalá-López, Vijay Kumar Chattu, Yuen Yu Chong, Samuele Cortese, Natalia Cruz-Martins, Berihun Assefa Dachew, Xiaochen Dai, Amira Hamed Darwish, Mohsen Dashti, Alejandro de la Torre-Luque, Daniel Diaz, Delaney D Ding, Angel Belle Cheng Dy, Arkadiusz Marian Dziedzic, Sepideh Ebrahimi Meimand, Omar Abdelsadek Abdou El Meligy, Iman El Sayed, Frank J Elgar, Holly E Erskine, Adeniyi Francis Fagbamigbe, Pawan Sirwan Faris, Andre Faro, Alize J Ferrari, Irina Filip, Florian Fischer, Aravind P Gandhi, Balasankar Ganesan, Miglas Welay Gebregergis, Mesfin Gebrehiwot, Bardiya Ghaderi Yazdi, Mohammad-Reza Ghasemi, Afsaneh Ghasemzadeh, Sasidhar Gunturu, Vivek Kumar Gupta, Sobia Ahsan Halim, Brian J Hall, Ahmed I Hasaballah, Simon I Hay, Bartosz Helfer, Md Mahbub Hossain, Bing-Fang Hwang, Umar Idris Ibrahim, Mehran Ilaghi, Md. Rabiul Islam, Sheikh Mohammed Shariful Islam, Mahalaxmi Iyer, Khushleen Jaggi, Haitham Jahrami, Elham Jamshidi, Ali Khaleghi, Abdul Aziz Khan, Mohammad Jobair Khan, Feriha Fatima Khidri, Kwanghyun Kim, Hyun Yong Koh, Manasi Kumar, Iván Landires, Long Khanh Dao Le, Seung Won Lee, Zhihui Li, Stephen S Lim, Jose Martinez-Raga, Roy Rillera Marzo, Indu Liz Matthew, Andrea Maugeri, Tomislav Mestrovic, Paul A Miller, Philip B Mitchell, Salahuddin Mohammed, Ali H Mokdad, Fateme Montazeri, Faraz Mughal, Christopher J L Murray, Woojae Myung, Javaid Nauman, Charles Richard James Newton, Huong Lan Thi Nguyen, Chisom Adaobi Nri-Ezedi, Vincent Ebuka Nwatah, Adeolu Olufunso Oladunjoye, Isaac Iyinoluwa Olufadewa, Michal Ordak, Nikita Otstavnov, Romil R Parikh, Seoyeon Park, Maja Pasovic, Jay Patel, Marcos Pereira, Maria Odete Pereira, Michael R Phillips, Guilherme V Polanczyk, Mohammad Pourfridoni, Jagadeesh Puvvula, Amir Radfar, Fakher Rahim, Mosiur Rahman, Muhammad Aziz Rahman, Amir Masoud Rahmani, Masoud Rahmati, Zubair Ahmed Ratan, Taeho Gregory Rhee, Priyanka Roy, Basema Ahmad Saddik, Amene Saghazadeh, Joseph W Sakshaug, Sana Salehi, Vijaya Paul Samuel, Senthilkumar Sankararaman, Damian F Santomauro, Maheswar Satpathy, David C Schwebel, James G Scott, Mario Šekerija, Arman Shafiee, Saeed Shahabi, Muhammad Aaqib Shamim, João Pedro Silva, Yonatan Solomon, Lourdes Bernadette C Sumpaico-Tanchanco, Chandan Kumar Swain, Rafael Tabarés-Seisdedos, Mohamad-Hani Temsah, Samuel Joseph Tromans, Lilian Tzivian, Maria Fernanda Vinueza Veloz, Theo Vos, Mandaras Tariku Walde, Muhammad Waqas, Harvey A Whiteford, Nuwan Darshana Wickramasinghe, Renjulal Yesodharan, Dong Keon Yon, Yoosik Youm, Burhan Abdullah Zaman, Youjie Zeng, and Magdalena Zielińska.

Drafting the work or revising it critically for important intellectual content

Isaac Yeboah Addo, Qorinah Estiningtyas Sakilah Adnani, Bright Opoku Ahinkorah, Ayman Ahmed, Fadwa Naji Alhalaiqa, Mohammed Usman Ali, Sami Almustanyir, Yasser Sami Abdel Dayem Amer, Sohrab Amiri, Dhanalakshmi Angappan, Catherine M Antony, Amadou Barrow, Akshaya Srikanth Bhagavathula, Sonu Bhaskar, Jasvinder Singh Singh Bhatti, Srinivasa Rao Bolla, Gabrielle Britton, Traolach Brugha, Joao Mauricio Castaldelli-Maia, Ferrán Catalá-López, Arthur Caye, Vijay Kumar Chattu, Yuen Yu Chong, Liliana G Ciobanu, Samuele Cortese, Natalia Cruz-Martins, Amira Hamed Darwish, Alejandro de la Torre-Luque, Daniel Diaz, Delaney D Ding, Arkadiusz Marian Dziedzic, Sepideh Ebrahimi Meimand, Omar Abdelsadek Abdou El Meligy, Iman El Sayed, Frank J Elgar, Holly E Erskine, Adeniyi Francis Fagbamigbe, Andre Faro, Alize J Ferrari, Nuno Ferreira, Irina Filip, Florian Fischer, Balasankar Ganesan, Miglas Welay Gebregergis, Bardiya Ghaderi Yazdi, Mohammad-Reza Ghasemi, Afsaneh Ghasemzadeh, Sasidhar Gunturu, Veer Bala Gupta, Sobia Ahsan Halim, Brian J Hall, Fulei Han, Josep Maria Haro, Ahmed I Hasaballah, Simon I Hay, Darren Hedley, Bartosz Helfer, Md Mahbub Hossain, Umar Idris Ibrahim, Mehran Ilaghi, Md. Rabiul Islam, Sheikh Mohammed Shariful Islam, Mahalaxmi Iyer, Khushleen Jaggi, Haitham Jahrami, Feriha

Fatima Khidri, Kwanghyun Kim, Hyun Yong Koh, Iván Landires, Zhihui Li, Jose Martinez-Raga, Roy Rillera Marzo, Indu Liz Matthew, Andrea Maugeri, Tomislav Mestrovic, Paul A Miller, Salahuddin Mohammed, Ali H Mokdad, Lorenzo Monasta, Fateme Montazeri, Matías Mrejen, Faraz Mughal, Javaid Nauman, Charles Richard James Newton, Huong Lan Thi Nguyen, Chisom Adaobi Nri-Ezedi, Vincent Ebuka Nwatah, Michal Ordak, Nikita Otstavnov, Raul Felipe Palma-Alvarez, Romil R Parikh, Jay Patel, Marcos Pereira, Michael R Phillips, Guilherme V Polanczyk, Mohammad Pourfridoni, Jagadeesh Puvvula, Amir Radfar, Fakher Rahim, Amir Masoud Rahmani, Zubair Ahmed Ratan, Luca Ronfani, Basema Ahmad Saddik, Vijaya Paul Samuel, Damian F Santomauro, Aswini Saravanan, Maheswar Satpathy, David C Schwebel, James G Scott, Mario Šekerija, Arman Shafiee, Saeed Shahabi, Muhammad Aaqib Shamim, João Pedro Silva, Yonatan Solomon, Chandan Kumar Swain, Mohamad-Hani Temsah, Samuel Joseph Tromans, Lilian Tzivian, Ravi Prasad Varma, Maria Fernanda Vinueza Veloz, Theo Vos, Mandaras Tariku Walde, Harvey A Whiteford, Nuwan Darshana Wickramasinghe, Renjulal Yesodharan, Dong Keon Yon, Yoosik Youm, Burhan Abdullah Zaman, and Magdalena Zielińska.

Managing the estimation or publications process

Alize J Ferrari, Hailey Hagins, Simon I Hay, Ali H Mokdad, Christopher J L Murray, Maja Pasovic, Damian F Santomauro, Theo Vos, and Harvey A Whiteford.
