# Supplementary material for: The global epidemiology and health burden of the autism spectrum: findings from the Global Burden of Disease Study 2021
Source: Lancet Psychiatry. 2025 Feb;12(2):111–21. doi: 10.1016/S2215-0366(24)00363-8 (PMC11750762; doi:10.1016/S2215-0366(24)00363-8)
Supplement: Supplementary appendix 2 [file mmc2.pdf]

THE LANCET
Psychiatry

Supplementary appendix 2

This appendix formed part of the original submission and has been peer reviewed. We post it as supplied by the authors.

Supplement to: Global Burden of Disease Study 2021 Autism Spectrum Collaborators. The global epidemiology and health burden of the autism spectrum: findings from the Global Burden of Disease Study 2021. *Lancet Psychiatry* 2024; published online Dec 19. https://doi.org/10.1016/S2215-0366(24)00363-8.

Contents

Section 1. Supplementary methods 2

Section 1.1. Adjustments to input data for DisMod-MR 2.1 2

Section 1.2. Prior settings in the estimation of the epidemiology of ASD in DisMod-MR 2.1 2

Section 1.3. Estimation of the proportion of autistic persons with each level of intellectual disability 3

Section 1.4. Estimation of the sequela-specific disability weights 4

Section 1.5. Comorbidity adjustment to years lived with disability 4

Section 2. Figures and tables 5

Figure S1: Overview of the estimation of prevalence and DALYs for the autism spectrum 5

Figure S2: Preferred Reporting Items for Systematic Reviews and Meta-Analyses (PRISMA) 2009 flow diagram for the GBD 2017 initial systematic-review 6

Figure S3: Preferred Reporting Items for Systematic Reviews and Meta-Analyses (PRISMA) 2020 flow diagram for the GBD 2021 systematic-review update 7

Figure S4: Hierarchy of meta-analyses conducted to estimate the proportion of autistic persons with each level of ID 8

Figure S5: Forest plot of the meta-analysis of the proportion of autistic persons that present with any ID 9

Figure S6: Forest plot of the meta-analysis of the proportion of autistic persons without ID that present with borderline ID 9

Figure S7: Forest plot of the meta-analysis of the proportion of autistic persons with ID that present with mild ID 10

Figure S8: Forest plot of the meta-analysis of the proportion of autistic persons with moderate, severe, or profound ID that present with moderate ID 10

Figure S9: Forest plot of the meta-analysis of the proportion of autistic persons with severe or profound ID that present with severe ID 11

Figure S10: Forest plot of the meta-analysis of the proportion of autistic persons that meet criteria for DSM-IV autistic disorder 11

Table S1: Guidelines for Accurate and Transparent Health Estimates Reporting (GATHER) checklist 12

Table S2: Search strings used in electronic databases 14

Table S3: Preferred Reporting Items for Systematic Reviews and Meta-Analyses (PRISMA) checklist 15

Table S4: Lay descriptions and disability weights for the health states informing autism spectrum sequelae in the GBD 2021 Study 18

Table S5: Age-standardised prevalence of ASD per 100 000 persons by location and sex, 2021 19

Table S6: Age-standardised prevalence of ASD sequelae per 100 000 persons by location, 2021 30

Table S7: Age-standardised DALY rates per 100 000 persons attributable to ASD by location and sex, 2021 50

Section 3. References 61

Section 1. Supplementary methods

Section 1.1. Adjustments to input data for DisMod-MR 2.1

Two adjustments were applied to estimates prior to analysis in DisMod-MR 2.1. First, data that aggregated across males and females within the study sample were split into sex-specific estimates. For data sources reporting age-specific and separately, sex-specific estimates (but not age-sex-specific estimates), the age-specific estimates were adjusted using the within-study sex-ratio. Data sources not reporting sex-specific estimates were adjusted using the pooled within-study sex-ratio across all respective sex-specific estimates estimated via meta-regression—Bayesian, regularised, and trimmed (MR-BRT).[1] Second, known sources of bias in the input prevalence data were adjusted for using MR-BRT. Reference epidemiological data for ASD were estimates that represented autistic persons from general population surveys with additional case-finding or population screening. To maximise data-inclusion, estimates representing DSM-IV autistic disorder / ICD-10 childhood autism or sourced from general population surveys without additional case-finding were also included as non-reference data and adjusted for prior to analysis in DisMod-MR 2.1. Within the ASD epidemiological dataset, within-study estimates were paired by age, sex, location, and year, between the reference and alternative estimates. The difference in logit prevalence between these estimates were then used as inputs in a network meta-analysis conducted via meta-regression—Bayesian, regularised, and trimmed (MR-BRT).[1] This analysis produced pooled log-odds between the reference estimates and alternative estimates that were used to adjust all alternative estimates prior to analysis. To reflect the added uncertainty around alternative estimates, the uncertainty intervals (UIs) for the adjustment factors incorporated gamma from the MR-BRT analysis, which represented the between study standard deviation across all the input data. The pooled log-odds between estimates of the autism sub-disorder and ASD was 0.44 (95% UI: 0.39 – 0.50) whereas the pooled log-odds between estimates from general population surveys without additional case-finding and reference sampling methodology was 0.70 (95% UI: 0.61 – 0.82).

Section 1.2. Settings in the estimation of the epidemiology of ASD in DisMod-MR 2.1

GBD 2021 followed a cause hierarchy with four levels. Level 0 represented the aggregate of all causes within GBD 2021. Level 1 included other COVID-19 pandemic-related outcomes and aggregated groups of communicable, maternal, neonatal and nutritional diseases; non-communicable diseases; and injuries respectively. Level 2 disaggregated level 1 causes into 22 smaller groupings (including the aggregate of all mental disorders). Level 3 disaggregated Level 2 causes into 175 diseases and injuries (including ASD). Level 4 further disaggregated 43 level 3 causes. Several prior settings were used to guide the DisMod-MR 2.1 estimation of prevalence based on the available data and feedback from experts in the field. Autistic persons were autistic across the lifespan (incidence at birth and no remission) and a maximum excess mortality rate of 0.3 was used to align with the upper confidence interval of the highest reported excess mortality estimate in the dataset. We allowed a time window of 15 years due to limited data across time, which meant estimates would inform prevalence 15 years before and after when the estimate was collected. The Healthcare Access Quality Index (HAQI) was placed as a location-level covariate on excess mortality. The HAQI is informed by the mortality rate of 32 causes of death which are typically prevented in the presence of effective care.[2]

Section 1.3. Estimation of the proportion of autistic persons with each level of intellectual disability

From the systematic review of epidemiological estimates, 19 studies were sourced that used the reference sampling methodology and reported the proportion of autistic persons by intellectual disability (ID). However, studies differed in the granularity of reporting with most studies only reporting the proportion of autistic persons that presented with any ID (IQ < 70). To maximise data-inclusion, five meta-analyses were conducted in R using the metafor package[3,4] to estimate the proportion of autistic persons living with each level of ID. Figure S4 illustrates the hierarchy of meta-analyses conducted and the number of studies used for each analysis. Each meta-analysis split the autistic population into finer groups of ID. For example meta-analysis #1 estimated the proportion of those with any ID out of all cases, and then meta-analysis #3 estimated the proportion of those with mild ID out of those with any ID.

No studies reported enough data to separate autistic persons meeting criteria for ASD by severe and profound ID. However there were six studies that looked at IQ by ASD sub-disorder, and together they suggested that the vast majority of cases with severe and profound ID would meet criteria for the autism sub-disorder of ASD.[5-10] Additionally there were five studies that provided enough data to separate persons meeting criteria for the autism sub-disorder by severe and profound ID. We therefore decided to conduct a meta-analysis on these five studies to inform this split.

Overall the pooled proportion of autistic persons that present with ID was 0.36 (95%UI: 0.30-0.42). Among autistic persons that do not present with ID, the pooled estimate of the proportion that present with borderline ID was 0.31 (95%UI: 0.26-0.36). Among those that do present with ID, the pooled estimate of the proportion presenting with mild ID was 0.42 (95%UI: 0.33-0.51). The pooled estimate of the proportion autistic persons presenting with moderate ID among those presenting with moderate, severe, or profound ID was 0.67 (95%UI: 0.53-0.78). Lastly the pooled proportion of autistic persons with severe or profound ID that present with profound ID was 0.19 (95%UI: 0.10-0.33). The forest plots of the meta-analyses are presented in Figures S5 to S9.

Because the denominator of meta-analyses #2 to #5 were subsamples of all autistic persons, the pooled proportions from these analyses had to be adjusted to represent the proportion of all autistic persons. We pulled 1000 draws (samples) from the logit distribution of proportion estimates from each meta-analysis, and adjusted the proportions at the draw level so that they represent the proportion of all autistic persons using the following formulas:

$$P_{No\ ID} = \left(1 - P_{Any\ ID}\right) \times (1 - S_{Borderline\ ID})$$

$$P_{Borderline\ ID} = \left(1 - P_{Any\ ID}\right) \times S_{Borderline\ ID}$$

$$P_{Mild\ ID} = P_{Any\ ID} \times S_{Mild\ ID}$$

$$P_{Moderate\ ID} = (P_{Any\ ID} - P_{Mild\ ID}) \times S_{Moderate\ ID}$$

$$P_{Severe\ ID} = (P_{Any\ ID} - P_{Mild\ ID} - P_{Moderate\ ID}) \times (1 - S_{Profound\ ID})$$

$$P_{Profound\ ID} = (P_{Any\ ID} - P_{Mild\ ID} - P_{Moderate\ ID}) \times S_{Profound\ ID}$$

Where *P* represents the proportion of all autistic persons, and *S* represents the proportion of the subsample from the meta-analysis. The 95% uncertainty intervals (UIs) were the 25th and 975th ranked values across the adjusted draws. This process produced severity proportions to split prevalence estimates into sequela-specific prevalence by age, sex, location, and year.

Section 1.4. Estimation of the sequela-specific disability weights

The disability weights of ASD in GBD 2021 represented the disability weight of ASD across six levels of ID, however, the disability weights surveys only provided disability weights for the health states *autism* and *Asperger's syndrome and other autism spectrum disorders* and *borderline*, *mild*, *moderate*, *severe*, and *profound intellectual disability* separatel. To estimate disability weights for ASD and each level of ID, we first compiled the disability weights for relevant health states (see Table S4). We then estimated a pooled overall disability weight for ASD from the *Autism* and *Asperger's syndrome and other ASD* disability weights. The systematic review sourced 19 studies using the reference sampling methodology that reported the proportion of autistic persons that met criteria for the autism sub-disorder. We conducted a meta-analysis in R using the metafor package[3,4] on these proportions to estimate a pooled proportion. The pooled proportion of ASD cases estimated to meet criteria for the autism sub-disorder was 0.41 (95%UI: 0.36-0.47, see Figure S10 for forest plot). We pulled 1000 draws (samples) from the logit distribution of the pooled proportion of autistic persons that met criteria for the autism sub-disorder. We then pulled 1000 draws from the distribution of disability weights for *Autism* and *Asperger's syndrome and other ASD,* and weighted the draws of the disability weights by the draws of the pooled proportion:

$$DW_{ASD} = DW_{Autism} \times P_{Autism} + \ DW_{Other\ ASD} \times (1 - P_{Autism})$$

Where *DW* is disability weight and P_{Autism} is the proportion of autistic persons estimated to meet criteria for the DSM-IV autistic disorder / ICD-10 childhood autism. Then the disability weight for ASD and each level of ID was calculated as:

$$DW_{ASD+ID} = 1 - (1 - DW_{ASD}) \times (1 - DW_{ID})$$

Section 1.5. Comorbidity adjustment to years lived with disability

Sequela-specific prevalence estimates across all causes of burden were multiplied by their respective disability weights to calculate YLDs. This process was conducted for each sequela separately; however, an individual may live with multiple sequelae from different conditions simultaneously. To adjust YLDs for comorbidity, the co-occurrence of sequelae was simulated within a population of 20 000 simulated individuals for every age, sex, location, and year. Simulants were exposed to the independent probability of each sequela based on the modelled prevalence of that sequela. The probability of a simulant having two or more sequelae was therefore the product of the prevalence of those sequelae. A disability weight was then assigned for each simulant based on the sequelae that simulant had. Simulated individuals with multiple sequelae were estimated to have a cumulative disability weight that was a multiplicative function of the disability weights of those sequelae, and the disability weight uniquely attributable to each sequela was adjusted accordingly. The YLD rate for each sequela was then calculated for each simulated population (i.e. each age, sex, location, and year). Total YLD count for each sequela was calculated as the YLD rate multiplied by the population. YLDs for ASD were then calculated as the sum of the YLDs for the ASD sequelae. This simulation was repeated 500 times, with the uncertainty of prevalence, severity, and disability weights propagated throughout the calculations.

Section 2. Figures and tables

Figure S1: Overview of the estimation of prevalence and DALYs for the autism spectrum

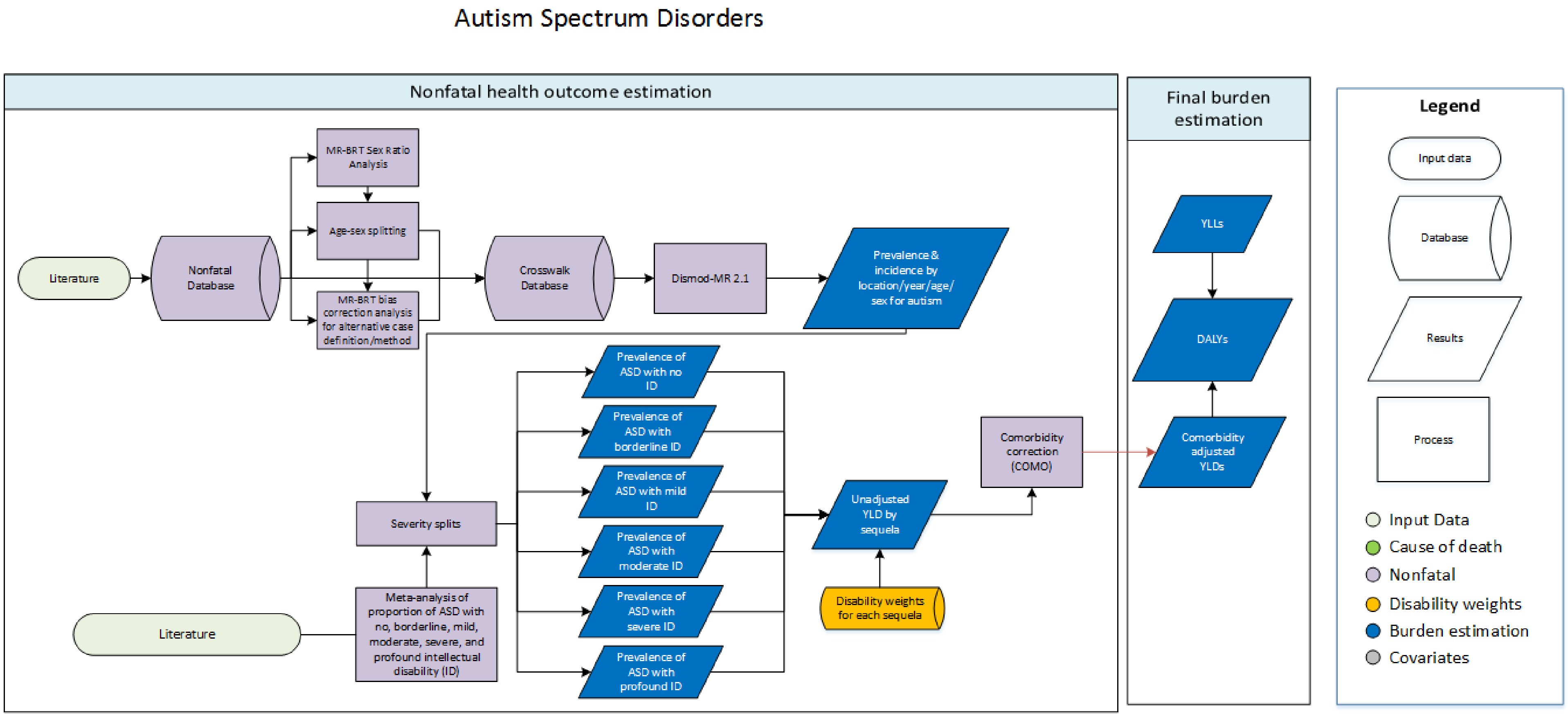

Figure S2: Preferred Reporting Items for Systematic Reviews and Meta-Analyses11 (PRISMA) 2009 flow diagram for the GBD 2017 initial systematic-review

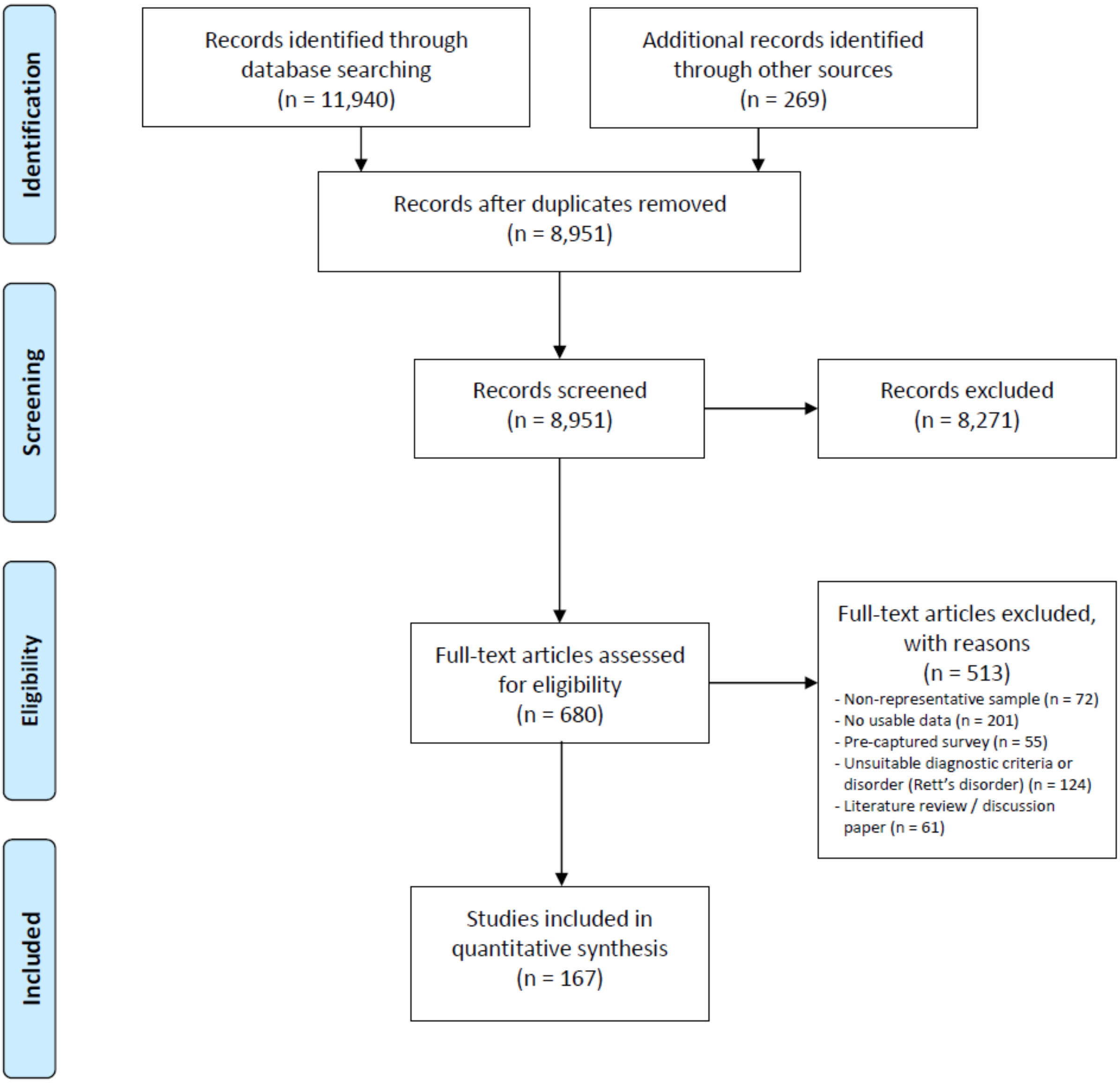

Note: The number of studies reported as "included in the previous version of review" for the GBD 2021 systematic review update (Figure S4) differs from the number of studies reported as included from the initial GBD 2017 systematic review (Figure S3) for several reasons. First, additional sources were provided by GBD collaborators for GBD 2019. Second, estimates using passive case finding (e.g., registry or administrative prevalence estimates) were excluded for GBD 2021 based on expert feedback. Third, new estimates may represent more granular estimates (e.g., by age or sex) of pre-captured studies and therefore new studies may replace (rather than add to) pre-captured studies.

Figure S3: Preferred Reporting Items for Systematic Reviews and Meta-Analyses[12] (PRISMA) 2020 flow diagram for the GBD 2021 systematic-review update

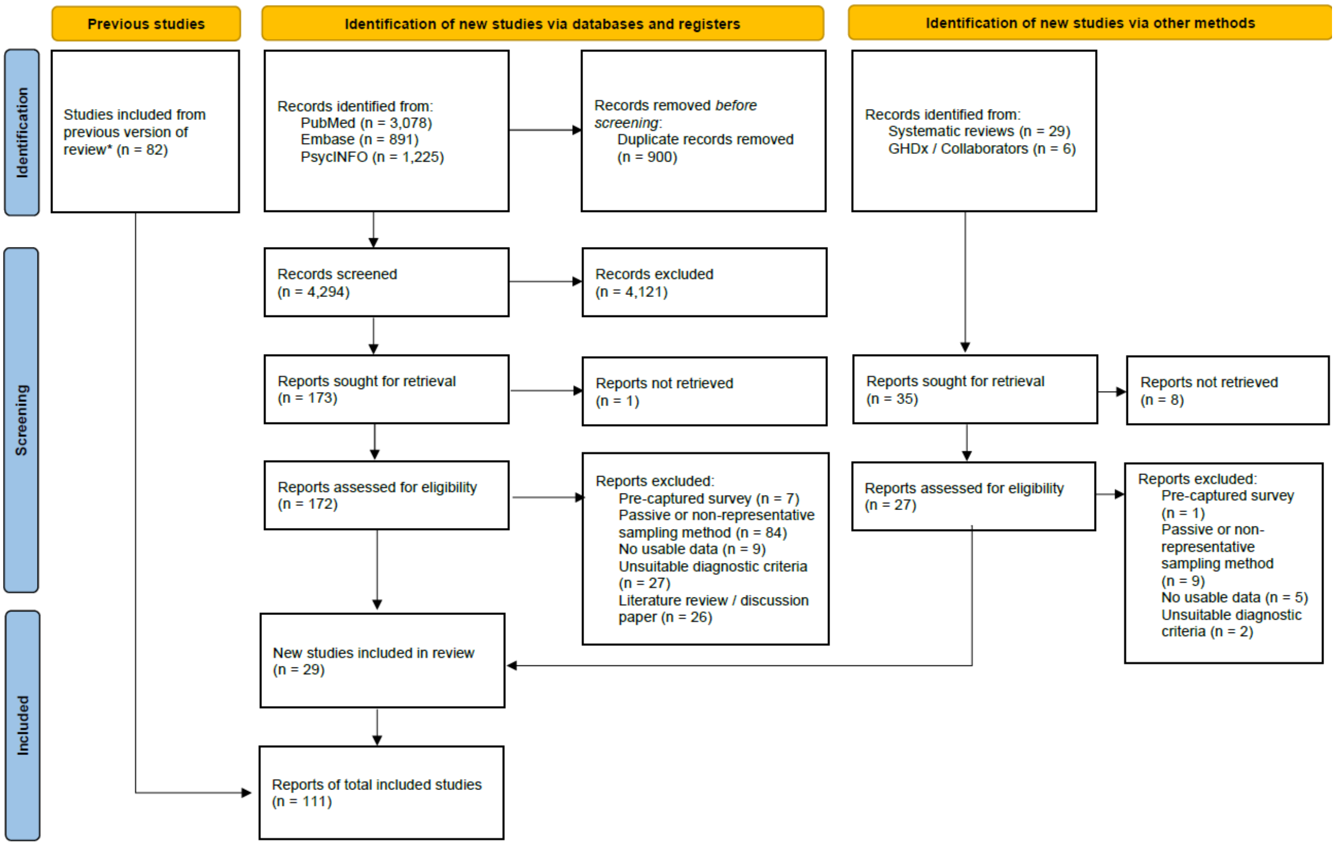

The number of studies reported as “included in the previous version of review” for the GBD 2021 systematic review update (Figure S4) differs from the number of studies reported as included from the GBD 2017 initial systematic review (Figure S3) for several reasons. First, additional sources were provided by GBD collaborators for GBD 2019. Second, estimates using passive case finding (e.g., registry or administrative prevalence estimates) were excluded for GBD 2021 based on expert feedback. Third, new estimates may represent more granular estimates (e.g., by age or sex) of pre-captured studies and therefore new studies may replace (rather than add to) pre-captured studies.

Figure S4: Hierarchy of meta-analyses conducted to estimate the proportion of autistic persons with each level of ID

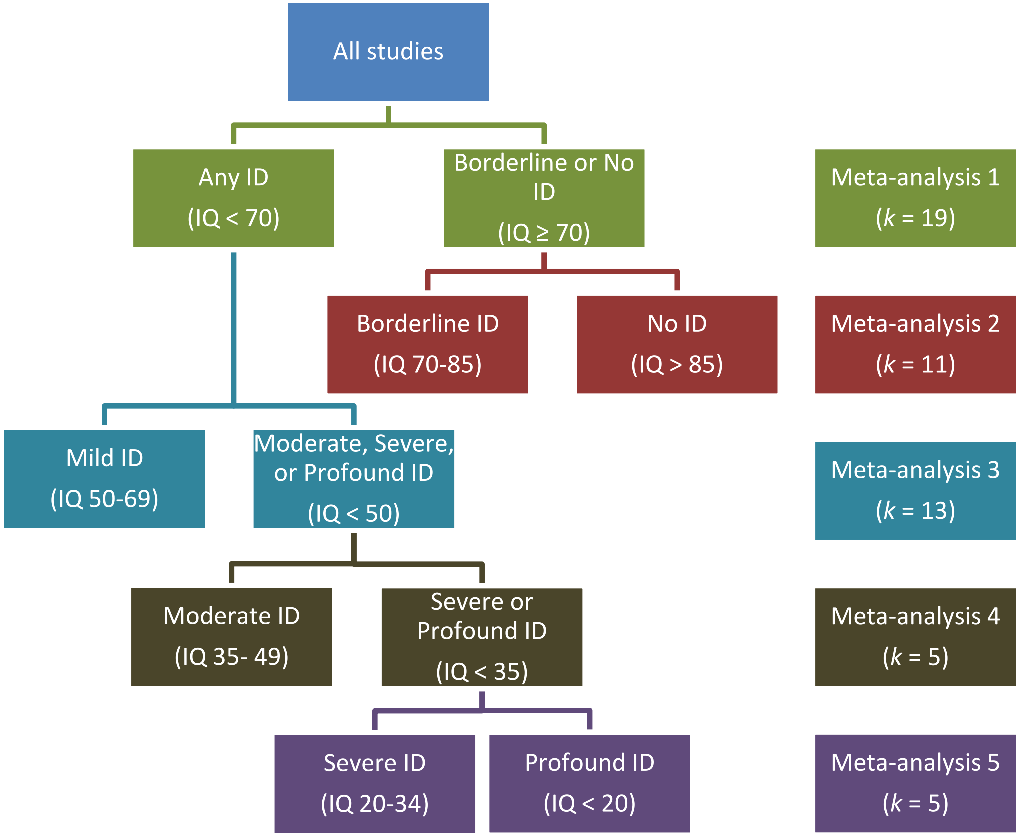

Note: *k* = number of studies used in meta-analysis. ID = Intellectual Disability. IQ = Intelligence Quotient.

Figure S5: Forest plot of the meta-analysis of the proportion of autistic persons that present with any ID

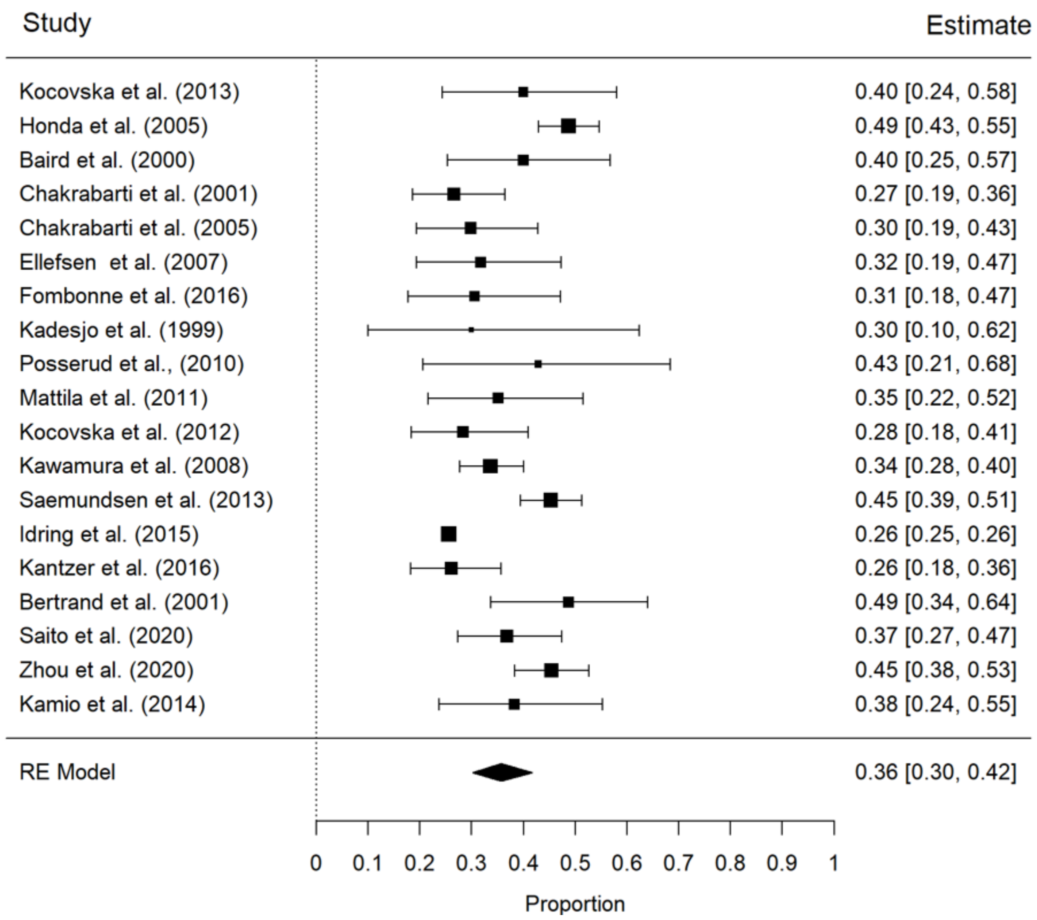

Note: Citations[5-10,13-25]

Figure S6: Forest plot of the meta-analysis of the proportion of autistic persons without ID that present with borderline ID

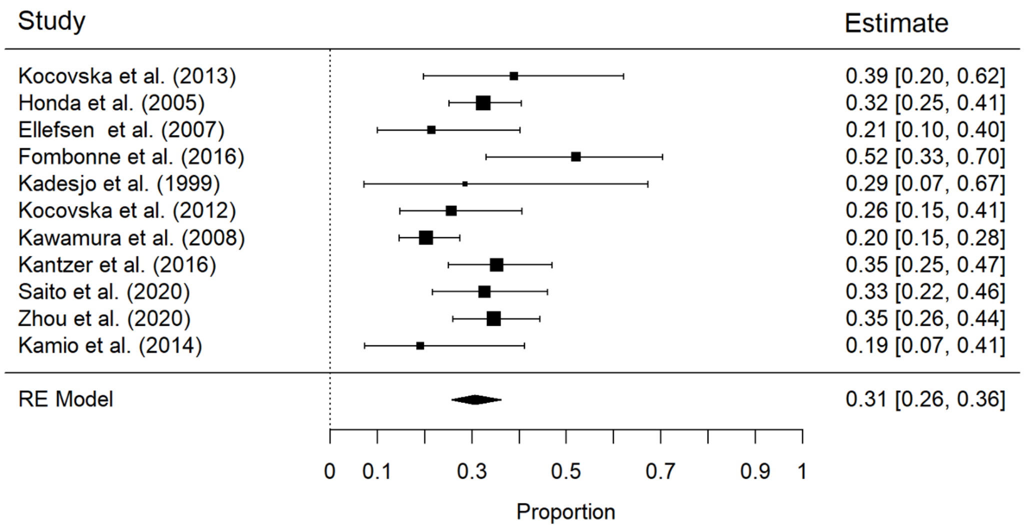

Note: Citations[7-9,13,14,16,18,21,23-25]

Figure S7: Forest plot of the meta-analysis of the proportion of autistic persons with ID that present with mild ID

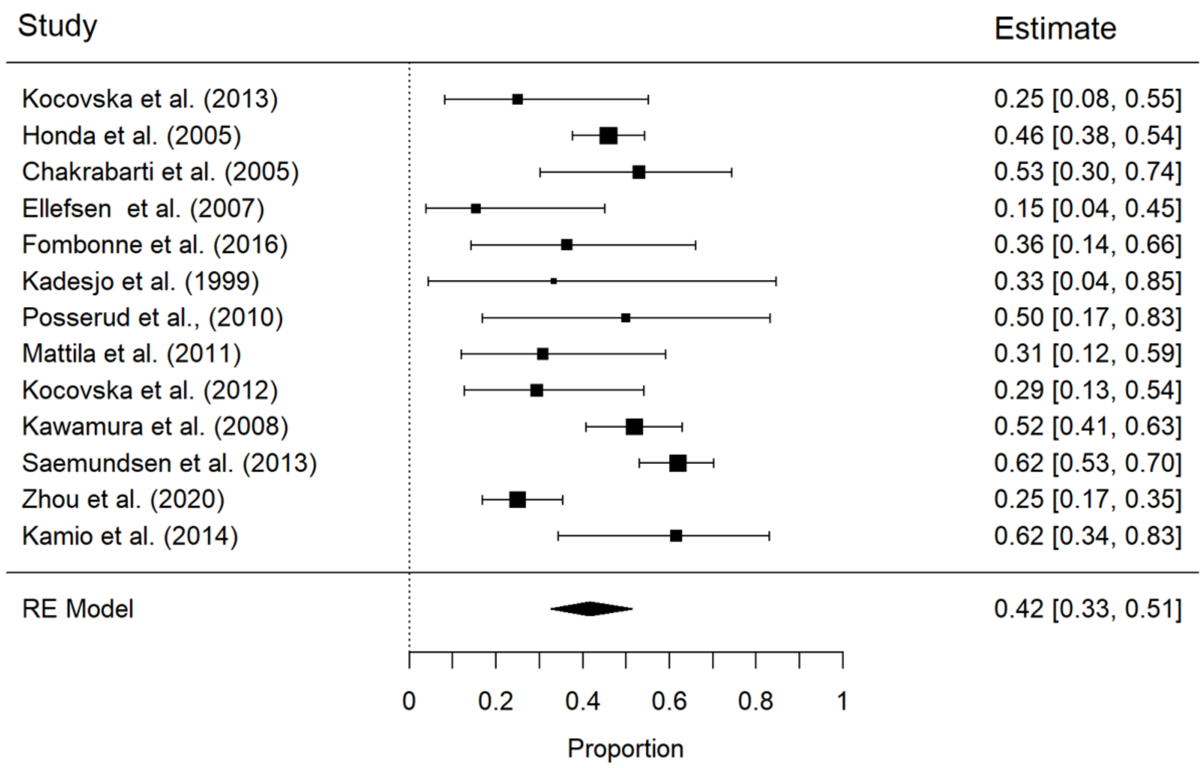

Note: Citations[6-10,13,14,16-19,24,25]

Figure S8: Forest plot of the meta-analysis of the proportion of autistic persons with moderate, severe, or profound ID that present with moderate ID

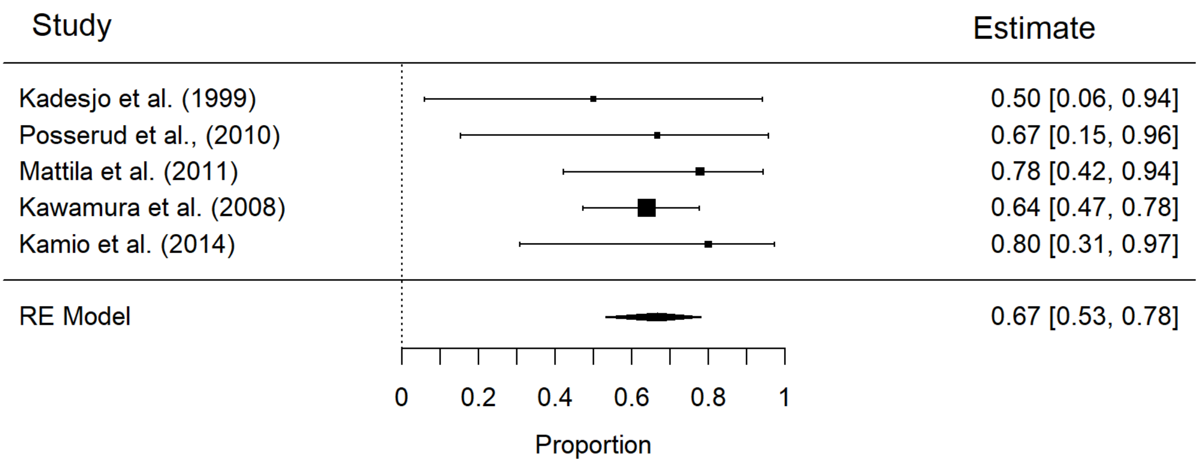

Note: Citations[7,10,17,18,25]

Figure S9: Forest plot of the meta-analysis of the proportion of autistic persons with severe or profound ID that present with severe ID

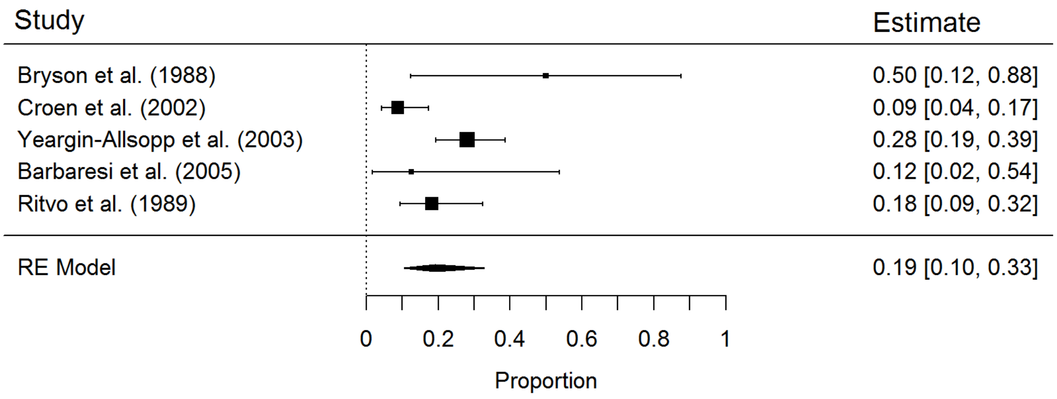

Note: Citations[26-30]

Figure S10: Forest plot of the meta-analysis of the proportion of autistic persons that meet criteria for DSM-IV autistic disorder

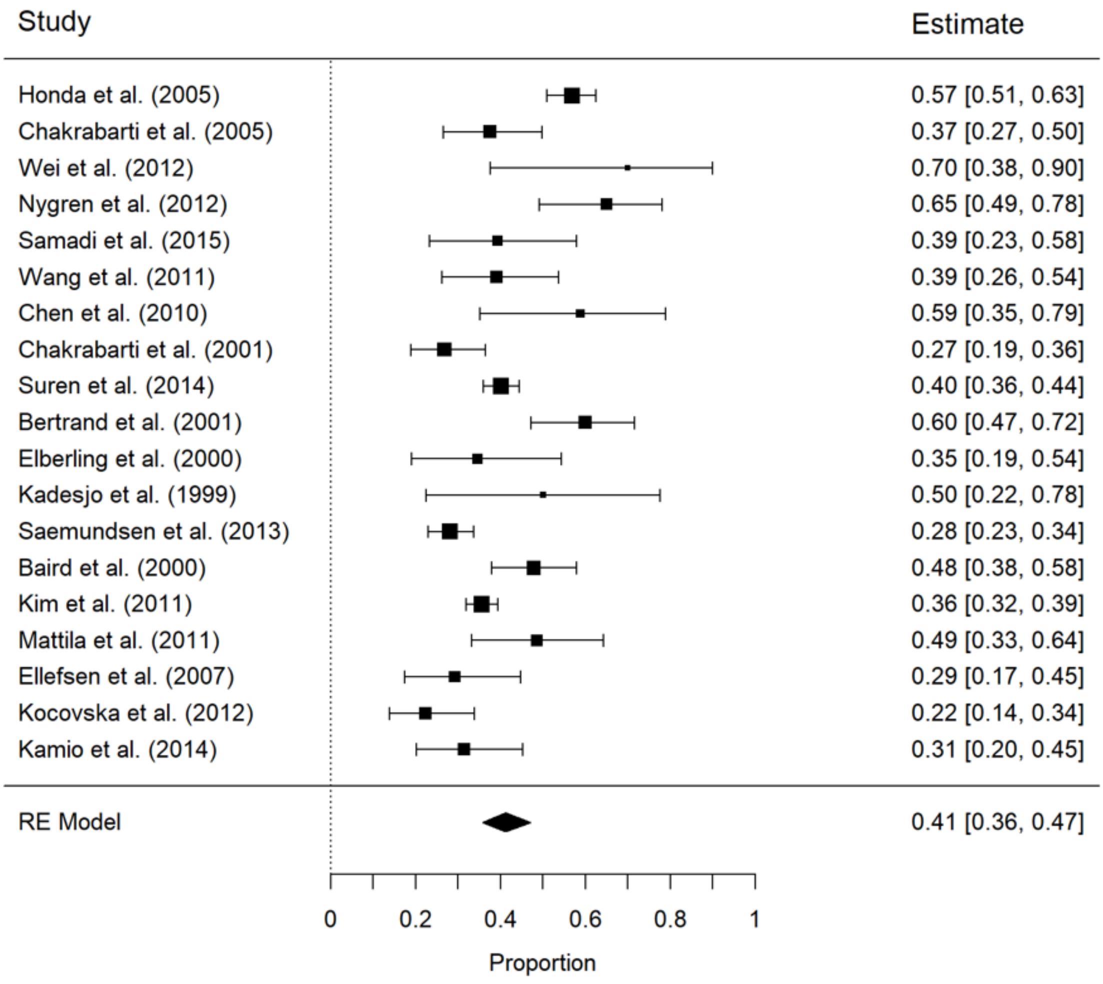

Note: Citations[5-10,14,15,19,22,25,31-38]

Table S1: Guidelines for Accurate and Transparent Health Estimates Reporting (GATHER) checklist

Item #	Checklist item	Location
**Objectives and funding**		
1	Define the indicator(s), populations (including age, sex, and geographic entities), and time period(s) for which estimates were made.	Method (first paragraph)
2	List the funding sources for the work.	Acknowledgements
**Data Inputs**		
For all data inputs from multiple sources that are synthesized as part of the study:		
3	Describe how the data were identified and how the data were accessed.	Method: *Search Strategy*
4	Specify the inclusion and exclusion criteria. Identify all ad-hoc exclusions.	Method: *Inclusion criteria and data extraction*
5	Provide information on all included data sources and their main characteristics. For each data source used, report reference information or contact name/institution, population represented, data collection method, year(s) of data collection, sex and age range, diagnostic criteria or measurement method, and sample size, as relevant.	Results<br>*Reference to GBD 2021 Data Input Sources Tool.*
6	Identify and describe any categories of input data that have potentially important biases (e.g., based on characteristics listed in item 5).	Method: *Prevalence estimation*<br>Appendix: *Section 1.1.*
For data inputs that contribute to the analysis but were not synthesized as part of the study:		
7	Describe and give sources for any other data inputs.	N/A
For all data inputs:		
8	Provide all data inputs in a file format from which data can be efficiently extracted (e.g., a spreadsheet rather than a PDF), including all relevant meta-data listed in item 5. For any data inputs that cannot be shared because of ethical or legal reasons, such as third-party ownership, provide a contact name or the name of the institution that retains the right to the data.	*Reference to GBD 2021 Data Input Sources Tool which provides spreadsheet of input data.*
**Data analysis**		
9	Provide a conceptual overview of the data analysis method. A diagram may be helpful.	Figure S1

10	Provide a detailed description of all steps of the analysis, including mathematical formulae. This description should cover, as relevant, data cleaning, data pre-processing, data adjustments and weighting of data sources, and mathematical or statistical model(s).	Method: *Estimation of epidemiology*<br>Method: *Estimation of severity distribution*<br>Method: *Estimation of disability weights*<br>Method: *Estimation of disability-adjusted life years*<br>Appendix: *Section 1.1.*<br>Appendix: *Section 1.2.*<br>Appendix: *Section 1.3.*<br>Appendix: *Section 1.4.*<br>Appendix: *Section 1.5.*
11	Describe how candidate models were evaluated and how the final model(s) were selected.	Method: *Estimation of epidemiology*<br>Method: *Estimation of severity distribution*<br>Method: *Estimation of disability weights*<br>Method: *Estimation of disability-adjusted life years*<br>Appendix: *Section 1.1.*<br>Appendix: *Section 1.2.*<br>Appendix: *Section 1.3.*<br>Appendix: *Section 1.4.*<br>Appendix: *Section 1.5.*
12	Provide the results of an evaluation of model performance, if done, as well as the results of any relevant sensitivity analysis.	N/A
13	Describe methods for calculating uncertainty of the estimates. State which sources of uncertainty were, and were not, accounted for in the uncertainty analysis.	Method (first paragraph)
14	State how analytic or statistical source code used to generate estimates can be accessed.	*Reference to GBD 2021 statistical code page*
**Results and Discussion**		
15	Provide published estimates in a file format from which data can be efficiently extracted.	*Reference to GBD 2021 results tool*
16	Report a quantitative measure of the uncertainty of the estimates (e.g. uncertainty intervals).	*All estimates are provided with uncertainty intervals*
17	Interpret results in light of existing evidence. If updating a previous set of estimates, describe the reasons for changes in estimates.	Discussion
18	Discuss limitations of the estimates. Include a discussion of any modelling assumptions or data limitations that affect interpretation of the estimates.	Discussion

Table S2: Search strings used in electronic databases

Database	Search string
PubMed	((((((autis*[Title/Abstract]) OR asperger*[Title/Abstract]) OR ((pervasive[Title/Abstract]) AND disorder*[Title/Abstract]))) OR "Child Development Disorders, Pervasive"[Mesh])) AND ((((((((((((prevalen*[Title/Abstract]) OR mortalit*[Title/Abstract]) OR death*[Title/Abstract]) OR inciden*[Title/Abstract]) OR remission[Title/Abstract]) OR duration[Title/Abstract]) OR remit*[Title/Abstract]) OR epidemiolog*[Title/Abstract]) OR "Prevalence"[Mesh]) OR "Mortality"[Mesh]) OR "Incidence"[Mesh]) OR "Epidemiology"[Mesh:NoExp]) OR "Morbidity"[Mesh:NoExp])
Embase	(autis*:ab,ti OR asperger*:ab,ti OR (pervasive:ab,ti AND disorder*:ab,ti) OR 'autism'/exp) AND 'human'/de AND (prevalen*:ab,ti OR mortalit*:ab,ti OR death*:ab,ti OR inciden*:ab,ti OR remission:ab,ti OR duration:ab,ti OR remit*:ab,ti OR epidemiolog*:ab,ti OR 'prevalence'/exp OR 'epidemiology'/exp OR 'remission'/exp) AND [embase]/lim NOT [medline]/lim AND ('article'/it OR 'article in press'/it)
PsycINFO	(TI autis* OR AB autis* OR TI asperger* OR AB asperger* OR ((TI pervasive OR AB pervasive) AND (TI disorder* OR AB disorder*)) OR DE "Autism Spectrum Disorders") AND (TI prevalen* OR AB prevalen* OR TI mortalit* OR AB mortalit* OR TI death* OR AB death* OR TI inciden* OR AB inciden* OR TI remission OR AB remission OR TI duration OR AB duration OR TI remit* OR AB remit* OR TI epidemiolog* OR AB epidemiolog* OR DE "Mortality Rate" OR DE "Epidemiology" OR DE "Morbidity" OR DE "Remission (Disorders)")

Table S3: Preferred Reporting Items for Systematic Reviews and Meta-Analyses (PRISMA) checklist

Section and Topic	Item #	Checklist item	Location where item is reported
**TITLE**			
Title	1	Identify the report as a systematic review.	*The title instead reflects the focus of this study, which was to report the results from the Global Burden of Disease Study 2021.*
**ABSTRACT**			
Abstract	2	See the PRISMA 2020 for Abstracts checklist.	*The abstract instead reflects the focus of this study, which was to report the results from the Global Burden of Disease Study 2021.*
**INTRODUCTION**			
Rationale	3	Describe the rationale for the review in the context of existing knowledge.	Introduction
Objectives	4	Provide an explicit statement of the objective(s) or question(s) the review addresses.	Introduction
**METHODS**			
Eligibility criteria	5	Specify the inclusion and exclusion criteria for the review and how studies were grouped for the syntheses.	Method: *Inclusion criteria and data extraction*
Information sources	6	Specify all databases, registers, websites, organisations, reference lists and other sources searched or consulted to identify studies. Specify the date when each source was last searched or consulted.	Method: *Search Strategy*
Search strategy	7	Present the full search strategies for all databases, registers and websites, including any filters and limits used.	Method: *Search Strategy*<br>Table S2
Selection process	8	Specify the methods used to decide whether a study met the inclusion criteria of the review, including how many reviewers screened each record and each report retrieved, whether they worked independently, and if applicable, details of automation tools used in the process.	Method: *Search Strategy*
Data collection process	9	Specify the methods used to collect data from reports, including how many reviewers collected data from each report, whether they worked independently, any processes for obtaining or confirming data from study investigators, and if applicable, details of automation tools used in the process.	Method: *Search Strategy*<br>Method: *Inclusion criteria and data extraction*
Data items	10a	List and define all outcomes for which data were sought. Specify whether all results that were compatible with each outcome domain in each study were sought (e.g. for all measures, time points, analyses), and if not, the methods used to decide which results to collect.	Method: *Inclusion criteria and data extraction*
	10b	List and define all other variables for which data were sought (e.g. participant and intervention characteristics, funding sources). Describe any assumptions made about any missing or unclear information.	Method: *Inclusion criteria and data extraction*
Study risk of bias assessment	11	Specify the methods used to assess risk of bias in the included studies, including details of the tool(s) used, how many reviewers assessed each study and whether they worked independently, and if applicable, details of automation tools used in the process.	Method: *Estimation of epidemiology*<br>Appendix: *Section 1.1.*
Effect measures	12	Specify for each outcome the effect measure(s) (e.g. risk ratio, mean difference) used in the synthesis or presentation of results.	Method: *Estimation of epidemiology*
Synthesis methods	13a	Describe the processes used to decide which studies were eligible for each synthesis (e.g. tabulating the study intervention characteristics and comparing	Method: *Inclusion criteria and data*

Section and Topic	Item #	Checklist item	Location where item is reported
		against the planned groups for each synthesis (item #5)).	*extraction*
	13b	Describe any methods required to prepare the data for presentation or synthesis, such as handling of missing summary statistics, or data conversions.	Method: *Estimation of epidemiology*<br>Appendix: *Section 1.1.*
	13c	Describe any methods used to tabulate or visually display results of individual studies and syntheses.	N/A
	13d	Describe any methods used to synthesize results and provide a rationale for the choice(s). If meta-analysis was performed, describe the model(s), method(s) to identify the presence and extent of statistical heterogeneity, and software package(s) used.	Method: *Estimation of epidemiology*
	13e	Describe any methods used to explore possible causes of heterogeneity among study results (e.g. subgroup analysis, meta-regression).	Method: *Estimation of epidemiology*<br>Appendix: *Section 1.1.*
	13f	Describe any sensitivity analyses conducted to assess robustness of the synthesized results.	N/A
Reporting bias assessment	14	Describe any methods used to assess risk of bias due to missing results in a synthesis (arising from reporting biases).	Method: *Inclusion criteria and data extraction*
Certainty assessment	15	Describe any methods used to assess certainty (or confidence) in the body of evidence for an outcome.	Method (first paragraph)
**RESULTS**			
Study selection	16a	Describe the results of the search and selection process, from the number of records identified in the search to the number of studies included in the review, ideally using a flow diagram.	Results<br>Figure S3
	16b	Cite studies that might appear to meet the inclusion criteria, but which were excluded, and explain why they were excluded.	N/A
Study characteristics	17	Cite each included study and present its characteristics.	*Reference to GBD 2021 Data Input Sources Tool which provides spreadsheet of input data.*
Risk of bias in studies	18	Present assessments of risk of bias for each included study.	Method: *Estimation of epidemiology*<br>Appendix: *Section 1.1.*<br>*Risk of methodological bias was considered within the framework of the bias corrections applied.*
Results of individual studies	19	For all outcomes, present, for each study: (a) summary statistics for each group (where appropriate) and (b) an effect estimate and its precision (e.g. confidence/credible interval), ideally using structured tables or plots.	*All estimates are provided with uncertainty intervals*
Results of syntheses	20a	For each synthesis, briefly summarise the characteristics and risk of bias among contributing studies.	Results<br>*Reference to GBD 2021 Data Input Sources Tool which provides spreadsheet of input data.*<br>Appendix: *Section 1.1.*<br>*Information on study methodology was extracted and assessed, and the purpose of the meta-regressions in* Appendix: *Section 1.1. to adjust*

Section and Topic	Item #	Checklist item	Location where item is reported
			estimates for bias prior to analysis.
	20b	Present results of all statistical syntheses conducted. If meta-analysis was done, present for each the summary estimate and its precision (e.g. confidence/credible interval) and measures of statistical heterogeneity. If comparing groups, describe the direction of the effect.	Results<br>Appendix: *Section 1.1.*<br>Appendix: *Section 1.3.*<br>Appendix: *Section 1.4.*<br>Appendix: *Section 1.5.*
	20c	Present results of all investigations of possible causes of heterogeneity among study results.	Results<br>Appendix: *Section 1.1.*
	20d	Present results of all sensitivity analyses conducted to assess the robustness of the synthesized results.	N/A
Reporting biases	21	Present assessments of risk of bias due to missing results (arising from reporting biases) for each synthesis assessed.	N/A
Certainty of evidence	22	Present assessments of certainty (or confidence) in the body of evidence for each outcome assessed.	*All estimates are provided with uncertainty intervals*
**DISCUSSION**			
Discussion	23a	Provide a general interpretation of the results in the context of other evidence.	Discussion
	23b	Discuss any limitations of the evidence included in the review.	Discussion
	23c	Discuss any limitations of the review processes used.	Discussion
	23d	Discuss implications of the results for practice, policy, and future research.	Discussion
**OTHER INFORMATION**			
Registration and protocol	24a	Provide registration information for the review, including register name and registration number, or state that the review was not registered.	PROSPERO registration: CRD42021257501
	24b	Indicate where the review protocol can be accessed, or state that a protocol was not prepared.	Protocol is available on PROSPERO
	24c	Describe and explain any amendments to information provided at registration or in the protocol.	Revisions to PROSPERO registration reflect name changes to the GBD iteration, team members, and funding sources
Support	25	Describe sources of financial or non-financial support for the review, and the role of the funders or sponsors in the review.	Acknowledgements
Competing interests	26	Declare any competing interests of review authors.	Declarations of interest
Availability of data, code and other materials	27	Report which of the following are publicly available and where they can be found: template data collection forms; data extracted from included studies; data used for all analyses; analytic code; any other materials used in the review.	Results<br>*Reference to GBD 2021 Data Input Sources Tool*

Table S4: Lay descriptions and disability weights for the health states informing autism spectrum sequelae in the GBD 2021 Study

Health state	Lay description	Disability weight (95% UI)
Autism	Has severe problems interacting with others and difficulty understanding simple questions or directions. The person has great difficulty with basic daily activities and becomes distressed by any change in routine.	0.262 (0.176–0.365)
Asperger's syndrome & other autism spectrum disorders	Has difficulty interacting with other people and is slow to understand or respond to questions. The person is often preoccupied with one thing and has some difficulty with basic daily activities.	0.104 (0.071–0.147)
Intellectual disability, borderline	Is slow in learning at school. As an adult, the person has some difficulty doing complex or unfamiliar tasks but otherwise functions independently.	0.011 (0.005–0.020)
Intellectual disability, mild	Has low intelligence and is slow in learning at school. As an adult, the person can live independently, but often needs help to raise children and can only work at simple supervised jobs.	0.043 (0.026–0.064)
Intellectual disability, moderate	Has low intelligence, and is slow in learning to speak and to do even simple tasks. As an adult, the person requires a lot of support to live independently and raise children. The person can only work at the simplest supervised jobs.	0.100 (0.066–0.142)
Intellectual disability, severe	Has very low intelligence and cannot speak more than a few words, needs constant supervision and help with most daily activities, and can do only the simplest tasks.	0.160 (0.107–0.226)
Intellectual disability, profound	Has very low intelligence, has almost no language, and does not understand even the most basic requests or instructions. The person requires constant supervision and help for all activities.	0.200 (0.133–0.283)

Table S5: Age-standardised prevalence of ASD per 100 000 persons by location and sex, 2021

Location	Both (95% UI)	Female (95% UI)	Male (95% UI)
**Global**	**788·3 (663·8-927·2)**	**508·1 (424·6-604·3)**	**1064·7 (898·5-1245·7)**
**Central Europe, Eastern Europe, and Central Asia**	**927·5 (778·5-1095·3)**	**644·2 (538·2-764·5)**	**1218·6 (1021·9-1440·2)**
**Central Asia**	**886·0 (744·2-1044·4)**	**621·5 (517·0-731·8)**	**1154·4 (971·2-1363·8)**
Armenia	922·0 (766·6-1090·5)	644·7 (534·4-773·4)	1204·2 (990·9-1435·4)
Azerbaijan	893·0 (745·1-1051·2)	625·2 (514·4-743·0)	1154·9 (952·7-1373·4)
Georgia	910·7 (760·4-1082·5)	636·5 (526·1-757·9)	1185·6 (983·0-1422·0)
Kazakhstan	897·7 (752·8-1064·9)	630·2 (520·8-766·7)	1180·0 (978·9-1405·8)
Kyrgyzstan	888·8 (746·0-1060·8)	623·7 (513·5-753·1)	1163·5 (959·8-1387·3)
Mongolia	861·3 (726·4-1027·4)	607·3 (504·8-722·6)	1124·8 (937·7-1351·1)
Tajikistan	866·8 (726·9-1023·6)	608·4 (500·9-733·1)	1119·8 (936·6-1327·2)
Turkmenistan	884·6 (740·3-1043·2)	615·7 (507·0-734·8)	1143·6 (955·7-1350·4)
Uzbekistan	879·4 (733·2-1035·4)	616·1 (506·8-742·6)	1144·3 (950·1-1370·2)
**Central Europe**	**964·4 (810·0-1140·6)**	**662·3 (552·2-785·0)**	**1263·3 (1058·4-1489·7)**
Albania	944·0 (781·6-1112·3)	645·2 (530·5-783·0)	1231·3 (1023·8-1468·1)
Bosnia and Herzegovina	972·0 (806·8-1150·0)	669·7 (552·2-810·1)	1271·9 (1052·7-1521·6)
Bulgaria	936·7 (782·9-1115·1)	646·0 (532·5-770·8)	1223·0 (1011·7-1458·3)
Croatia	996·0 (824·9-1186·2)	676·3 (559·1-814·3)	1313·2 (1081·3-1578·0)
Czechia	996·7 (824·2-1172·9)	675·8 (553·6-809·8)	1311·2 (1088·5-1567·3)
Hungary	971·0 (803·5-1153·1)	665·4 (545·6-803·5)	1279·7 (1066·4-1521·7)
Montenegro	976·3 (811·1-1150·0)	665·1 (550·9-791·8)	1283·1 (1062·7-1531·8)

Location	Both (95% UI)	Female (95% UI)	Male (95% UI)
North Macedonia	957·5 (803·0-1136·9)	654·9 (537·9-780·7)	1246·2 (1049·0-1489·4)
Poland	964·5 (806·7-1144·4)	666·0 (552·7-796·1)	1263·5 (1060·0-1495·0)
Romania	939·0 (786·0-1105·1)	647·3 (533·4-777·2)	1227·6 (1023·2-1465·7)
Serbia	968·4 (801·4-1155·4)	662·2 (539·9-794·4)	1259·8 (1044·6-1506·8)
Slovakia	966·7 (809·0-1150·8)	664·2 (546·2-793·1)	1267·5 (1048·1-1532·1)
Slovenia	1017·9 (845·7-1224·1)	684·6 (565·8-830·5)	1338·0 (1102·5-1609·2)
**Eastern Europe**	**928·5 (779·8-1102·3)**	**645·6 (535·4-773·4)**	**1226·2 (1031·6-1457·2)**
Belarus	945·2 (783·5-1128·1)	653·8 (534·7-784·2)	1250·3 (1043·9-1493·3)
Estonia	964·5 (811·2-1146·7)	658·9 (540·8-793·0)	1272·7 (1057·4-1530·9)
Latvia	943·2 (778·7-1117·1)	650·9 (533·7-776·3)	1244·6 (1031·5-1490·3)
Lithuania	944·8 (783·9-1115·8)	650·8 (541·5-786·5)	1249·6 (1032·5-1488·6)
Republic of Moldova	917·9 (766·0-1082·3)	636·5 (525·8-761·1)	1206·3 (998·2-1447·8)
Russian Federation	928·8 (780·0-1100·6)	645·8 (536·5-770·2)	1227·6 (1028·5-1451·3)
Ukraine	922·3 (770·6-1101·3)	642·9 (532·1-767·0)	1214·5 (1017·5-1454·2)
**High-income**	**1090·2 (916·3-1279·3)**	**649·8 (543·4-771·1)**	**1526·5 (1283·2-1786·5)**
**Australasia**	**1191·0 (993·0-1422·7)**	**709·4 (586·1-862·2)**	**1670·0 (1387·5-2012·1)**
Australia	1195·4 (996·1-1428·7)	711·2 (581·7-865·0)	1678·7 (1388·8-2028·0)
New Zealand	1169·2 (969·9-1404·3)	700·8 (576·7-841·3)	1627·8 (1357·1-1949·0)
**High-income Asia Pacific**	**1559·5 (1311·3-1832·4)**	**938·8 (790·5-1107·1)**	**2161·1 (1815·5-2536·3)**
Brunei Darussalam	1370·5 (1153·1-1635·4)	834·2 (688·5-997·0)	1856·1 (1537·8-2204·7)
Japan	1586·9 (1333·2-1864·1)	944·3 (792·8-1119·9)	2214·1 (1870·6-2597·1)
Republic of Korea	1506·8 (1254·0-1779·2)	933·2 (770·4-1112·6)	2055·7 (1708·9-2427·4)

Location	Both (95% UI)	Female (95% UI)	Male (95% UI)
Singapore	1487·3 (1250·0-1767·8)	891·2 (731·1-1072·2)	2065·9 (1732·9-2448·6)
**High-income North America**	**1097·2 (919·2-1296·7)**	**707·3 (589·5-838·0)**	**1486·9 (1244·5-1746·3)**
Canada	1117·9 (930·4-1317·6)	717·5 (591·8-861·7)	1515·1 (1256·0-1805·5)
Greenland	1016·9 (848·6-1199·3)	660·8 (544·1-797·4)	1343·5 (1119·7-1607·1)
United States of America	1094·8 (920·2-1291·2)	706·2 (588·3-836·0)	1483·7 (1245·9-1748·4)
**Southern Latin America**	**1056·5 (885·9-1245·4)**	**662·3 (548·0-787·7)**	**1458·7 (1216·8-1729·8)**
Argentina	1045·7 (867·3-1230·9)	657·4 (540·9-788·1)	1443·9 (1198·8-1712·9)
Chile	1082·6 (902·8-1277·0)	673·8 (554·0-807·8)	1493·0 (1248·8-1776·1)
Uruguay	1058·1 (880·2-1268·2)	664·7 (551·4-796·7)	1464·4 (1209·8-1753·5)
**Western Europe**	**896·6 (751·6-1054·5)**	**477·6 (397·7-572·1)**	**1309·1 (1099·7-1533·1)**
Andorra	905·9 (752·9-1077·5)	484·5 (395·0-587·6)	1303·1 (1082·4-1549·0)
Austria	892·2 (741·1-1069·1)	486·1 (398·1-590·0)	1287·1 (1065·9-1547·0)
Belgium	885·3 (734·8-1051·9)	484·0 (398·7-587·6)	1281·5 (1056·5-1525·1)
Cyprus	882·9 (733·7-1048·0)	482·9 (397·0-584·1)	1281·0 (1058·4-1522·8)
Denmark	946·5 (797·2-1124·2)	514·9 (417·0-614·4)	1366·3 (1143·1-1620·4)
Finland	828·7 (687·5-990·5)	473·3 (390·4-568·1)	1173·2 (971·0-1407·7)
France	881·3 (734·9-1049·9)	482·0 (395·3-582·3)	1283·7 (1064·0-1528·3)
Germany	897·5 (738·7-1071·0)	484·4 (400·6-584·8)	1289·2 (1053·0-1542·4)
Greece	881·4 (732·5-1056·8)	485·4 (397·3-586·6)	1278·6 (1057·6-1541·6)
Iceland	1047·3 (875·1-1235·0)	564·4 (464·5-665·7)	1509·4 (1261·8-1786·7)
Ireland	1071·9 (894·8-1274·4)	596·8 (490·0-719·7)	1548·1 (1292·1-1850·2)
Israel	795·1 (665·4-944·0)	446·8 (365·6-536·6)	1140·3 (950·2-1353·7)

Location	Both (95% UI)	Female (95% UI)	Male (95% UI)
Italy	999·3 (839·6-1186·0)	558·9 (462·6-667·7)	1431·6 (1199·2-1699·1)
Luxembourg	901·8 (749·7-1081·9)	487·1 (400·5-591·5)	1300·6 (1083·4-1559·3)
Malta	898·1 (751·3-1069·8)	484·5 (397·1-591·4)	1292·9 (1079·4-1546·0)
Monaco	890·6 (744·5-1062·5)	481·5 (396·4-581·7)	1305·2 (1079·6-1567·0)
Netherlands	899·6 (739·8-1073·2)	486·3 (397·2-583·3)	1305·5 (1082·7-1558·2)
Norway	808·3 (677·1-963·1)	444·6 (367·0-532·4)	1155·5 (971·0-1370·1)
Portugal	861·8 (718·3-1019·8)	476·3 (389·8-570·1)	1257·4 (1047·4-1507·6)
San Marino	890·8 (739·0-1063·6)	485·3 (398·8-584·6)	1303·7 (1078·8-1550·8)
Spain	1024·7 (863·8-1204·3)	451·7 (376·4-538·1)	1591·6 (1331·8-1870·9)
Sweden	1016·7 (839·5-1216·8)	501·7 (397·9-627·3)	1508·5 (1250·3-1803·0)
Switzerland	902·3 (749·2-1073·8)	485·2 (398·4-585·5)	1304·9 (1076·5-1560·6)
United Kingdom	750·8 (631·6-890·3)	404·1 (332·7-483·9)	1099·8 (923·1-1298·4)
**Latin America and Caribbean**	**689·5 (579·7-820·3)**	**464·9 (386·3-557·9)**	**920·2 (775·0-1087·0)**
**Andean Latin America**	**684·3 (571·6-814·1)**	**464·5 (385·3-555·9)**	**902·4 (751·0-1069·7)**
Bolivia (Plurinational State of)	663·1 (555·1-795·5)	453·8 (374·6-545·8)	872·5 (724·1-1043·4)
Ecuador	682·7 (565·9-813·8)	465·8 (385·8-557·8)	901·3 (747·6-1075·8)
Peru	691·7 (577·7-826·2)	467·0 (384·7-561·3)	912·3 (757·3-1086·9)
**Caribbean**	**682·5 (572·1-813·8)**	**464·7 (382·7-558·3)**	**902·7 (763·6-1069·7)**
Antigua and Barbuda	711·6 (588·0-844·9)	482·3 (392·1-583·0)	946·2 (786·6-1123·1)
Bahamas	692·9 (572·2-823·3)	476·7 (390·4-573·4)	921·0 (761·0-1093·3)
Barbados	712·4 (593·8-854·6)	482·7 (395·4-581·3)	948·7 (789·9-1141·0)
Belize	683·0 (567·1-821·9)	467·6 (382·0-567·0)	900·7 (744·2-1084·0)

Location	Both (95% UI)	Female (95% UI)	Male (95% UI)
Bermuda	740·5 (611·9-893·6)	492·1 (403·0-593·1)	993·2 (818·9-1203·4)
Cuba	723·4 (603·2-859·9)	485·0 (396·7-583·8)	955·8 (793·5-1133·9)
Dominica	695·0 (582·7-829·6)	472·5 (389·4-564·8)	908·3 (752·3-1079·5)
Dominican Republic	677·1 (565·5-807·5)	461·6 (375·7-556·8)	889·4 (742·3-1068·3)
Grenada	697·9 (578·4-825·5)	472·2 (388·7-571·4)	911·7 (754·6-1083·3)
Guyana	663·2 (552·8-789·0)	457·4 (379·3-548·8)	874·2 (727·6-1040·1)
Haiti	627·0 (522·7-746·2)	431·8 (355·1-517·7)	831·2 (692·6-988·7)
Jamaica	702·5 (590·2-834·7)	476·1 (392·3-569·8)	929·9 (780·8-1102·4)
Puerto Rico	731·1 (602·8-877·6)	494·4 (404·7-591·3)	977·4 (807·0-1168·2)
Saint Kitts and Nevis	694·9 (581·7-824·3)	472·0 (387·2-567·6)	917·1 (765·7-1100·0)
Saint Lucia	702·4 (581·6-837·4)	477·2 (389·9-574·1)	923·4 (761·7-1101·2)
Saint Vincent and the Grenadines	693·7 (575·6-825·8)	469·5 (385·6-562·9)	908·9 (752·4-1079·8)
Suriname	673·7 (559·5-804·7)	460·6 (378·3-549·2)	889·6 (739·1-1066·8)
Trinidad and Tobago	699·2 (585·7-835·9)	474·4 (390·3-570·6)	919·8 (757·2-1097·9)
United States Virgin Islands	709·9 (588·3-848·5)	481·5 (394·8-579·3)	941·3 (782·3-1126·4)
**Central Latin America**	**758·6 (639·0-897·8)**	**510·3 (423·1-611·4)**	**1017·1 (855·1-1196·5)**
Colombia	771·8 (640·9-921·5)	516·5 (423·3-617·4)	1032·7 (857·0-1234·4)
Costa Rica	792·0 (655·2-946·8)	532·5 (438·7-642·7)	1064·9 (876·9-1276·4)
El Salvador	745·6 (620·6-893·8)	508·5 (417·7-608·6)	1012·9 (842·9-1213·5)
Guatemala	726·0 (605·5-861·0)	494·6 (409·7-593·1)	973·5 (807·9-1153·6)
Honduras	727·8 (605·8-864·2)	494·0 (407·5-588·5)	977·1 (813·4-1165·7)
Mexico	758·5 (641·3-902·8)	509·7 (424·2-611·1)	1017·3 (859·7-1201·7)

Location	Both (95% UI)	Female (95% UI)	Male (95% UI)
Nicaragua	756·1 (627·4-892·4)	508·9 (418·1-609·0)	1010·5 (835·5-1197·4)
Panama	779·7 (654·4-921·4)	516·6 (427·3-620·4)	1037·1 (870·0-1229·7)
Venezuela (Bolivarian Republic of)	756·7 (634·2-899·5)	511·3 (422·7-611·3)	1016·5 (845·7-1209·9)
**Tropical Latin America**	**614·5 (514·7-732·3)**	**413·4 (340·9-495·8)**	**820·7 (687·6-974·5)**
Brazil	614·1 (514·6-731·9)	413·1 (340·4-495·2)	820·4 (687·4-973·8)
Paraguay	628·1 (521·4-748·2)	421·5 (344·0-509·7)	830·2 (687·0-988·0)
**North Africa and Middle East**	**771·8 (648·5-910·7)**	**524·8 (436·1-624·1)**	**1001·3 (845·9-1179·2)**
Afghanistan	703·1 (581·7-839·5)	488·0 (403·4-585·1)	917·8 (757·1-1094·5)
Algeria	774·6 (644·9-921·9)	530·5 (437·3-634·0)	1010·7 (840·1-1205·1)
Bahrain	848·4 (703·7-1009·1)	545·1 (450·4-661·6)	1049·3 (872·7-1251·0)
Egypt	763·7 (639·6-905·8)	522·7 (430·9-629·7)	987·0 (821·5-1167·5)
Iran (Islamic Republic of)	791·2 (662·9-934·0)	527·7 (439·2-631·4)	1045·7 (875·9-1232·2)
Iraq	709·2 (590·7-852·8)	495·4 (407·9-600·2)	909·2 (757·3-1098·5)
Jordan	810·0 (672·7-960·5)	541·2 (444·0-650·3)	1044·1 (859·9-1253·8)
Kuwait	829·5 (685·6-984·3)	556·0 (454·3-670·2)	1072·6 (890·0-1276·3)
Lebanon	872·4 (724·2-1033·2)	610·6 (502·2-729·8)	1131·6 (934·4-1360·8)
Libya	769·1 (636·7-915·3)	526·4 (434·0-638·2)	999·6 (828·9-1193·3)
Morocco	756·2 (634·1-897·5)	520·6 (432·3-625·1)	987·9 (820·4-1167·9)
Oman	832·9 (687·4-998·6)	543·3 (444·5-651·6)	1035·7 (855·1-1246·2)
Palestine	782·1 (651·4-934·1)	539·1 (441·0-646·2)	1017·8 (847·0-1218·2)
Qatar	964·6 (798·0-1145·1)	583·3 (479·0-697·6)	1173·5 (972·7-1396·9)
Saudi Arabia	817·8 (677·7-975·7)	535·8 (442·2-638·3)	1027·2 (848·5-1228·3)

Location	Both (95% UI)	Female (95% UI)	Male (95% UI)
Sudan	737·5 (611·6-879·5)	506·5 (416·0-608·2)	960·2 (796·1-1151·5)
Syrian Arab Republic	759·0 (630·0-913·0)	532·3 (437·0-645·2)	1012·8 (838·0-1210·3)
Tunisia	785·0 (650·9-932·3)	540·9 (444·6-648·8)	1028·3 (846·0-1226·3)
Türkiye	786·6 (652·9-931·7)	538·7 (445·9-647·0)	1030·4 (848·7-1227·3)
United Arab Emirates	840·9 (701·0-999·6)	530·7 (436·3-641·9)	1014·2 (844·5-1210·2)
Yemen	726·6 (608·1-859·7)	504·0 (420·6-601·1)	947·2 (788·8-1134·3)
**South Asia**	**686·2 (576·6-802·0)**	**466·8 (389·9-557·3)**	**897·0 (753·8-1038·9)**
Bangladesh	588·2 (486·7-696·6)	388·2 (316·4-466·6)	794·2 (654·0-941·2)
Bhutan	648·1 (540·6-770·4)	435·7 (360·0-525·6)	846·6 (702·6-1008·6)
India	708·1 (595·1-831·2)	483·7 (404·5-576·2)	921·4 (775·6-1073·9)
Nepal	626·1 (519·4-750·6)	431·5 (355·9-523·9)	840·9 (695·5-1006·7)
Pakistan	631·1 (526·6-741·9)	427·2 (355·0-512·9)	825·4 (687·8-970·5)
**Southeast Asia, East Asia, and Oceania**	**669·2 (560·7-791·5)**	**373·6 (308·4-447·9)**	**950·5 (797·1-1122·2)**
**East Asia**	**660·7 (549·4-785·9)**	**324·6 (267·3-391·9)**	**972·2 (811·3-1152·9)**
China	655·7 (545·1-780·4)	318·2 (262·1-384·3)	967·9 (807·4-1148·1)
Democratic People's Republic of Korea	706·1 (587·1-845·0)	427·5 (350·7-520·4)	980·8 (809·0-1175·7)
Taiwan (Province of China)	911·1 (752·0-1083·9)	587·6 (480·3-714·0)	1227·9 (1014·3-1471·6)
**Oceania**	**673·2 (566·1-807·0)**	**433·5 (356·8-523·6)**	**898·2 (751·4-1078·0)**
American Samoa	708·9 (588·9-847·7)	454·9 (370·2-550·3)	952·1 (785·5-1142·0)
Cook Islands	724·6 (601·4-867·4)	463·7 (382·2-554·4)	1000·3 (829·4-1199·4)
Fiji	684·8 (566·4-818·4)	445·0 (363·8-533·5)	919·1 (762·4-1093·5)
Guam	726·0 (600·3-871·4)	461·2 (375·4-561·1)	979·2 (809·6-1178·9)

Location	Both (95% UI)	Female (95% UI)	Male (95% UI)
Kiribati	647·3 (537·6-772·5)	428·1 (348·7-515·2)	879·9 (730·5-1043·6)
Marshall Islands	677·3 (562·0-808·1)	438·6 (360·9-527·3)	905·3 (748·1-1083·1)
Micronesia (Federated States of)	683·2 (575·0-804·0)	442·9 (363·1-526·6)	915·9 (759·3-1093·1)
Nauru	672·3 (560·1-802·9)	437·7 (360·8-527·8)	908·0 (752·8-1082·8)
Niue	701·6 (584·0-827·2)	455·5 (373·9-547·3)	941·0 (781·3-1112·3)
Northern Mariana Islands	734·2 (611·6-874·8)	462·6 (380·0-552·5)	979·9 (808·8-1176·8)
Palau	726·7 (601·9-857·4)	450·8 (370·9-549·4)	950·9 (787·0-1136·6)
Papua New Guinea	669·8 (562·0-804·3)	430·0 (352·9-520·8)	891·7 (744·8-1069·7)
Samoa	696·9 (580·5-829·9)	448·3 (365·1-541·4)	932·2 (779·7-1102·1)
Solomon Islands	671·8 (562·7-801·6)	436·5 (354·9-529·1)	899·2 (745·8-1075·9)
Tokelau	714·5 (598·7-855·6)	455·2 (374·3-550·5)	961·8 (797·4-1152·8)
Tonga	694·9 (575·6-829·3)	451·2 (368·3-547·0)	943·4 (778·0-1124·9)
Tuvalu	684·4 (566·4-820·7)	437·9 (358·0-527·9)	910·8 (750·2-1089·6)
Vanuatu	671·1 (555·5-802·4)	439·1 (360·9-524·2)	900·3 (739·7-1078·7)
**Southeast Asia**	**683·0 (574·6-809·7)**	**458·6 (380·6-551·4)**	**905·9 (762·1-1069·0)**
Cambodia	672·4 (560·6-805·8)	441·5 (361·5-530·6)	913·9 (753·8-1093·4)
Indonesia	647·3 (543·6-769·0)	478·4 (397·6-572·9)	811·8 (683·8-959·4)
Lao People's Democratic Republic	668·8 (557·4-802·7)	436·0 (358·4-530·1)	899·7 (748·8-1073·7)
Malaysia	734·3 (611·9-874·6)	467·9 (383·3-563·6)	983·6 (815·8-1168·5)
Maldives	765·9 (630·0-912·2)	458·1 (375·6-555·1)	983·9 (811·6-1183·4)
Mauritius	728·1 (603·1-862·5)	466·9 (384·5-559·8)	990·3 (818·0-1181·8)
Myanmar	668·8 (555·6-803·0)	439·7 (361·8-531·8)	913·2 (755·6-1095·0)

Location	Both (95% UI)	Female (95% UI)	Male (95% UI)
Philippines	686·1 (576·4-811·2)	446·3 (370·9-535·2)	920·3 (774·5-1081·9)
Seychelles	733·9 (607·9-873·3)	466·5 (382·8-554·5)	974·7 (805·6-1169·4)
Sri Lanka	749·9 (620·3-896·0)	466·8 (386·5-559·8)	1048·0 (863·0-1246·3)
Thailand	722·2 (597·6-864·5)	463·4 (381·4-560·7)	989·3 (819·3-1194·8)
Timor-Leste	677·3 (566·1-807·2)	440·3 (360·0-529·8)	909·6 (756·6-1094·7)
Viet Nam	726·3 (606·3-863·6)	421·2 (344·7-508·0)	1029·5 (849·9-1223·5)
**Sub-Saharan Africa**	**890·2 (748·6-1043·1)**	**628·4 (526·6-743·1)**	**1164·7 (980·2-1358·6)**
**Central Sub-Saharan Africa**	**885·4 (739·7-1046·5)**	**624·5 (517·9-742·2)**	**1152·0 (963·2-1364·4)**
Angola	879·2 (741·0-1029·7)	626·6 (520·3-751·9)	1155·0 (964·6-1366·6)
Central African Republic	848·7 (711·7-1026·1)	606·8 (497·6-730·6)	1105·6 (927·6-1328·6)
Congo	896·3 (750·2-1059·9)	630·2 (519·6-751·2)	1166·5 (971·8-1378·8)
Democratic Republic of the Congo	887·7 (738·2-1055·6)	623·8 (514·4-752·9)	1151·2 (961·9-1368·7)
Equatorial Guinea	927·4 (773·1-1096·9)	637·4 (527·0-772·6)	1193·1 (992·4-1421·9)
Gabon	905·4 (756·3-1079·4)	641·7 (530·6-771·0)	1189·5 (999·2-1417·2)
**Eastern Sub-Saharan Africa**	**893·5 (752·4-1045·2)**	**629·0 (529·0-744·7)**	**1165·9 (980·2-1363·2)**
Burundi	886·5 (737·5-1042·9)	619·2 (513·6-740·3)	1147·9 (948·7-1358·7)
Comoros	906·9 (757·5-1074·5)	636·1 (528·6-762·7)	1180·2 (978·4-1395·3)
Djibouti	931·6 (775·9-1106·1)	637·1 (525·9-765·9)	1182·7 (982·7-1396·7)
Eritrea	886·8 (738·3-1053·8)	626·4 (515·7-745·2)	1148·6 (957·2-1380·1)
Ethiopia	894·6 (751·9-1053·8)	626·5 (525·0-747·0)	1159·3 (972·9-1370·9)
Kenya	906·3 (761·4-1061·0)	637·3 (533·2-759·2)	1181·4 (995·8-1376·5)
Madagascar	889·5 (742·0-1051·7)	626·8 (517·9-749·5)	1161·7 (969·5-1383·9)

Location	Both (95% UI)	Female (95% UI)	Male (95% UI)
Malawi	886·0 (741·6-1048·2)	626·7 (522·2-757·6)	1164·0 (969·6-1382·5)
Mozambique	872·6 (725·0-1039·0)	621·8 (514·3-741·0)	1150·5 (961·4-1363·7)
Rwanda	890·7 (742·6-1054·9)	629·8 (521·5-753·7)	1173·4 (971·8-1394·2)
Somalia	868·1 (726·0-1020·8)	612·6 (508·2-733·0)	1125·4 (938·5-1339·9)
South Sudan	891·2 (745·9-1060·4)	625·5 (518·0-753·4)	1160·3 (966·8-1375·1)
Uganda	900·1 (749·7-1067·6)	634·6 (527·9-762·0)	1184·4 (988·4-1402·4)
United Republic of Tanzania	897·5 (749·1-1067·5)	634·2 (528·1-763·1)	1178·4 (975·9-1406·8)
Zambia	896·1 (747·3-1063·8)	630·3 (524·0-759·8)	1167·6 (968·7-1396·1)
**Southern Sub-Saharan Africa**	**903·6 (760·1-1069·2)**	**637·3 (533·0-765·6)**	**1184·9 (995·9-1388·5)**
Botswana	906·0 (757·3-1077·2)	638·0 (527·7-766·8)	1183·8 (987·9-1405·6)
Eswatini	886·6 (735·8-1057·9)	628·4 (515·8-748·7)	1163·8 (963·4-1394·5)
Lesotho	871·0 (727·7-1041·3)	622·1 (514·4-741·7)	1142·0 (955·8-1366·4)
Namibia	905·0 (757·2-1079·1)	642·4 (530·3-776·8)	1189·4 (991·4-1419·7)
South Africa	911·7 (765·2-1082·3)	640·5 (538·1-763·4)	1194·0 (999·0-1412·8)
Zimbabwe	873·9 (728·9-1041·4)	624·3 (516·6-745·4)	1151·6 (966·9-1381·9)
**Western Sub-Saharan Africa**	**886·2 (745·1-1045·6)**	**626·9 (524·2-740·0)**	**1162·9 (979·1-1363·7)**
Benin	889·0 (736·6-1059·5)	626·9 (519·0-745·6)	1164·2 (960·9-1381·9)
Burkina Faso	875·7 (736·9-1034·0)	621·2 (515·7-738·7)	1151·4 (959·6-1372·8)
Cabo Verde	951·6 (792·4-1131·9)	662·6 (544·0-785·9)	1236·8 (1026·7-1485·1)
Cameroon	893·1 (744·4-1057·7)	628·3 (517·1-758·8)	1161·2 (971·9-1382·4)
Chad	876·2 (727·5-1049·3)	617·3 (508·0-745·6)	1137·2 (942·2-1360·9)
Côte d'Ivoire	909·1 (752·9-1083·9)	629·2 (517·9-756·1)	1169·2 (963·6-1389·5)

Location	Both (95% UI)	Female (95% UI)	Male (95% UI)
Gambia	898·0 (752·1-1063·5)	631·2 (525·9-761·7)	1173·7 (970·6-1395·2)
Ghana	894·6 (748·0-1064·2)	632·8 (521·2-751·6)	1177·1 (985·2-1412·0)
Guinea	874·9 (729·5-1040·5)	619·6 (512·2-748·3)	1149·5 (954·8-1361·8)
Guinea-Bissau	862·5 (721·0-1036·5)	613·6 (505·2-739·6)	1131·2 (940·2-1350·8)
Liberia	905·2 (752·7-1072·2)	629·2 (515·9-756·0)	1171·7 (975·5-1391·4)
Mali	885·0 (739·1-1054·3)	622·4 (520·6-752·7)	1152·4 (954·4-1369·6)
Mauritania	914·3 (763·1-1085·7)	642·0 (529·6-760·9)	1198·4 (990·5-1426·7)
Niger	877·2 (729·4-1038·9)	617·7 (513·7-740·7)	1145·7 (951·2-1356·1)
Nigeria	881·9 (743·5-1040·4)	627·6 (525·0-742·5)	1164·9 (983·0-1371·4)
Sao Tome and Principe	923·5 (767·8-1089·2)	646·6 (532·7-776·3)	1199·3 (993·5-1421·0)
Senegal	899·7 (746·6-1068·3)	632·4 (522·7-756·9)	1169·9 (974·0-1396·0)
Sierra Leone	889·3 (739·9-1062·5)	624·1 (518·3-752·2)	1156·2 (953·1-1377·3)
Togo	887·3 (744·1-1059·9)	628·0 (519·5-759·3)	1165·3 (965·3-1393·6)

Note: UI = Uncertainty interval.

Table S6: Age-standardised prevalence of ASD sequelae per 100 000 persons by location, 2021

Location	ASD without ID (95% UI)	ASD with borderline ID (95% UI)	ASD with mild ID (95% UI)	ASD with moderate ID (95% UI)	ASD with severe ID (95% UI)	ASD with profound ID (95% UI)
**Global**	**352·4 (284·8-421·8)**	**155·3 (120·0-195·0)**	**117·5 (88·9-155·9)**	**109·0 (78·1-145·0)**	**43·4 (26·3-68·1)**	**10·8 (4·8-19·3)**
**Central Europe, Eastern Europe, and Central Asia**	**414·6 (334·9-494·8)**	**182·7 (140·5-229·0)**	**138·2 (104·3-182·9)**	**128·2 (91·9-168·7)**	**51·0 (30·9-80·2)**	**12·7 (5·6-22·8)**
**Central Asia**	**396·1 (323·1-473·3)**	**174·5 (134·6-220·0)**	**132·0 (100·3-174·4)**	**122·5 (87·6-162·5)**	**48·7 (29·6-76·7)**	**12·2 (5·4-21·6)**
Armenia	412·2 (332·9-492·8)	181·6 (139·0-228·8)	137·4 (101·3-182·8)	127·5 (90·5-170·4)	50·7 (30·2-77·9)	12·7 (5·6-22·6)
Azerbaijan	399·3 (324·4-476·7)	175·9 (134·2-220·3)	133·1 (100·1-179·2)	123·4 (88·3-164·6)	49·1 (29·9-76·6)	12·3 (5·3-22·0)
Georgia	407·1 (328·6-485·4)	179·4 (136·8-226·8)	135·8 (101·5-184·2)	125·9 (89·7-167·3)	50·1 (30·5-79·8)	12·5 (5·5-22·3)
Kazakhstan	401·3 (324·3-481·3)	176·8 (136·5-225·6)	133·8 (100·1-179·9)	124·1 (88·0-166·3)	49·4 (29·6-78·4)	12·3 (5·5-22·1)
Kyrgyzstan	397·3 (320·5-477·6)	175·1 (135·2-222·5)	132·4 (99·0-177·2)	122·8 (87·6-163·8)	48·9 (29·7-76·7)	12·2 (5·3-22·1)
Mongolia	385·0 (312·7-458·9)	169·6 (130·5-214·3)	128·3 (95·8-173·1)	119·1 (85·0-159·5)	47·4 (28·6-75·7)	11·8 (5·1-21·1)
Tajikistan	387·5 (313·8-463·9)	170·7 (130·2-214·9)	129·2 (96·1-171·6)	119·8 (85·1-159·9)	47·7 (28·6-75·4)	11·9 (5·2-20·8)
Turkmenistan	395·5 (319·0-476·2)	174·2 (132·9-220·6)	131·8 (99·1-176·6)	122·3 (87·5-164·1)	48·7 (28·9-77·3)	12·1 (5·3-21·4)

Location	ASD without ID (95% UI)	ASD with borderline ID (95% UI)	ASD with mild ID (95% UI)	ASD with moderate ID (95% UI)	ASD with severe ID (95% UI)	ASD with profound ID (95% UI)
Uzbekistan	393·1 (320·2-469·6)	173·2 (133·8-220·5)	131·0 (97·9-174·7)	121·6 (86·8-162·0)	48·4 (29·1-76·5)	12·1 (5·2-21·4)
**Central Europe**	**431·2 (347·0-517·4)**	**189·9 (145·7-239·0)**	**143·7 (107·9-189·3)**	**133·3 (95·6-177·5)**	**53·0 (32·1-83·5)**	**13·2 (5·8-23·8)**
Albania	422·0 (341·8-506·4)	185·9 (143·0-232·4)	140·7 (104·0-188·7)	130·5 (93·5-177·7)	51·9 (31·1-81·7)	13·0 (5·7-23·4)
Bosnia and Herzegovina	434·6 (351·6-519·1)	191·4 (147·8-242·1)	144·9 (107·1-194·6)	134·4 (95·9-179·8)	53·4 (32·0-84·3)	13·3 (5·9-24·3)
Bulgaria	418·8 (333·7-500·5)	184·5 (143·0-233·2)	139·6 (104·5-185·3)	129·5 (92·2-173·3)	51·5 (31·4-81·3)	12·9 (5·6-23·1)
Croatia	445·2 (358·0-536·6)	196·2 (151·7-248·9)	148·4 (109·3-199·6)	137·7 (98·1-184·0)	54·8 (33·3-86·5)	13·7 (6·0-24·6)
Czechia	445·6 (358·6-538·3)	196·3 (150·3-246·9)	148·5 (109·1-198·1)	137·7 (98·7-181·2)	54·8 (33·3-87·2)	13·7 (5·9-24·8)
Hungary	434·1 (352·7-520·1)	191·3 (147·0-242·8)	144·7 (107·6-193·5)	134·2 (95·5-180·6)	53·4 (31·8-84·8)	13·3 (5·9-23·9)
Montenegro	436·5 (351·0-521·9)	192·3 (147·2-243·2)	145·5 (106·1-194·3)	134·9 (96·6-182·7)	53·7 (32·3-85·1)	13·4 (5·9-24·1)
North Macedonia	428·1 (346·2-515·5)	188·6 (146·2-238·2)	142·7 (105·8-188·2)	132·3 (94·5-175·3)	52·7 (31·4-82·2)	13·2 (5·7-24·2)
Poland	431·2 (348·8-519·8)	189·9 (146·6-238·6)	143·7 (107·0-190·0)	133·3 (95·5-177·2)	53·1 (32·0-82·7)	13·2 (5·8-23·8)
Romania	419·8 (339·0-499·7)	184·9 (142·8-232·3)	139·9 (104·6-185·0)	129·8 (92·7-174·4)	51·7 (30·9-81·4)	12·9 (5·7-23·3)

Location	ASD without ID (95% UI)	ASD with borderline ID (95% UI)	ASD with mild ID (95% UI)	ASD with moderate ID (95% UI)	ASD with severe ID (95% UI)	ASD with profound ID (95% UI)
Serbia	433·0 (345·2-519·7)	190·7 (147·1-239·3)	144·3 (106·5-192·2)	133·9 (96·2-179·4)	53·3 (32·1-84·9)	13·3 (5·8-24·0)
Slovakia	432·2 (349·9-519·9)	190·4 (148·2-238·3)	144·1 (107·4-194·5)	133·6 (95·4-180·6)	53·2 (32·3-83·9)	13·3 (5·8-23·8)
Slovenia	455·1 (370·3-554·0)	200·5 (153·2-256·4)	151·7 (111·7-199·9)	140·7 (101·0-188·8)	56·0 (33·7-87·5)	14·0 (6·1-25·4)
**Eastern Europe**	**415·1 (333·9-495·1)**	**182·9 (140·9-229·1)**	**138·4 (103·7-182·8)**	**128·4 (92·0-170·0)**	**51·1 (30·8-80·4)**	**12·8 (5·6-22·9)**
Belarus	422·6 (343·0-505·8)	186·1 (143·8-230·5)	140·9 (104·2-185·6)	130·7 (93·5-173·5)	52·0 (31·6-80·4)	13·0 (5·7-23·3)
Estonia	431·2 (347·2-518·9)	190·0 (144·7-241·9)	143·7 (108·1-192·2)	133·3 (94·6-181·6)	53·0 (31·6-82·0)	13·3 (5·7-24·1)
Latvia	421·7 (337·7-502·5)	185·8 (144·0-233·7)	140·5 (104·0-185·5)	130·4 (92·9-174·0)	51·9 (31·6-81·5)	13·0 (5·7-23·6)
Lithuania	422·4 (341·7-505·9)	186·1 (140·5-234·0)	140·8 (105·0-189·2)	130·6 (92·7-176·7)	52·0 (31·6-80·6)	13·0 (5·7-23·2)
Republic of Moldova	410·4 (330·5-493·1)	180·8 (139·3-229·7)	136·7 (101·3-182·1)	126·9 (90·6-169·8)	50·5 (30·6-79·2)	12·6 (5·4-22·6)
Russian Federation	415·2 (335·3-497·3)	182·9 (140·8-229·8)	138·4 (103·7-183·7)	128·4 (92·2-169·8)	51·1 (31·1-80·3)	12·8 (5·6-22·9)
Ukraine	412·3 (331·1-495·1)	181·6 (139·0-226·6)	137·4 (103·1-183·1)	127·5 (90·8-168·8)	50·7 (30·1-81·7)	12·7 (5·5-22·5)
**High-income**	**487·4 (393·6-585·3)**	**214·7 (165·8-269·6)**	**162·5 (121·7-216·3)**	**150·7 (108·1-200·7)**	**60·0 (36·6-94·8)**	**15·0 (6·6-26·8)**

Location	ASD without ID (95% UI)	ASD with borderline ID (95% UI)	ASD with mild ID (95% UI)	ASD with moderate ID (95% UI)	ASD with severe ID (95% UI)	ASD with profound ID (95% UI)
**Australasia**	**532·4 (428·6-643·0)**	**234·5 (181·9-297·7)**	**177·5 (130·6-234·2)**	**164·7 (116·4-219·0)**	**65·5 (39·3-103·4)**	**16·4 (7·1-29·2)**
Australia	534·4 (430·1-647·9)	235·4 (182·1-298·9)	178·1 (130·7-235·8)	165·3 (116·7-219·9)	65·8 (39·4-104·0)	16·4 (7·1-29·4)
New Zealand	522·7 (426·1-631·9)	230·3 (180·3-291·0)	174·2 (129·1-229·5)	161·6 (115·0-213·6)	64·3 (38·7-100·7)	16·1 (7·0-28·5)
**High-income Asia Pacific**	**697·2 (561·8-839·6)**	**307·1 (240·2-388·1)**	**232·4 (173·2-307·8)**	**215·6 (153·8-286·8)**	**85·8 (52·1-135·0)**	**21·4 (9·4-38·3)**
Brunei Darussalam	612·7 (496·0-732·5)	269·9 (209·3-339·4)	204·3 (151·9-273·8)	189·5 (134·9-250·2)	75·4 (45·9-120·4)	18·8 (8·3-33·7)
Japan	709·5 (570·9-852·6)	312·5 (242·6-397·2)	236·4 (176·7-313·4)	219·4 (156·5-294·2)	87·3 (53·0-137·4)	21·8 (9·6-39·0)
Republic of Korea	673·7 (536·7-812·5)	296·7 (230·0-369·8)	224·5 (166·9-296·1)	208·3 (149·9-279·5)	82·8 (50·5-129·3)	20·7 (9·0-37·4)
Singapore	664·9 (540·3-795·2)	292·9 (226·3-368·9)	221·6 (164·2-288·6)	205·7 (147·6-275·5)	81·8 (49·1-129·7)	20·4 (8·9-36·7)
**High-income North America**	**490·5 (395·5-588·5)**	**216·1 (166·7-270·7)**	**163·5 (123·3-217·9)**	**151·7 (108·8-202·4)**	**60·3 (36·9-95·3)**	**15·1 (6·6-27·0)**
Canada	499·8 (403·2-597·8)	220·2 (171·3-276·9)	166·6 (122·3-225·7)	154·6 (110·7-208·5)	61·5 (37·0-97·1)	15·4 (6·8-27·7)
Greenland	454·6 (369·0-547·9)	200·3 (154·5-252·3)	151·5 (111·7-201·3)	140·6 (100·0-188·5)	55·9 (33·6-87·4)	14·0 (6·0-25·0)
United States of America	489·5 (395·2-587·9)	215·6 (166·5-269·9)	163·1 (123·2-216·9)	151·4 (108·3-202·0)	60·2 (36·8-95·3)	15·0 (6·6-26·9)

Location	ASD without ID (95% UI)	ASD with borderline ID (95% UI)	ASD with mild ID (95% UI)	ASD with moderate ID (95% UI)	ASD with severe ID (95% UI)	ASD with profound ID (95% UI)
**Southern Latin America**	**472·3 (384·1-562·9)**	**208·1 (161·0-262·4)**	**157·4 (116·8-212·7)**	**146·1 (104·6-196·0)**	**58·1 (35·6-92·7)**	**14·5 (6·4-25·8)**
Argentina	467·5 (378·7-557·3)	205·9 (159·4-261·2)	155·8 (114·6-211·9)	144·6 (102·7-194·7)	57·5 (34·8-92·2)	14·4 (6·2-25·6)
Chile	484·0 (391·9-582·4)	213·2 (164·8-270·2)	161·3 (119·7-214·3)	149·7 (106·5-198·6)	59·5 (36·2-94·3)	14·9 (6·5-26·7)
Uruguay	473·0 (378·5-565·5)	208·4 (161·6-263·4)	157·6 (116·4-207·9)	146·3 (104·9-197·2)	58·2 (35·1-92·0)	14·5 (6·3-26·3)
**Western Europe**	**400·8 (323·3-480·5)**	**176·6 (136·6-220·9)**	**133·6 (99·8-178·8)**	**124·0 (89·1-164·8)**	**49·3 (30·1-77·7)**	**12·3 (5·4-22·1)**
Andorra	405·0 (325·4-486·6)	178·4 (136·8-225·4)	135·0 (100·2-178·5)	125·2 (89·7-167·5)	49·8 (29·9-77·7)	12·4 (5·5-22·4)
Austria	398·9 (316·5-479·6)	175·7 (134·0-220·1)	133·0 (99·1-180·1)	123·3 (88·3-163·3)	49·1 (29·5-78·9)	12·2 (5·4-21·8)
Belgium	395·8 (319·9-474·6)	174·4 (135·1-221·7)	131·9 (97·5-178·6)	122·4 (86·6-166·6)	48·7 (29·0-76·6)	12·2 (5·4-22·2)
Cyprus	394·7 (318·4-472·4)	173·8 (134·4-218·7)	131·6 (97·7-175·0)	122·0 (86·8-162·0)	48·6 (29·2-77·5)	12·1 (5·3-21·7)
Denmark	423·2 (344·7-508·7)	186·4 (143·7-237·0)	141·0 (104·4-188·9)	130·9 (94·6-179·4)	52·0 (30·8-81·0)	13·0 (5·6-23·5)
Finland	370·5 (300·1-447·8)	163·2 (126·2-202·8)	123·5 (92·2-165·1)	114·6 (82·2-154·7)	45·6 (27·6-70·7)	11·4 (5·0-20·5)
France	394·0 (318·5-470·3)	173·5 (134·4-216·5)	131·4 (97·3-177·2)	121·9 (87·5-163·5)	48·5 (29·3-77·1)	12·1 (5·3-21·4)

Location	ASD without ID (95% UI)	ASD with borderline ID (95% UI)	ASD with mild ID (95% UI)	ASD with moderate ID (95% UI)	ASD with severe ID (95% UI)	ASD with profound ID (95% UI)
Germany	401·2 (319·0-481·0)	176·8 (135·9-222·9)	133·8 (98·4-180·9)	124·0 (88·3-165·8)	49·3 (30·0-78·2)	12·3 (5·3-22·5)
Greece	394·1 (315·1-473·7)	173·6 (135·7-220·6)	131·4 (97·6-174·6)	121·8 (86·9-163·9)	48·5 (29·1-76·6)	12·1 (5·3-21·7)
Iceland	468·2 (382·8-564·1)	206·3 (157·4-259·3)	156·1 (117·0-206·4)	144·8 (103·6-191·8)	57·5 (34·6-87·9)	14·4 (6·2-25·7)
Ireland	479·3 (383·5-583·6)	211·1 (163·6-268·1)	159·8 (119·3-214·5)	148·2 (106·4-198·0)	58·9 (35·5-92·2)	14·7 (6·4-26·7)
Israel	355·5 (287·0-427·4)	156·6 (119·5-198·0)	118·5 (87·1-159·8)	109·9 (78·6-152·0)	43·7 (26·3-68·6)	10·9 (4·8-19·5)
Italy	446·7 (363·4-534·1)	196·8 (151·4-246·9)	148·9 (111·8-199·6)	138·2 (98·5-184·8)	55·0 (33·3-86·1)	13·7 (6·1-24·6)
Luxembourg	403·2 (322·9-482·4)	177·5 (137·1-223·1)	134·4 (99·6-182·6)	124·7 (89·5-168·8)	49·6 (30·0-77·7)	12·4 (5·4-21·6)
Malta	401·5 (324·2-483·5)	176·9 (132·5-223·7)	133·8 (99·3-182·4)	124·1 (89·4-165·6)	49·4 (29·8-77·8)	12·3 (5·4-22·2)
Monaco	398·2 (322·6-478·8)	175·4 (135·3-222·8)	132·7 (98·9-178·7)	123·1 (88·2-165·6)	49·0 (29·8-77·9)	12·2 (5·4-22·3)
Netherlands	402·2 (323·5-483·5)	177·2 (137·5-223·2)	134·1 (99·1-181·2)	124·4 (87·5-167·0)	49·5 (29·9-78·3)	12·3 (5·4-21·8)
Norway	361·3 (291·4-434·2)	159·2 (122·6-198·8)	120·5 (90·1-160·5)	111·7 (80·1-149·4)	44·4 (27·0-70·2)	11·1 (4·9-20·0)
Portugal	385·3 (310·2-461·3)	169·7 (131·9-213·7)	128·4 (95·5-170·8)	119·1 (85·3-158·5)	47·4 (28·5-75·6)	11·8 (5·2-20·7)

Location	ASD without ID (95% UI)	ASD with borderline ID (95% UI)	ASD with mild ID (95% UI)	ASD with moderate ID (95% UI)	ASD with severe ID (95% UI)	ASD with profound ID (95% UI)
San Marino	398·2 (320·7-475·8)	175·4 (134·3-222·8)	132·8 (97·4-180·4)	123·1 (87·8-163·7)	49·0 (29·8-77·3)	12·2 (5·5-21·9)
Spain	458·2 (367·7-548·3)	201·8 (157·6-251·0)	152·7 (114·6-205·0)	141·7 (101·9-190·7)	56·3 (33·7-88·8)	14·1 (6·1-25·7)
Sweden	454·5 (361·8-555·9)	200·2 (153·9-255·5)	151·5 (112·2-201·3)	140·6 (100·4-189·9)	55·9 (33·5-87·1)	14·0 (6·1-25·7)
Switzerland	403·4 (324·5-481·2)	177·7 (136·8-224·9)	134·5 (98·6-183·6)	124·7 (87·7-164·7)	49·6 (29·9-78·7)	12·4 (5·3-22·6)
United Kingdom	335·7 (270·5-403·5)	147·9 (113·4-185·7)	111·9 (83·8-149·4)	103·8 (74·3-139·5)	41·3 (25·1-65·1)	10·3 (4·6-18·6)
**Latin America and Caribbean**	**308·3 (249·8-369·6)**	**135·8 (103·6-170·6)**	**102·7 (77·8-137·0)**	**95·3 (68·2-127·0)**	**37·9 (23·0-59·8)**	**9·5 (4·2-16·9)**
**Andean Latin America**	**305·9 (248·1-368·3)**	**134·8 (104·1-169·4)**	**102·0 (76·7-137·7)**	**94·6 (67·2-126·2)**	**37·6 (22·7-59·6)**	**9·4 (4·1-16·7)**
Bolivia (Plurinational State of)	296·5 (237·5-359·3)	130·6 (101·2-165·1)	98·8 (74·0-134·7)	91·7 (65·6-123·7)	36·5 (21·9-58·5)	9·1 (3·9-16·4)
Ecuador	305·2 (246·2-369·2)	134·4 (103·2-168·9)	101·7 (75·8-133·9)	94·4 (66·6-125·5)	37·6 (22·8-60·0)	9·4 (4·1-16·7)
Peru	309·2 (250·6-370·6)	136·2 (104·7-172·8)	103·1 (76·5-139·4)	95·6 (68·3-127·7)	38·0 (23·0-60·0)	9·5 (4·1-16·8)
**Caribbean**	**305·1 (245·9-364·5)**	**134·4 (103·2-169·4)**	**101·7 (76·5-134·9)**	**94·4 (67·4-125·9)**	**37·5 (22·7-59·7)**	**9·4 (4·1-16·8)**
Antigua and Barbuda	318·1 (254·7-381·4)	140·1 (106·2-179·3)	106·1 (78·0-142·5)	98·4 (70·3-131·0)	39·1 (23·6-61·7)	9·8 (4·2-17·8)

Location	ASD without ID (95% UI)	ASD with borderline ID (95% UI)	ASD with mild ID (95% UI)	ASD with moderate ID (95% UI)	ASD with severe ID (95% UI)	ASD with profound ID (95% UI)
Bahamas	309·8 (250·9-372·3)	136·4 (105·6-171·4)	103·2 (77·3-139·6)	95·8 (68·5-127·7)	38·1 (23·0-60·1)	9·5 (4·1-17·1)
Barbados	318·4 (256·8-383·9)	140·3 (107·6-176·8)	106·2 (79·2-141·9)	98·5 (70·2-133·0)	39·2 (23·8-61·7)	9·8 (4·3-17·6)
Belize	305·4 (247·0-369·9)	134·5 (102·9-170·6)	101·8 (75·2-135·6)	94·4 (67·5-126·4)	37·6 (22·6-58·7)	9·4 (4·1-17·0)
Bermuda	331·0 (268·4-402·6)	145·8 (111·7-184·5)	110·4 (81·2-148·8)	102·3 (72·8-136·6)	40·7 (24·9-64·7)	10·2 (4·5-18·5)
Cuba	323·4 (261·2-386·2)	142·5 (108·1-179·9)	107·8 (80·4-141·9)	100·0 (71·3-134·1)	39·8 (24·2-61·6)	9·9 (4·3-18·0)
Dominica	310·7 (251·2-372·9)	136·9 (105·5-171·8)	103·6 (77·7-136·5)	96·1 (69·6-128·0)	38·2 (23·1-60·8)	9·6 (4·2-17·0)
Dominican Republic	302·7 (245·6-366·7)	133·3 (102·9-169·0)	100·9 (74·0-134·8)	93·6 (67·0-125·5)	37·2 (22·5-60·6)	9·3 (4·0-16·6)
Grenada	312·0 (253·6-374·2)	137·4 (106·4-173·0)	104·0 (77·0-138·5)	96·5 (68·7-130·7)	38·4 (23·6-59·9)	9·6 (4·2-17·2)
Guyana	296·5 (238·2-358·8)	130·6 (101·7-165·4)	98·8 (73·1-132·1)	91·7 (65·0-122·9)	36·5 (21·8-58·4)	9·1 (4·0-16·2)
Haiti	280·3 (225·2-336·4)	123·5 (93·6-157·7)	93·4 (69·8-125·5)	86·7 (61·9-115·8)	34·5 (20·3-54·8)	8·6 (3·7-15·2)
Jamaica	314·1 (253·3-379·3)	138·4 (107·2-174·3)	104·7 (77·4-139·4)	97·1 (69·7-129·6)	38·6 (23·0-60·6)	9·7 (4·2-17·4)
Puerto Rico	326·8 (265·4-391·2)	144·0 (111·0-178·4)	109·0 (79·9-143·8)	101·1 (71·4-137·2)	40·2 (23·9-63·3)	10·0 (4·3-18·5)

Location	ASD without ID (95% UI)	ASD with borderline ID (95% UI)	ASD with mild ID (95% UI)	ASD with moderate ID (95% UI)	ASD with severe ID (95% UI)	ASD with profound ID (95% UI)
Saint Kitts and Nevis	310·6 (251·8-372·8)	136·8 (105·3-173·1)	103·6 (77·0-139·4)	96·1 (68·4-130·2)	38·2 (23·6-60·1)	9·5 (4·1-17·2)
Saint Lucia	314·0 (252·2-374·9)	138·3 (105·7-174·7)	104·7 (77·2-138·8)	97·1 (69·0-130·1)	38·6 (23·4-60·8)	9·7 (4·2-17·3)
Saint Vincent and the Grenadines	310·1 (248·8-368·8)	136·6 (104·6-174·5)	103·4 (76·4-137·5)	95·9 (68·9-129·6)	38·2 (23·1-59·9)	9·5 (4·1-17·1)
Suriname	301·2 (242·7-361·2)	132·7 (102·9-169·1)	100·4 (74·2-132·1)	93·2 (65·8-125·2)	37·1 (22·3-57·8)	9·2 (4·1-16·7)
Trinidad and Tobago	312·5 (253·3-375·5)	137·7 (106·8-174·4)	104·2 (76·6-141·6)	96·7 (69·7-130·9)	38·4 (23·1-61·8)	9·6 (4·1-17·5)
United States Virgin Islands	317·4 (255·9-378·6)	139·8 (106·3-173·3)	105·8 (77·5-142·3)	98·1 (70·6-132·3)	39·0 (23·7-61·6)	9·7 (4·3-17·5)
**Central Latin America**	**339·1 (276·0-406·1)**	**149·4 (114·4-187·7)**	**113·0 (85·5-149·9)**	**104·9 (75·1-139·8)**	**41·7 (25·4-65·5)**	**10·4 (4·6-18·6)**
Colombia	345·1 (279·2-418·0)	152·0 (115·9-193·2)	115·0 (84·6-153·2)	106·7 (76·4-142·2)	42·4 (25·8-66·3)	10·6 (4·6-18·9)
Costa Rica	354·0 (286·2-425·5)	156·0 (118·8-198·6)	118·0 (87·1-155·6)	109·5 (78·2-147·0)	43·5 (26·1-68·5)	10·9 (4·8-19·6)
El Salvador	333·3 (269·9-398·6)	146·9 (112·2-186·9)	111·1 (82·6-146·4)	103·1 (74·2-138·1)	41·0 (24·6-65·0)	10·2 (4·4-18·4)
Guatemala	324·5 (263·2-388·4)	143·0 (108·5-178·2)	108·2 (80·0-145·1)	100·4 (71·0-134·8)	39·9 (24·0-63·2)	10·0 (4·4-17·9)
Honduras	325·4 (263·4-394·3)	143·3 (110·9-182·0)	108·4 (81·7-144·8)	100·6 (71·2-133·9)	40·0 (24·1-63·2)	10·0 (4·3-17·9)

Location	ASD without ID (95% UI)	ASD with borderline ID (95% UI)	ASD with mild ID (95% UI)	ASD with moderate ID (95% UI)	ASD with severe ID (95% UI)	ASD with profound ID (95% UI)
Mexico	339·1 (273·8-406·1)	149·4 (114·7-188·0)	113·0 (85·3-148·9)	104·9 (74·9-139·2)	41·7 (25·2-65·9)	10·4 (4·6-18·6)
Nicaragua	338·0 (271·5-409·6)	148·9 (115·1-186·3)	112·7 (83·8-153·5)	104·6 (73·9-139·7)	41·6 (25·1-65·9)	10·4 (4·5-18·6)
Panama	348·6 (279·7-419·1)	153·6 (118·0-192·3)	116·2 (85·9-156·1)	107·8 (77·6-142·8)	42·9 (26·0-68·3)	10·7 (4·6-19·2)
Venezuela (Bolivarian Republic of)	338·3 (272·6-405·3)	149·0 (114·6-189·4)	112·8 (83·7-150·7)	104·6 (74·6-142·0)	41·6 (25·1-65·1)	10·4 (4·5-18·7)
**Tropical Latin America**	**274·7 (221·2-329·6)**	**121·0 (92·5-152·1)**	**91·6 (69·0-122·5)**	**85·0 (61·0-113·5)**	**33·8 (20·4-53·2)**	**8·4 (3·7-15·1)**
Brazil	274·5 (221·1-329·3)	120·9 (92·4-152·1)	91·5 (68·9-122·4)	84·9 (60·9-113·4)	33·8 (20·4-53·2)	8·4 (3·7-15·1)
Paraguay	280·8 (227·6-338·6)	123·7 (95·2-156·1)	93·6 (68·8-123·2)	86·9 (61·4-116·0)	34·6 (20·6-54·7)	8·6 (3·8-15·7)
**North Africa and Middle East**	**345·0 (280·8-411·1)**	**152·0 (116·5-191·4)**	**115·0 (87·0-153·5)**	**106·7 (76·2-142·0)**	**42·4 (25·7-66·6)**	**10·6 (4·7-18·8)**
Afghanistan	314·3 (254·1-376·5)	138·5 (105·3-174·8)	104·8 (78·3-141·2)	97·2 (69·1-130·3)	38·7 (22·9-61·0)	9·7 (4·3-17·1)
Algeria	346·3 (282·0-418·7)	152·5 (117·9-192·6)	115·4 (84·4-154·3)	107·1 (76·4-143·2)	42·6 (25·8-67·6)	10·6 (4·6-18·9)
Bahrain	379·3 (307·9-455·2)	167·1 (129·0-212·0)	126·4 (93·0-168·0)	117·3 (83·5-158·8)	46·7 (28·3-72·7)	11·7 (5·0-21·3)
Egypt	341·4 (276·9-408·8)	150·4 (114·9-189·7)	113·8 (84·1-152·1)	105·6 (75·4-142·4)	42·0 (25·3-66·5)	10·5 (4·5-18·3)

Location	ASD without ID (95% UI)	ASD with borderline ID (95% UI)	ASD with mild ID (95% UI)	ASD with moderate ID (95% UI)	ASD with severe ID (95% UI)	ASD with profound ID (95% UI)
Iran (Islamic Republic of)	353·7 (285·9-423·5)	155·8 (119·7-195·8)	117·9 (88·6-156·2)	109·4 (78·4-145·4)	43·5 (26·3-68·2)	10·9 (4·8-19·4)
Iraq	317·0 (257·0-383·7)	139·7 (107·7-177·4)	105·7 (78·8-140·1)	98·0 (69·6-131·1)	39·0 (23·3-61·6)	9·7 (4·2-17·6)
Jordan	362·1 (290·2-432·2)	159·5 (122·4-201·5)	120·7 (88·5-161·0)	112·0 (80·6-149·8)	44·5 (26·9-70·1)	11·1 (4·9-19·8)
Kuwait	370·9 (302·6-447·3)	163·3 (124·0-203·3)	123·6 (90·7-164·7)	114·7 (82·2-154·5)	45·6 (27·3-71·4)	11·4 (5·0-20·9)
Lebanon	390·1 (314·9-470·5)	171·8 (131·7-216·0)	130·0 (95·5-173·6)	120·6 (85·9-160·2)	48·0 (28·7-75·2)	12·0 (5·2-21·3)
Libya	343·8 (277·5-412·7)	151·5 (115·7-193·1)	114·6 (85·3-154·2)	106·3 (75·8-141·0)	42·3 (25·1-67·4)	10·6 (4·6-19·0)
Morocco	338·1 (274·3-403·7)	148·9 (113·3-186·7)	112·7 (82·5-149·4)	104·5 (75·0-140·1)	41·6 (25·0-65·8)	10·4 (4·5-18·7)
Oman	372·3 (298·6-446·0)	164·0 (126·7-209·3)	124·1 (92·6-165·4)	115·2 (81·3-155·5)	45·8 (27·4-72·5)	11·4 (5·0-20·8)
Palestine	349·7 (283·0-419·1)	154·0 (118·7-196·1)	116·5 (86·6-156·3)	108·1 (76·7-144·2)	43·0 (26·2-67·4)	10·7 (4·7-19·1)
Qatar	431·3 (340·8-519·6)	190·0 (145·0-241·4)	143·7 (105·6-192·9)	133·3 (95·3-178·3)	53·0 (31·9-83·9)	13·3 (5·8-23·9)
Saudi Arabia	365·6 (295·6-434·8)	161·0 (125·3-205·5)	121·9 (90·0-164·9)	113·1 (80·3-151·5)	45·0 (26·6-71·8)	11·2 (5·0-20·5)
Sudan	329·7 (265·0-393·5)	145·3 (111·8-186·7)	109·9 (81·1-147·4)	101·9 (72·5-136·9)	40·6 (24·1-63·7)	10·1 (4·5-17·9)

Location	ASD without ID (95% UI)	ASD with borderline ID (95% UI)	ASD with mild ID (95% UI)	ASD with moderate ID (95% UI)	ASD with severe ID (95% UI)	ASD with profound ID (95% UI)
Syrian Arab Republic	339·3 (273·8-409·2)	149·5 (113·9-188·7)	113·1 (83·3-151·5)	104·9 (74·1-141·1)	41·8 (25·1-65·9)	10·4 (4·6-18·4)
Tunisia	350·9 (283·3-419·7)	154·6 (118·6-196·4)	117·0 (87·0-155·9)	108·6 (77·8-145·9)	43·2 (26·0-67·7)	10·8 (4·7-19·6)
Türkiye	351·7 (282·3-420·7)	154·9 (119·5-194·2)	117·2 (86·5-156·4)	108·7 (76·9-143·9)	43·2 (26·2-67·1)	10·8 (4·7-19·1)
United Arab Emirates	375·9 (304·3-452·9)	165·6 (127·8-211·3)	125·3 (92·6-169·4)	116·2 (83·1-156·3)	46·3 (27·8-72·5)	11·5 (5·1-20·7)
Yemen	324·8 (265·8-390·4)	143·1 (109·6-181·0)	108·2 (81·4-144·3)	100·5 (71·5-134·3)	40·0 (23·8-64·5)	10·0 (4·3-17·9)
**South Asia**	**306·8 (248·0-367·9)**	**135·1 (104·5-170·2)**	**102·2 (77·1-135·4)**	**94·9 (67·8-126·4)**	**37·7 (22·7-59·6)**	**9·4 (4·1-16·8)**
Bangladesh	263·0 (210·5-315·0)	115·8 (88·3-147·0)	87·7 (65·5-117·8)	81·3 (58·0-109·9)	32·3 (19·3-50·7)	8·1 (3·5-14·3)
Bhutan	289·7 (234·9-347·3)	127·7 (97·0-162·7)	96·6 (71·5-127·1)	89·6 (63·9-120·5)	35·6 (21·3-55·5)	8·9 (3·9-15·8)
India	316·6 (256·6-379·7)	139·5 (107·4-175·3)	105·5 (79·5-139·9)	97·9 (70·0-130·1)	38·9 (23·5-61·4)	9·7 (4·3-17·3)
Nepal	279·9 (223·3-336·4)	123·3 (94·3-155·8)	93·3 (69·6-123·3)	86·6 (62·4-115·6)	34·4 (20·7-54·6)	8·6 (3·7-15·2)
Pakistan	282·1 (227·5-341·1)	124·3 (96·8-158·3)	94·0 (70·5-124·3)	87·3 (62·4-117·5)	34·7 (20·9-55·0)	8·7 (3·8-15·5)
**Southeast Asia, East Asia, and Oceania**	**299·2 (240·6-360·3)**	**131·8 (101·1-166·6)**	**99·7 (75·1-132·6)**	**92·5 (66·2-124·3)**	**36·8 (22·3-57·7)**	**9·2 (4·0-16·5)**

Location	ASD without ID (95% UI)	ASD with borderline ID (95% UI)	ASD with mild ID (95% UI)	ASD with moderate ID (95% UI)	ASD with severe ID (95% UI)	ASD with profound ID (95% UI)
**East Asia**	**295·4 (237·9-356·4)**	**130·1 (99·5-165·2)**	**98·4 (73·2-131·1)**	**91·3 (65·2-123·1)**	**36·3 (22·0-56·5)**	**9·1 (4·0-16·4)**
China	293·2 (236·1-353·9)	129·2 (98·7-164·1)	97·7 (72·7-130·2)	90·6 (64·7-122·2)	36·1 (21·8-56·1)	9·0 (4·0-16·3)
Democratic People's Republic of Korea	315·7 (255·3-378·5)	139·0 (106·8-174·0)	105·2 (77·9-139·6)	97·6 (69·7-131·6)	38·9 (23·5-61·9)	9·7 (4·2-17·7)
Taiwan (Province of China)	407·3 (328·2-496·5)	179·5 (137·1-227·7)	135·8 (99·6-181·2)	125·9 (90·6-170·8)	50·1 (30·2-78·1)	12·5 (5·4-22·9)
**Oceania**	**301·0 (242·1-364·4)**	**132·6 (103·0-167·6)**	**100·3 (75·0-134·5)**	**93·1 (66·3-125·3)**	**37·0 (22·1-58·4)**	**9·2 (4·0-16·3)**
American Samoa	316·9 (257·4-381·8)	139·6 (106·7-177·9)	105·6 (78·1-140·7)	98·0 (69·2-132·5)	39·0 (23·7-61·6)	9·7 (4·2-17·8)
Cook Islands	323·9 (263·6-391·4)	142·7 (110·0-181·3)	108·0 (80·2-145·1)	100·2 (71·5-134·4)	39·9 (23·7-62·7)	10·0 (4·4-18·1)
Fiji	306·1 (246·2-367·5)	134·9 (104·3-170·1)	102·0 (76·0-135·1)	94·7 (67·5-125·8)	37·7 (22·8-59·5)	9·4 (4·1-16·8)
Guam	324·5 (263·1-394·8)	143·0 (109·2-183·8)	108·2 (79·8-145·1)	100·3 (71·0-135·3)	39·9 (24·3-62·9)	10·0 (4·4-18·3)
Kiribati	289·4 (232·8-350·1)	127·5 (97·6-159·9)	96·4 (71·9-126·6)	89·5 (64·3-120·8)	35·6 (21·5-57·1)	8·9 (3·9-16·0)
Marshall Islands	302·8 (242·8-362·1)	133·4 (102·9-168·6)	100·9 (74·6-135·6)	93·6 (66·6-125·4)	37·3 (22·3-59·0)	9·3 (4·1-16·8)
Micronesia (Federated States of)	305·4 (249·5-365·4)	134·5 (104·6-171·8)	101·8 (75·5-136·9)	94·4 (66·7-125·9)	37·6 (22·6-59·3)	9·4 (4·0-17·1)

Location	ASD without ID (95% UI)	ASD with borderline ID (95% UI)	ASD with mild ID (95% UI)	ASD with moderate ID (95% UI)	ASD with severe ID (95% UI)	ASD with profound ID (95% UI)
Nauru	300·6 (241·0-361·9)	132·4 (101·9-168·3)	100·2 (74·5-135·0)	92·9 (66·3-123·9)	37·0 (22·2-58·4)	9·2 (4·0-16·5)
Niue	313·6 (254·5-373·9)	138·2 (106·5-173·7)	104·6 (77·1-138·5)	97·0 (69·6-130·8)	38·6 (23·4-62·2)	9·6 (4·2-17·5)
Northern Mariana Islands	328·2 (264·9-395·1)	144·6 (111·1-182·8)	109·4 (80·0-146·9)	101·5 (72·8-137·0)	40·4 (24·3-63·4)	10·1 (4·3-18·3)
Palau	324·8 (262·7-390·6)	143·1 (111·7-182·4)	108·3 (81·4-147·1)	100·5 (71·4-133·8)	40·0 (24·2-63·6)	10·0 (4·3-18·0)
Papua New Guinea	299·5 (241·0-364·7)	131·9 (102·2-166·5)	99·8 (74·4-133·9)	92·6 (65·7-124·4)	36·8 (21·9-58·0)	9·2 (4·0-16·2)
Samoa	311·6 (252·6-373·8)	137·2 (106·4-172·2)	103·8 (76·9-137·1)	96·3 (69·3-128·6)	38·3 (23·3-59·3)	9·6 (4·1-17·2)
Solomon Islands	300·4 (242·4-361·2)	132·3 (102·2-168·8)	100·1 (75·5-134·9)	92·9 (66·3-125·3)	36·9 (22·4-59·5)	9·2 (4·0-16·5)
Tokelau	319·4 (256·4-382·4)	140·7 (108·0-177·2)	106·5 (78·5-141·8)	98·7 (69·7-132·0)	39·3 (23·9-61·7)	9·8 (4·3-17·5)
Tonga	310·6 (250·4-372·9)	136·9 (104·5-172·8)	103·6 (76·9-140·2)	96·0 (68·8-129·8)	38·2 (23·5-59·7)	9·5 (4·2-17·4)
Tuvalu	306·0 (245·7-365·9)	134·8 (104·3-171·4)	102·0 (75·9-136·6)	94·6 (67·3-128·1)	37·6 (22·5-59·5)	9·4 (4·1-16·9)
Vanuatu	300·0 (240·6-361·1)	132·2 (101·4-166·5)	100·0 (74·3-133·9)	92·8 (66·2-125·2)	36·9 (22·4-58·6)	9·2 (3·9-16·4)
**Southeast Asia**	**305·3 (246·5-365·3)**	**134·5 (103·7-169·5)**	**101·8 (76·7-135·2)**	**94·4 (67·7-126·0)**	**37·6 (22·8-58·9)**	**9·4 (4·1-16·8)**

Location	ASD without ID (95% UI)	ASD with borderline ID (95% UI)	ASD with mild ID (95% UI)	ASD with moderate ID (95% UI)	ASD with severe ID (95% UI)	ASD with profound ID (95% UI)
Cambodia	300·6 (240·6-362·6)	132·4 (102·6-167·7)	100·2 (75·2-135·0)	93·0 (66·2-124·4)	37·0 (22·4-58·7)	9·2 (4·0-16·3)
Indonesia	289·4 (236·3-347·4)	127·5 (97·9-160·3)	96·5 (72·8-128·9)	89·5 (64·0-119·5)	35·6 (21·5-55·5)	8·9 (3·9-15·8)
Lao People's Democratic Republic	299·0 (240·7-357·1)	131·7 (99·9-168·2)	99·6 (74·8-132·4)	92·5 (65·4-123·7)	36·8 (21·9-59·0)	9·2 (4·1-16·5)
Malaysia	328·3 (266·7-396·5)	144·6 (112·2-180·4)	109·4 (81·3-145·6)	101·5 (72·5-136·2)	40·4 (24·3-63·9)	10·1 (4·4-18·4)
Maldives	342·4 (276·6-410·4)	150·8 (117·3-190·4)	114·2 (84·2-152·0)	105·9 (75·5-141·2)	42·1 (24·9-66·3)	10·5 (4·6-18·6)
Mauritius	325·5 (264·5-390·9)	143·4 (112·0-179·0)	108·5 (80·7-145·0)	100·7 (71·8-136·9)	40·1 (24·2-63·5)	10·0 (4·4-18·2)
Myanmar	299·0 (244·4-361·8)	131·7 (101·1-167·3)	99·6 (73·8-132·0)	92·5 (66·0-124·9)	36·8 (21·9-59·1)	9·2 (4·0-16·6)
Philippines	306·7 (249·0-367·0)	135·1 (103·2-170·7)	102·2 (77·2-135·4)	94·9 (67·9-127·0)	37·7 (22·8-59·0)	9·4 (4·1-16·8)
Seychelles	328·1 (266·7-394·1)	144·5 (110·6-183·9)	109·4 (80·9-145·3)	101·4 (72·2-135·2)	40·4 (24·3-63·8)	10·1 (4·4-18·2)
Sri Lanka	335·3 (272·6-403·3)	147·7 (112·9-185·6)	111·8 (83·6-148·1)	103·6 (73·8-138·7)	41·2 (24·6-64·2)	10·3 (4·5-18·8)
Thailand	322·8 (257·4-392·2)	142·2 (108·1-181·6)	107·6 (79·5-141·0)	99·8 (71·8-134·3)	39·7 (23·4-62·8)	9·9 (4·3-18·1)
Timor-Leste	302·8 (245·2-367·6)	133·4 (102·6-168·9)	100·9 (74·7-133·2)	93·6 (66·2-126·6)	37·2 (22·2-58·4)	9·3 (4·0-17·0)

Location	ASD without ID (95% UI)	ASD with borderline ID (95% UI)	ASD with mild ID (95% UI)	ASD with moderate ID (95% UI)	ASD with severe ID (95% UI)	ASD with profound ID (95% UI)
Viet Nam	324·7 (258·4-389·6)	143·0 (111·4-182·1)	108·3 (80·3-146·6)	100·4 (71·8-134·3)	39·9 (24·2-62·9)	10·0 (4·4-17·7)
**Sub-Saharan Africa**	**398·0 (325·3-476·2)**	**175·3 (136·0-220·7)**	**132·6 (100·2-176·2)**	**123·1 (88·2-164·2)**	**49·0 (29·5-76·5)**	**12·2 (5·4-21·6)**
**Central Sub-Saharan Africa**	**395·8 (321·4-477·7)**	**174·4 (134·0-217·7)**	**131·9 (98·9-175·0)**	**122·4 (87·8-162·2)**	**48·7 (29·0-77·6)**	**12·2 (5·3-21·7)**
Angola	393·1 (317·4-467·9)	173·1 (133·1-216·7)	131·0 (97·8-173·7)	121·6 (87·3-163·1)	48·3 (28·6-76·2)	12·1 (5·2-21·3)
Central African Republic	379·4 (309·0-458·0)	167·2 (129·0-213·0)	126·4 (94·6-167·4)	117·4 (83·1-157·6)	46·7 (27·6-73·6)	11·6 (5·1-20·7)
Congo	400·7 (321·5-483·7)	176·5 (135·4-222·5)	133·5 (99·4-174·8)	123·9 (88·6-166·4)	49·3 (29·7-76·8)	12·3 (5·4-21·8)
Democratic Republic of the Congo	396·9 (318·9-484·1)	174·8 (133·9-219·8)	132·2 (99·0-177·2)	122·7 (88·0-163·5)	48·8 (29·1-78·0)	12·2 (5·3-21·7)
Equatorial Guinea	414·6 (335·0-499·1)	182·7 (141·4-231·0)	138·2 (102·6-183·1)	128·2 (90·9-171·2)	51·0 (30·3-79·4)	12·7 (5·6-22·8)
Gabon	404·7 (329·1-483·1)	178·3 (135·7-224·1)	134·9 (101·5-179·5)	125·2 (89·5-167·5)	49·8 (29·7-78·9)	12·4 (5·5-22·1)
**Eastern Sub-Saharan Africa**	**399·4 (325·1-475·2)**	**176·0 (136·1-221·4)**	**133·1 (100·3-177·0)**	**123·5 (89·1-165·1)**	**49·1 (29·5-77·0)**	**12·3 (5·4-21·6)**
Burundi	396·3 (320·2-472·0)	174·6 (135·4-219·7)	132·1 (98·2-176·0)	122·6 (88·4-165·8)	48·8 (28·7-77·6)	12·2 (5·3-21·7)
Comoros	405·5 (330·3-486·1)	178·6 (138·2-225·5)	135·1 (100·0-181·2)	125·4 (89·8-169·2)	49·9 (29·9-79·5)	12·5 (5·4-22·2)

Location	ASD without ID (95% UI)	ASD with borderline ID (95% UI)	ASD with mild ID (95% UI)	ASD with moderate ID (95% UI)	ASD with severe ID (95% UI)	ASD with profound ID (95% UI)
Djibouti	416·5 (334·6-500·5)	183·5 (140·6-232·1)	138·8 (103·5-185·4)	128·8 (92·0-173·7)	51·3 (30·9-81·7)	12·8 (5·6-22·7)
Eritrea	396·4 (320·9-477·6)	174·7 (135·1-220·9)	132·1 (99·0-176·4)	122·6 (89·0-165·4)	48·8 (28·9-76·5)	12·2 (5·4-21·8)
Ethiopia	399·9 (324·3-477·7)	176·2 (135·6-221·4)	133·3 (100·3-176·8)	123·7 (89·1-165·3)	49·2 (29·6-76·7)	12·3 (5·5-21·9)
Kenya	405·2 (328·8-484·0)	178·5 (137·5-225·6)	135·0 (101·8-179·0)	125·3 (90·2-166·8)	49·8 (30·0-78·3)	12·4 (5·5-22·1)
Madagascar	397·6 (324·9-474·0)	175·2 (133·9-218·7)	132·5 (99·1-178·3)	123·0 (88·9-166·7)	48·9 (29·1-77·4)	12·2 (5·4-21·6)
Malawi	396·2 (319·8-477·4)	174·6 (133·1-221·7)	132·0 (98·3-173·8)	122·5 (89·0-163·5)	48·7 (29·1-76·5)	12·2 (5·3-21·7)
Mozambique	390·2 (314·2-467·2)	171·8 (132·2-215·2)	130·0 (97·5-173·2)	120·7 (86·1-162·2)	48·0 (28·0-75·2)	12·0 (5·3-21·3)
Rwanda	398·2 (321·5-476·0)	175·5 (135·1-221·7)	132·7 (98·7-176·3)	123·1 (86·6-164·2)	49·0 (28·8-76·9)	12·2 (5·4-21·7)
Somalia	388·1 (316·6-465·8)	171·0 (132·3-217·4)	129·3 (95·9-173·0)	120·0 (86·2-161·7)	47·7 (28·6-74·2)	11·9 (5·2-20·8)
South Sudan	398·4 (321·8-473·6)	175·5 (135·5-221·6)	132·8 (98·2-177·9)	123·2 (87·9-164·6)	49·0 (29·1-76·6)	12·3 (5·3-21·7)
Uganda	402·4 (328·0-481·5)	177·3 (136·4-225·2)	134·1 (99·5-179·3)	124·4 (88·6-166·4)	49·5 (29·5-77·3)	12·4 (5·4-22·1)
United Republic of Tanzania	401·2 (323·7-476·9)	176·8 (137·0-223·3)	133·7 (99·5-178·1)	124·1 (90·2-166·9)	49·4 (29·7-78·3)	12·3 (5·3-21·7)

Location	ASD without ID (95% UI)	ASD with borderline ID (95% UI)	ASD with mild ID (95% UI)	ASD with moderate ID (95% UI)	ASD with severe ID (95% UI)	ASD with profound ID (95% UI)
Zambia	400·6 (324·7-481·5)	176·5 (136·3-221·8)	133·5 (99·9-177·1)	123·8 (90·1-165·4)	49·3 (29·4-78·1)	12·3 (5·4-22·2)
**Southern Sub-Saharan Africa**	**404·0 (328·1-487·9)**	**178·0 (138·1-225·3)**	**134·6 (102·1-178·1)**	**124·9 (89·3-166·2)**	**49·7 (29·9-77·6)**	**12·4 (5·5-21·9)**
Botswana	405·0 (326·4-488·8)	178·4 (137·1-225·7)	135·0 (101·8-180·4)	125·3 (89·8-167·2)	49·8 (30·1-78·4)	12·5 (5·4-22·2)
Eswatini	396·4 (320·4-474·3)	174·6 (134·9-221·4)	132·1 (98·1-175·3)	122·6 (87·3-165·0)	48·7 (29·8-76·4)	12·2 (5·3-22·4)
Lesotho	389·4 (317·1-474·3)	171·5 (132·7-217·4)	129·8 (97·3-172·1)	120·5 (87·0-161·6)	47·9 (29·1-75·0)	12·0 (5·3-21·3)
Namibia	404·7 (326·3-491·3)	178·2 (138·1-225·3)	134·8 (100·5-179·8)	125·1 (89·7-168·3)	49·8 (30·0-78·1)	12·4 (5·4-22·3)
South Africa	407·6 (330·5-492·9)	179·6 (138·5-226·9)	135·8 (103·0-179·8)	126·0 (90·0-167·9)	50·1 (30·1-78·5)	12·5 (5·5-22·2)
Zimbabwe	390·7 (314·5-473·4)	172·1 (133·5-219·6)	130·2 (96·4-172·1)	120·8 (86·7-160·6)	48·1 (28·5-75·3)	12·0 (5·3-21·3)
**Western Sub-Saharan Africa**	**396·2 (323·5-473·5)**	**174·5 (134·4-220·3)**	**132·0 (99·7-175·0)**	**122·5 (87·6-163·1)**	**48·7 (29·3-75·9)**	**12·2 (5·4-21·5)**
Benin	397·5 (322·3-479·3)	175·1 (134·5-222·6)	132·4 (98·7-174·6)	122·9 (88·0-164·0)	48·9 (29·3-77·0)	12·2 (5·2-22·4)
Burkina Faso	391·5 (319·6-468·6)	172·5 (133·2-218·8)	130·5 (99·3-173·4)	121·1 (86·5-162·9)	48·1 (28·6-75·8)	12·0 (5·3-21·3)
Cabo Verde	425·4 (345·1-510·0)	187·4 (143·4-234·0)	141·8 (105·4-191·6)	131·6 (93·5-176·6)	52·3 (32·0-82·4)	13·1 (5·7-23·6)

Location	ASD without ID (95% UI)	ASD with borderline ID (95% UI)	ASD with mild ID (95% UI)	ASD with moderate ID (95% UI)	ASD with severe ID (95% UI)	ASD with profound ID (95% UI)
Cameroon	399·3 (323·5-480·2)	175·9 (134·7-223·7)	133·1 (98·8-175·5)	123·4 (89·0-163·3)	49·1 (29·6-78·2)	12·3 (5·3-21·8)
Chad	391·7 (317·9-471·5)	172·6 (132·9-221·2)	130·5 (96·0-175·1)	121·2 (85·4-163·2)	48·2 (28·7-75·3)	12·0 (5·3-21·2)
Côte d'Ivoire	406·5 (325·5-485·7)	179·1 (137·2-227·1)	135·5 (99·7-179·7)	125·7 (89·7-167·5)	50·0 (29·6-77·9)	12·5 (5·4-22·2)
Gambia	401·5 (323·6-479·9)	176·9 (136·3-224·0)	133·8 (99·1-179·3)	124·1 (90·0-166·6)	49·4 (29·5-76·2)	12·3 (5·3-21·8)
Ghana	400·0 (323·0-485·9)	176·1 (136·8-220·6)	133·3 (100·3-178·5)	123·7 (87·7-164·9)	49·2 (30·1-77·3)	12·3 (5·4-22·0)
Guinea	391·2 (314·4-472·8)	172·3 (133·0-220·6)	130·3 (97·0-172·0)	121·0 (86·2-161·1)	48·1 (28·5-74·8)	12·0 (5·2-21·7)
Guinea-Bissau	385·7 (310·8-462·2)	169·9 (131·0-214·7)	128·5 (96·4-169·3)	119·2 (85·2-159·3)	47·4 (28·2-75·0)	11·8 (5·1-21·1)
Liberia	404·7 (326·4-486·1)	178·3 (138·6-226·0)	134·9 (99·6-182·2)	125·1 (89·9-166·9)	49·8 (29·6-79·2)	12·4 (5·4-22·0)
Mali	395·6 (322·6-473·5)	174·3 (132·0-218·9)	131·8 (98·6-175·5)	122·3 (87·3-163·5)	48·7 (29·1-77·2)	12·2 (5·3-21·6)
Mauritania	408·8 (328·9-492·8)	180·1 (139·8-228·4)	136·2 (102·6-181·7)	126·4 (90·1-168·4)	50·3 (30·0-79·8)	12·5 (5·5-22·0)
Niger	392·1 (318·5-470·9)	172·8 (134·0-218·5)	130·7 (97·5-171·5)	121·3 (86·0-162·0)	48·2 (29·0-77·1)	12·0 (5·3-21·5)
Nigeria	394·3 (322·8-470·3)	173·7 (133·3-217·9)	131·4 (99·5-174·1)	121·9 (87·1-162·5)	48·5 (29·2-75·7)	12·1 (5·4-21·4)

Location	ASD without ID (95% UI)	ASD with borderline ID (95% UI)	ASD with mild ID (95% UI)	ASD with moderate ID (95% UI)	ASD with severe ID (95% UI)	ASD with profound ID (95% UI)
Sao Tome and Principe	412·9 (333·9-490·5)	181·9 (140·1-227·6)	137·6 (103·0-185·8)	127·7 (91·3-171·6)	50·8 (29·9-78·7)	12·7 (5·6-22·3)
Senegal	402·3 (326·4-486·9)	177·2 (135·8-224·5)	134·0 (100·3-178·1)	124·3 (89·1-165·3)	49·5 (30·0-77·1)	12·4 (5·4-22·3)
Sierra Leone	397·6 (320·0-479·6)	175·2 (135·5-220·2)	132·5 (99·1-175·8)	122·9 (87·8-164·1)	48·9 (29·3-76·9)	12·2 (5·4-21·8)
Togo	396·7 (318·4-473·6)	174·8 (135·2-222·7)	132·2 (99·6-175·8)	122·7 (88·4-164·4)	48·8 (29·5-76·7)	12·2 (5·4-21·7)

Note: ASD = Autism spectrum disorder. ID = Intellectual disability. UI = Uncertainty interval.

Table S7: Age-standardised DALY rates per 100 000 persons attributable to ASD by location and sex, 2021

Location	Both (95% UI)	Female (95% UI)	Male (95% UI)
**Global**	**147·6 (100·2-208·2)**	**94·5 (64·5-133·0)**	**199·8 (136·3-282·0)**
**Central Europe, Eastern Europe, and Central Asia**	**173·8 (118·9-244·0)**	**120·1 (81·6-166·7)**	**228·7 (157·2-321·8)**
**Central Asia**	**166·4 (113·2-235·6)**	**116·0 (80·1-165·1)**	**217·4 (147·4-307·6)**
Armenia	173·1 (118·2-240·4)	120·5 (83·3-169·9)	226·6 (154·3-318·4)
Azerbaijan	168·1 (117·3-234·1)	116·9 (80·6-163·1)	218·1 (152·1-304·9)
Georgia	170·9 (119·4-242·0)	119·1 (82·6-170·4)	222·6 (154·4-312·8)
Kazakhstan	168·5 (117·3-239·3)	117·6 (80·8-166·1)	222·0 (153·8-314·1)
Kyrgyzstan	167·2 (112·3-236·0)	116·7 (79·0-164·3)	219·4 (147·0-306·1)
Mongolia	161·2 (112·0-227·8)	113·3 (78·5-160·7)	210·8 (142·9-296·4)
Tajikistan	162·9 (111·2-230·1)	113·7 (77·6-162·6)	211·2 (144·6-297·2)
Turkmenistan	166·5 (111·7-233·7)	115·1 (78·8-160·7)	215·7 (145·5-301·2)
Uzbekistan	165·1 (112·3-232·7)	114·9 (79·7-159·5)	215·5 (146·1-303·4)
Central Europe	181·0 (125·2-253·0)	123·9 (84·1-171·0)	237·4 (165·2-334·6)
Albania	177·5 (121·7-249·6)	120·8 (83·1-172·9)	232·0 (160·4-325·4)
Bosnia and Herzegovina	182·3 (127·5-258·5)	125·2 (85·9-176·1)	238·7 (167·6-341·3)
Bulgaria	175·7 (121·8-249·1)	120·8 (82·9-171·0)	229·6 (160·0-320·8)
Croatia	187·1 (127·5-264·5)	126·5 (86·2-175·5)	247·0 (169·1-351·1)
Czechia	187·0 (131·1-261·6)	126·2 (86·5-176·1)	246·3 (170·0-344·8)
Hungary	182·5 (124·4-253·3)	124·5 (84·5-170·9)	240·8 (165·0-333·4)
Montenegro	183·5 (127·7-260·1)	124·3 (84·1-173·7)	241·5 (164·3-338·3)

Location	Both (95% UI)	Female (95% UI)	Male (95% UI)
North Macedonia	179·6 (123·1-251·7)	122·3 (83·4-170·5)	234·2 (162·7-327·7)
Poland	180·9 (125·2-251·8)	124·6 (84·6-172·0)	237·0 (165·6-332·0)
Romania	176·5 (121·7-244·6)	121·3 (82·8-167·6)	231·0 (158·8-320·7)
Serbia	182·1 (125·7-254·1)	123·8 (81·7-173·4)	237·4 (163·0-327·8)
Slovakia	181·4 (123·9-254·0)	124·1 (85·3-170·7)	238·2 (160·8-336·0)
Slovenia	190·9 (129·3-268·9)	128·1 (86·9-183·2)	250·9 (171·7-352·6)
**Eastern Europe**	**173·9 (119·2-243·4)**	**120·3 (81·7-166·2)**	**229·8 (158·3-321·8)**
Belarus	177·3 (121·2-247·7)	121·9 (83·1-169·1)	234·8 (160·2-331·4)
Estonia	181·0 (124·1-249·0)	122·9 (85·4-172·2)	239·2 (163·4-326·7)
Latvia	176·9 (122·6-248·0)	121·4 (83·3-171·1)	233·7 (162·3-328·1)
Lithuania	177·3 (121·5-247·7)	121·5 (83·6-170·2)	234·8 (158·5-329·2)
Republic of Moldova	172·5 (116·8-244·3)	119·0 (81·8-164·4)	227·1 (152·0-322·9)
Russian Federation	173·9 (119·7-244·0)	120·3 (81·5-166·6)	230·1 (159·7-322·7)
Ukraine	172·7 (119·1-240·3)	119·9 (82·4-167·2)	227·4 (156·8-315·7)
**High-income**	**204·1 (140·7-284·7)**	**120·8 (81·9-170·1)**	**286·4 (198·8-402·3)**
**Australasia**	**222·6 (154·0-314·3)**	**131·8 (87·9-186·0)**	**312·8 (219·1-437·2)**
Australia	223·5 (155·0-315·0)	132·2 (87·9-187·0)	314·4 (217·7-440·1)
New Zealand	218·3 (150·3-306·7)	129·8 (88·4-180·6)	304·7 (209·3-423·9)
**High-income Asia Pacific**	**293·9 (203·2-413·0)**	**176·0 (120·8-244·4)**	**407·9 (282·8-578·4)**
Brunei Darussalam	257·7 (176·3-361·1)	156·3 (109·0-219·5)	349·3 (238·0-485·3)
Japan	299·1 (207·2-420·0)	177·1 (120·8-246·0)	418·1 (291·3-586·4)
Republic of Korea	283·8 (196·0-395·6)	175·0 (121·1-246·3)	387·6 (267·6-540·9)

Location	Both (95% UI)	Female (95% UI)	Male (95% UI)
Singapore	280·9 (193·9-388·0)	167·7 (114·9-230·5)	390·8 (268·0-542·7)
**High-income North America**	**204·0 (141·1-285·5)**	**130·6 (89·2-182·8)**	**277·1 (192·5-388·2)**
Canada	209·6 (146·9-296·0)	133·6 (93·1-188·1)	284·8 (195·4-409·1)
Greenland	190·4 (131·3-267·9)	122·7 (84·6-168·3)	252·5 (172·0-352·6)
United States of America	203·3 (141·0-283·7)	130·3 (88·7-182·3)	276·2 (192·2-386·3)
**Southern Latin America**	**198·2 (134·7-280·3)**	**123·4 (83·8-172·8)**	**274·3 (185·6-383·1)**
Argentina	196·3 (133·8-274·6)	122·6 (84·2-169·9)	271·6 (184·4-377·3)
Chile	202·8 (139·6-289·4)	125·0 (84·5-178·7)	280·7 (194·9-393·3)
Uruguay	198·6 (137·5-283·6)	123·9 (84·7-177·3)	275·6 (189·9-392·7)
**Western Europe**	**168·3 (115·5-234·8)**	**88·8 (61·4-124·2)**	**246·3 (170·4-341·8)**
Andorra	170·1 (116·1-239·3)	90·3 (61·3-127·2)	245·3 (168·6-343·4)
Austria	167·8 (113·2-238·4)	90·6 (61·4-128·0)	242·6 (163·7-342·8)
Belgium	166·2 (113·2-232·5)	90·1 (61·4-126·3)	241·2 (164·0-337·8)
Cyprus	166·0 (112·3-233·2)	90·1 (61·2-122·7)	241·5 (165·2-342·0)
Denmark	177·7 (119·7-245·0)	95·8 (65·3-131·4)	257·2 (176·4-357·2)
Finland	155·5 (106·7-218·3)	88·2 (60·3-124·3)	220·6 (151·1-312·1)
France	165·2 (112·7-232·3)	89·5 (61·2-127·0)	241·2 (166·4-336·1)
Germany	168·4 (116·2-233·5)	90·0 (61·9-126·2)	242·6 (167·3-338·1)
Greece	165·3 (111·5-233·2)	90·2 (62·2-127·0)	240·5 (160·2-341·7)
Iceland	196·9 (136·2-273·2)	105·4 (71·2-145·8)	284·4 (197·4-393·9)
Ireland	200·9 (138·5-279·3)	111·0 (76·2-158·0)	290·9 (199·9-403·1)
Israel	149·9 (102·6-211·2)	83·7 (57·9-119·8)	215·3 (146·4-300·2)

Location	Both (95% UI)	Female (95% UI)	Male (95% UI)
Italy	187·7 (129·6-263·8)	104·1 (71·3-146·2)	269·5 (186·7-381·1)
Luxembourg	169·4 (116·7-234·6)	90·6 (61·2-127·9)	245·1 (170·0-340·3)
Malta	168·9 (113·8-236·8)	90·4 (62·3-127·8)	243·8 (163·0-340·9)
Monaco	167·3 (115·2-235·1)	89·7 (62·2-123·6)	245·8 (168·7-345·6)
Netherlands	169·0 (117·4-236·9)	90·6 (61·7-127·2)	246·0 (170·6-345·5)
Norway	151·9 (104·7-214·4)	82·8 (56·7-116·6)	217·8 (149·9-304·9)
Portugal	161·5 (109·7-227·9)	88·4 (60·6-123·4)	236·2 (161·3-332·5)
San Marino	167·5 (114·9-234·0)	90·4 (62·0-128·1)	245·8 (168·4-343·7)
Spain	192·8 (134·5-265·4)	84·1 (58·0-117·5)	300·1 (207·4-415·3)
Sweden	191·3 (130·9-270·0)	93·7 (62·6-133·2)	284·4 (196·4-403·5)
Switzerland	169·3 (117·5-235·9)	90·2 (61·9-126·0)	245·6 (171·1-342·9)
United Kingdom	140·6 (95·4-198·1)	75·0 (52·0-105·0)	206·6 (140·6-290·1)
**Latin America and Caribbean**	**129·0 (87·1-181·3)**	**86·4 (58·7-121·0)**	**172·6 (116·8-243·8)**
**Andean Latin America**	**128·5 (86·8-180·9)**	**86·6 (59·7-120·6)**	**170·0 (116·3-238·0)**
Bolivia (Plurinational State of)	123·9 (86·2-174·0)	84·1 (58·0-118·6)	163·7 (113·7-228·6)
Ecuador	128·2 (86·2-178·4)	87·0 (59·8-122·6)	169·7 (114·9-234·0)
Peru	130·1 (88·4-182·7)	87·2 (59·9-121·4)	172·2 (119·7-241·8)
**Caribbean**	**127·5 (86·5-178·1)**	**86·2 (60·1-120·9)**	**169·2 (115·7-235·8)**
Antigua and Barbuda	133·2 (91·2-187·1)	89·7 (61·8-127·1)	177·6 (122·4-247·9)
Bahamas	129·8 (88·2-180·9)	88·6 (61·7-124·0)	173·1 (117·3-242·2)
Barbados	133·6 (90·2-188·7)	90·0 (60·4-123·5)	178·3 (118·9-253·1)
Belize	127·8 (86·1-179·0)	87·0 (59·2-124·3)	169·2 (114·3-236·4)

Location	Both (95% UI)	Female (95% UI)	Male (95% UI)
Bermuda	139·2 (94·6-196·9)	92·1 (62·2-129·7)	187·0 (127·1-265·4)
Cuba	135·7 (92·9-190·6)	90·4 (62·7-127·4)	179·7 (123·7-252·6)
Dominica	130·2 (90·1-185·4)	88·0 (60·2-124·9)	170·6 (120·0-240·8)
Dominican Republic	126·9 (86·8-176·6)	85·9 (59·2-119·6)	167·3 (114·4-231·4)
Grenada	130·5 (90·1-182·8)	87·7 (60·1-125·3)	170·9 (117·6-237·2)
Guyana	123·2 (83·4-171·4)	84·4 (58·6-118·4)	162·9 (109·5-224·9)
Haiti	116·5 (79·5-164·7)	79·5 (55·0-112·9)	155·2 (105·5-220·9)
Jamaica	131·8 (92·2-182·6)	88·8 (61·2-124·7)	174·9 (122·9-242·0)
Puerto Rico	136·9 (93·4-190·8)	92·2 (62·2-128·9)	183·3 (126·6-252·3)
Saint Kitts and Nevis	130·1 (89·4-182·3)	87·9 (59·9-124·3)	172·0 (117·8-242·0)
Saint Lucia	131·2 (88·7-184·5)	88·6 (60·5-124·3)	173·0 (116·1-245·0)
Saint Vincent and the Grenadines	129·7 (88·6-182·4)	87·4 (60·8-123·8)	170·2 (116·0-239·3)
Suriname	125·5 (85·8-175·1)	85·1 (58·5-118·2)	166·4 (113·8-232·9)
Trinidad and Tobago	130·7 (89·3-181·4)	88·2 (60·2-125·2)	172·4 (115·9-239·6)
United States Virgin Islands	132·9 (91·1-185·5)	89·6 (61·6-123·8)	176·6 (121·9-249·3)
**Central Latin America**	**142·2 (96·2-201·3)**	**95·1 (64·8-133·3)**	**191·1 (129·1-270·8)**
Colombia	145·0 (98·1-204·3)	96·7 (65·7-137·0)	194·3 (131·8-273·1)
Costa Rica	148·6 (101·5-208·9)	99·3 (68·0-140·1)	200·3 (135·9-278·5)
El Salvador	139·5 (94·5-194·8)	95·0 (65·5-134·1)	189·4 (129·7-267·0)
Guatemala	135·4 (93·7-191·1)	91·8 (62·5-132·3)	181·8 (126·7-255·0)
Honduras	136·2 (92·7-192·6)	92·0 (62·7-131·7)	183·3 (126·7-257·0)
Mexico	142·2 (95·9-199·9)	94·9 (64·9-133·9)	191·2 (129·2-268·9)

Location	Both (95% UI)	Female (95% UI)	Male (95% UI)
Nicaragua	141·5 (95·9-197·8)	94·9 (65·5-132·9)	189·3 (126·2-265·5)
Panama	146·3 (101·1-207·4)	96·4 (66·7-132·5)	195·1 (135·5-278·0)
Venezuela (Bolivarian Republic of)	142·0 (98·5-199·8)	95·5 (66·6-134·8)	191·2 (131·0-271·7)
**Tropical Latin America**	**114·4 (77·1-160·3)**	**76·3 (51·7-108·4)**	**153·4 (103·6-215·3)**
Brazil	114·3 (77·0-160·1)	76·2 (51·8-108·5)	153·4 (103·5-215·3)
Paraguay	117·4 (79·0-161·8)	78·1 (53·5-111·4)	155·8 (105·7-215·7)
**North Africa and Middle East**	**144·2 (97·2-202·5)**	**97·1 (66·5-136·8)**	**187·9 (127·0-265·0)**
Afghanistan	130·4 (89·0-183·2)	89·8 (62·2-127·4)	170·7 (116·1-240·7)
Algeria	145·2 (97·3-202·0)	98·5 (66·6-137·4)	190·5 (127·6-266·0)
Bahrain	158·7 (110·5-221·9)	100·7 (70·0-143·6)	197·0 (138·0-275·1)
Egypt	143·0 (96·7-202·4)	96·9 (66·0-137·1)	185·9 (124·9-261·6)
Iran (Islamic Republic of)	147·8 (100·6-208·8)	97·6 (66·3-136·5)	196·2 (134·2-276·5)
Iraq	132·0 (90·6-184·9)	91·6 (63·9-127·3)	169·8 (116·2-240·7)
Jordan	151·7 (103·3-212·3)	100·1 (67·6-139·9)	196·7 (133·5-276·8)
Kuwait	155·0 (104·3-218·7)	102·9 (67·9-142·4)	201·6 (135·2-280·6)
Lebanon	162·5 (108·4-226·9)	112·7 (76·1-155·1)	211·6 (143·6-296·6)
Libya	143·6 (97·2-202·8)	97·3 (65·6-136·1)	187·5 (126·2-264·6)
Morocco	141·2 (97·0-197·0)	96·2 (64·9-133·9)	185·4 (128·6-260·4)
Oman	156·1 (104·9-218·7)	100·7 (67·8-142·0)	194·8 (131·6-273·1)
Palestine	145·9 (99·9-204·5)	99·8 (67·5-138·8)	190·5 (129·3-267·6)
Qatar	180·6 (123·6-251·2)	108·2 (73·3-149·3)	220·4 (150·9-305·8)
Saudi Arabia	152·8 (104·9-218·5)	99·3 (68·5-140·6)	192·5 (130·7-275·1)

Location	Both (95% UI)	Female (95% UI)	Male (95% UI)
Sudan	137·5 (94·4-193·5)	93·7 (64·7-132·5)	179·9 (123·6-251·3)
Syrian Arab Republic	141·1 (96·7-200·1)	98·3 (66·4-138·2)	189·1 (131·4-268·1)
Tunisia	146·6 (99·6-206·8)	100·0 (68·1-137·9)	193·0 (131·6-272·0)
Türkiye	147·6 (99·7-207·0)	100·1 (68·4-141·3)	194·3 (131·8-273·0)
United Arab Emirates	157·2 (107·8-220·9)	98·1 (68·3-137·3)	190·5 (130·6-268·9)
Yemen	134·8 (93·4-191·5)	92·4 (63·5-130·6)	176·9 (122·6-249·9)
**South Asia**	**127·9 (87·3-180·3)**	**86·2 (59·6-121·2)**	**167·9 (114·2-235·6)**
Bangladesh	110·3 (75·4-154·6)	72·2 (49·5-102·2)	149·6 (102·8-210·0)
Bhutan	121·3 (84·9-169·6)	80·9 (54·7-116·6)	159·1 (111·2-221·2)
India	131·9 (89·8-185·2)	89·2 (61·7-125·4)	172·4 (117·1-242·3)
Nepal	116·9 (78·7-164·4)	79·9 (55·4-113·3)	157·6 (105·9-221·8)
Pakistan	117·5 (81·3-164·4)	79·0 (53·9-111·5)	154·2 (106·8-215·6)
**Southeast Asia, East Asia, and Oceania**	**126·5 (86·0-178·0)**	**70·3 (47·3-99·5)**	**179·8 (123·0-254·0)**
**East Asia**	**125·1 (84·5-176·3)**	**61·2 (40·6-86·8)**	**184·2 (125·4-259·8)**
China	124·2 (83·7-175·1)	60·0 (39·9-85·3)	183·4 (124·7-258·8)
Democratic People's Republic of Korea	133·9 (93·5-187·1)	80·6 (54·8-112·8)	186·3 (129·8-259·9)
Taiwan (Province of China)	172·2 (119·4-241·7)	110·6 (74·0-154·9)	232·5 (160·9-325·0)
**Oceania**	**126·1 (85·7-176·8)**	**80·8 (55·1-112·3)**	**168·5 (116·7-234·7)**
American Samoa	132·7 (91·1-189·4)	84·7 (57·4-121·1)	178·6 (122·1-251·0)
Cook Islands	135·9 (92·4-189·7)	86·6 (58·5-122·1)	188·0 (127·7-261·9)
Fiji	128·4 (88·2-184·2)	83·1 (56·1-116·9)	172·5 (117·3-247·9)
Guam	136·8 (92·2-192·8)	86·3 (59·5-122·3)	185·0 (124·0-260·3)

Location	Both (95% UI)	Female (95% UI)	Male (95% UI)
Kiribati	121·3 (83·7-170·1)	79·8 (53·8-114·4)	165·2 (114·1-229·0)
Marshall Islands	126·6 (88·6-179·3)	81·6 (57·4-113·5)	169·7 (118·1-242·1)
Micronesia (Federated States of)	128·3 (87·0-180·8)	82·7 (56·3-115·5)	172·4 (115·2-243·7)
Nauru	126·1 (86·0-177·8)	81·7 (56·2-114·2)	170·5 (115·3-241·6)
Niue	131·6 (89·9-184·0)	85·0 (56·8-120·5)	176·8 (120·8-246·4)
Northern Mariana Islands	138·1 (94·9-193·0)	86·5 (58·1-120·9)	184·7 (127·4-256·0)
Palau	136·1 (92·0-191·8)	84·0 (57·9-118·6)	178·3 (120·0-249·0)
Papua New Guinea	125·4 (86·1-174·8)	80·2 (54·3-111·7)	167·3 (115·8-233·0)
Samoa	130·9 (87·8-184·5)	83·7 (56·2-117·2)	175·6 (121·7-247·4)
Solomon Islands	126·1 (86·2-176·8)	81·3 (56·1-114·5)	169·4 (117·0-235·9)
Tokelau	134·1 (92·2-189·3)	84·8 (56·4-118·8)	181·1 (125·2-255·5)
Tonga	130·7 (89·5-185·5)	84·3 (57·4-120·1)	178·0 (122·3-250·9)
Tuvalu	129·0 (87·1-180·9)	82·0 (56·1-114·8)	171·9 (116·5-242·9)
Vanuatu	126·0 (85·9-179·0)	82·1 (55·0-118·9)	169·3 (115·0-239·8)
**Southeast Asia**	**128·6 (87·5-181·7)**	**85·9 (58·5-120·9)**	**171·0 (116·5-241·5)**
Cambodia	126·4 (85·8-179·8)	82·5 (55·9-115·2)	172·1 (116·7-241·2)
Indonesia	122·0 (81·9-172·0)	89·7 (60·8-125·8)	153·4 (103·0-216·9)
Lao People's Democratic Republic	126·1 (85·9-177·6)	81·9 (55·7-116·7)	169·9 (115·9-242·0)
Malaysia	138·2 (95·5-194·9)	87·5 (60·0-124·1)	185·5 (127·2-254·4)
Maldives	144·4 (97·2-203·4)	85·3 (58·2-122·2)	186·1 (124·8-261·9)
Mauritius	136·3 (92·5-190·6)	86·9 (59·0-121·9)	185·8 (125·5-260·0)
Myanmar	125·8 (85·4-178·0)	82·2 (55·8-115·2)	172·2 (116·8-242·0)

Location	Both (95% UI)	Female (95% UI)	Male (95% UI)
Philippines	129·0 (87·3-181·6)	83·3 (57·1-117·8)	173·4 (118·0-245·7)
Seychelles	138·2 (94·3-194·6)	87·3 (58·4-122·5)	183·8 (125·1-257·8)
Sri Lanka	140·8 (97·4-197·9)	87·1 (58·6-123·6)	197·1 (136·0-276·4)
Thailand	136·0 (93·0-192·7)	86·9 (57·8-121·3)	186·6 (130·0-261·4)
Timor-Leste	126·9 (89·2-179·6)	82·4 (57·0-117·1)	170·5 (118·3-241·1)
Viet Nam	137·3 (95·2-193·1)	79·3 (53·2-110·7)	194·8 (134·8-272·7)
**Sub-Saharan Africa**	**166·0 (113·5-233·6)**	**116·3 (80·8-164·1)**	**218·1 (148·1-306·9)**
**Central Sub-Saharan Africa**	**164·7 (112·4-231·9)**	**115·5 (79·7-164·1)**	**214·8 (145·2-302·6)**
Angola	163·8 (114·2-227·4)	116·1 (79·8-163·0)	215·7 (148·2-300·9)
Central African Republic	157·4 (106·8-223·7)	111·8 (76·1-159·3)	205·7 (140·1-293·3)
Congo	166·8 (115·3-234·3)	116·4 (78·4-162·1)	217·9 (153·5-307·5)
Democratic Republic of the Congo	165·1 (112·3-232·4)	115·4 (78·2-163·4)	214·5 (146·7-306·0)
Equatorial Guinea	172·9 (118·2-247·2)	117·7 (80·2-166·4)	223·2 (152·6-318·3)
Gabon	168·5 (114·2-234·2)	118·4 (80·5-166·3)	222·4 (149·6-310·0)
**Eastern Sub-Saharan Africa**	**166·8 (114·1-234·9)**	**116·6 (81·0-164·2)**	**218·4 (148·7-307·6)**
Burundi	165·7 (114·8-232·7)	115·3 (80·3-162·1)	215·0 (149·2-304·9)
Comoros	170·1 (118·7-238·1)	118·6 (82·1-166·9)	222·0 (154·7-310·0)
Djibouti	174·7 (123·3-247·3)	118·8 (82·6-168·4)	222·4 (154·6-315·9)
Eritrea	165·7 (114·8-233·7)	116·5 (80·3-164·4)	214·8 (148·3-301·6)
Ethiopia	167·1 (114·8-234·2)	116·4 (79·9-161·9)	217·3 (149·4-307·4)
Kenya	169·7 (116·2-238·2)	118·6 (82·3-166·7)	221·9 (151·7-312·2)
Madagascar	166·5 (116·2-231·6)	116·5 (80·9-161·7)	218·2 (151·1-308·0)

Location	Both (95% UI)	Female (95% UI)	Male (95% UI)
Malawi	165·6 (112·8-232·8)	116·0 (79·9-166·6)	218·6 (150·7-307·3)
Mozambique	161·8 (111·9-225·7)	113·8 (77·5-161·1)	214·8 (151·5-301·8)
Rwanda	166·5 (113·2-232·4)	117·0 (80·3-166·2)	220·0 (149·5-302·2)
Somalia	162·2 (111·3-227·5)	113·7 (77·8-159·0)	210·8 (146·5-298·7)
South Sudan	165·5 (115·3-231·2)	115·5 (80·2-161·9)	216·2 (148·3-303·3)
Uganda	167·9 (118·2-234·4)	117·9 (81·9-166·5)	221·3 (156·6-310·2)
United Republic of Tanzania	167·6 (115·1-236·8)	117·4 (79·9-167·5)	221·2 (150·1-308·9)
Zambia	166·4 (114·6-234·3)	116·5 (81·5-164·3)	217·4 (148·6-304·8)
**Southern Sub-Saharan Africa**	**167·7 (114·9-236·1)**	**117·3 (80·6-163·5)**	**220·8 (151·5-311·8)**
Botswana	168·4 (118·5-236·3)	117·8 (83·2-164·6)	220·5 (153·1-311·0)
Eswatini	164·4 (114·2-229·1)	116·0 (81·6-162·6)	216·1 (148·7-302·1)
Lesotho	161·2 (111·2-222·2)	114·2 (76·4-163·0)	212·1 (142·3-291·0)
Namibia	168·8 (115·9-237·5)	119·2 (82·0-168·8)	222·2 (151·9-313·8)
South Africa	169·1 (115·8-237·7)	117·6 (82·0-164·7)	222·4 (151·6-313·9)
Zimbabwe	163·0 (111·9-229·6)	115·6 (78·7-162·2)	215·5 (149·4-302·4)
**Western Sub-Saharan Africa**	**165·5 (113·5-232·4)**	**116·0 (80·7-163·1)**	**218·3 (148·4-306·6)**
Benin	166·2 (115·6-231·0)	116·0 (79·7-164·2)	218·9 (150·9-306·1)
Burkina Faso	163·7 (114·9-228·4)	115·1 (78·6-160·0)	216·3 (151·7-302·2)
Cabo Verde	178·5 (122·8-251·3)	123·3 (84·2-169·3)	232·8 (161·3-330·1)
Cameroon	167·0 (114·3-232·9)	116·6 (78·7-166·0)	218·0 (150·1-302·2)
Chad	163·3 (112·9-229·6)	114·1 (77·8-161·0)	213·1 (143·4-298·5)
Côte d'Ivoire	169·9 (118·7-236·8)	116·5 (79·5-163·6)	219·5 (150·4-304·5)

Location	Both (95% UI)	Female (95% UI)	Male (95% UI)
Gambia	167·6 (116·8-232·6)	116·6 (81·0-165·7)	220·4 (153·3-305·2)
Ghana	167·6 (112·0-234·3)	117·8 (80·6-164·5)	221·4 (147·3-312·1)
Guinea	163·2 (111·0-228·8)	114·7 (78·8-160·0)	215·4 (149·7-304·3)
Guinea-Bissau	161·4 (112·1-226·0)	114·1 (80·9-162·2)	212·4 (146·7-296·8)
Liberia	167·4 (114·2-232·3)	114·9 (76·6-160·3)	218·2 (150·9-302·6)
Mali	164·8 (113·2-232·4)	114·8 (80·6-163·1)	215·8 (146·7-303·3)
Mauritania	171·6 (117·0-243·7)	119·4 (81·3-167·3)	226·0 (154·6-319·6)
Niger	164·2 (112·5-227·7)	114·7 (79·5-160·8)	215·5 (146·9-298·7)
Nigeria	164·6 (112·9-231·7)	116·1 (80·6-163·2)	218·6 (148·6-308·5)
Sao Tome and Principe	173·6 (116·0-247·1)	120·6 (84·1-170·1)	226·2 (150·7-324·8)
Senegal	168·1 (117·5-235·9)	116·9 (80·6-165·4)	219·7 (150·6-304·7)
Sierra Leone	166·0 (115·1-235·1)	115·6 (76·5-165·4)	216·8 (149·1-308·6)
Togo	165·8 (115·4-230·2)	116·0 (81·1-162·9)	219·1 (149·4-303·9)

Note: UI = Uncertainty interval.

Section 3. References

References

1. Zheng P, Barber R, Sorensen RJD, Murray CJL, Aravkin AY. Trimmed Constrained Mixed Effects Models: Formulations and Algorithms. *Journal of Computational and Graphical Statistics* 2021; **30**(3): 544-56.
2. Fullman N, Yearwood J, Abay SM, et al. Measuring performance on the Healthcare Access and Quality Index for 195 countries and territories and selected subnational locations: a systematic analysis from the Global Burden of Disease Study 2016. *The Lancet* 2018; **391**(10136): 2236-71.
3. Viechtbauer W. Conducting meta-analyses in R with the metafor package. *Journal of Statistical Software* 2010; **36**(3): 1-48.
4. R Core Team. R: A Language and Environment for Statistical Computing. Vienna, Austria: R Foundation for Statistical Computing; 2017.
5. Chakrabarti S, Fombonne E. Pervasive developmental disorders in preschool children. *JAMA: Journal of the American Medical Association* 2001; **285**(24): 3093-9.
6. Chakrabarti S, Fombonne E. Pervasive Developmental Disorders in Preschool Children: Confirmation of High Prevalence. *The American Journal of Psychiatry* 2005; **162**(6): 1133-41.
7. Kadesjo B, Gillberg C, Hagberg B. Brief report: autism and Asperger syndrome in seven-year-old children: a total population study. *J Autism Dev Disord* 1999; **29**(4): 327-31.
8. Ellefsen A, Kampmann H, Billstedt E, Gillberg IC, Gillberg C. Autism in the Faroe Islands. An epidemiological study. *Journal of Autism and Developmental Disorders* 2007; **37**(3): 437-44.
9. Kocovska E, Biskupsto R, Carina Gillberg I, et al. The rising prevalence of autism: a prospective longitudinal study in the Faroe Islands. *J Autism Dev Disord* 2012; **42**(9): 1959-66.
10. Mattila M-L, Kielinen M, Linna S-L, et al. Autism spectrum disorders according to DSM-IV-TR and comparison with DSM-5 draft criteria: An epidemiological study. *Journal of the American Academy of Child & Adolescent Psychiatry* 2011; **50**(6): 583-92.
11. Moher D, Liberati A, Tetzlaff J, Altman DG. Preferred reporting items for systematic reviews and meta-analyses: the PRISMA statement. *Ann Intern Med* 2009; **151**(4): 264-9, w64.
12. Page MJ, McKenzie JE, Bossuyt PM, et al. The PRISMA 2020 statement: an updated guideline for reporting systematic reviews. *Bmj* 2021; **372**: n71.
13. Kocovska E, Billstedt E, Ellefsen A, et al. Autism in the Faroe Islands: diagnostic stability from childhood to early adult life. *ScientificWorldJournal* 2013; **2013**: 592371.
14. Honda H, Shimizu Y, Rutter M. No effect of MMR withdrawal on the incidence of autism: a total population study. *Journal of Child Psychology and Psychiatry* 2005; **46**(6): 572-9.
15. Baird G, Charman T, Baron-Cohen S, et al. A screening instrument for autism at 18 months of age: a 6-year follow-up study. *J Am Acad Child Adolesc Psychiatry* 2000; **39**(6): 694-702.
16. Fombonne E, Marcin C, Manero AC, et al. Prevalence of Autism Spectrum Disorders in Guanajuato, Mexico: The Leon survey. *Journal of Autism and Developmental Disorders* 2016; **46**(5): 1669-85.
17. Posserud M, Lundervold AJ, Lie SA, Gillberg C. The prevalence of autism spectrum disorders: Impact of diagnostic instrument and non-response bias. *Social Psychiatry and Psychiatric Epidemiology* 2010; **45**(3): 319-27.
18. Kawamura Y, Takahashi O, Ishii T. Reevaluating the incidence of pervasive developmental disorders: Impact of elevated rates of detection through implementation of an integrated system of screening in Toyota, Japan. *Psychiatry and Clinical Neurosciences* 2008; **62**(2): 152-9.
19. Saemundsen E, Magnússon P, Georgsdóttir I, Egilsson E, Rafnsson V. Prevalence of autism spectrum disorders in an Icelandic birth cohort. *BMJ Open* 2013; **3**(6).
20. Idring S, Lundberg M, Sturm H, et al. Changes in Prevalence of Autism Spectrum Disorders in 2001–2011: Findings from the Stockholm Youth Cohort. *Journal of Autism and Developmental Disorders* 2015; **45**(6): 1766-73.

21. Kantzer AK, Fernell E, Westerlund J, Hagberg B, Gillberg C, Miniscalco C. Young children who screen positive for autism: Stability, change and comorbidity over two years. *Research in Developmental Disabilities* 2016.
22. Bertrand J, Mars A, Boyle C, Bove F, Yeargin-Allsopp M, Decoufle P. Prevalence of autism in a United States population: the Brick Township, New Jersey, investigation. *Pediatrics* 2001; **108**(5): 1155-61.
23. Saito M, Hirota T, Sakamoto Y, et al. Prevalence and cumulative incidence of autism spectrum disorders and the patterns of co-occurring neurodevelopmental disorders in a total population sample of 5-year-old children. *Mol Autism* 2020; **11**(1): 35.
24. Zhou H, Xu X, Yan W, et al. Prevalence of Autism Spectrum Disorder in China: A Nationwide Multi-center Population-based Study Among Children Aged 6 to 12 Years. *Neurosci Bull* 2020; **36**(9): 961-71.
25. Kamio Y, Inada N, Koyama T, Inokuchi E, Tsuchiya K, Kuroda M. Effectiveness of using the Modified Checklist for Autism in Toddlers in two-stage screening of autism spectrum disorder at the 18-month health check-up in Japan. *J Autism Dev Disord* 2014; **44**(1): 194-203.
26. Bryson SE, Clark BS, Smith IM. First report of a Canadian epidemiological study of autistic syndromes. *Child Psychology & Psychiatry & Allied Disciplines* 1988; **29**(4): 433-45.
27. Croen LA, Grether JK, Hoogstrate J, Selvin S. The changing prevalence of autism in California. *Journal of Autism and Developmental Disorders* 2002; **32**(3): 207-15.
28. Yeargin-Allsopp M, Rice C, Karapurkar T, Doernberg N, Boyle C, Murphy C. Prevalence of autism in a US metropolitan area. *JAMA: Journal of the American Medical Association* 2003; **289**(1): 49-55.
29. Barbaresi WJ, Katusic SK, Colligan RC, Weaver AL, Jacobsen SJ. The incidence of autism in Olmsted County, Minnesota, 1976-1997: results from a population-based study. *Arch Pediatr Adolesc Med* 2005; **159**(1): 37-44.
30. Ritvo ER, Freeman BJ, Pingree C, et al. The UCLA-University of Utah epidemiologic survey of autism: Prevalence. *The American Journal of Psychiatry* 1989; **146**(2): 194-9.
31. Wei Z, Ren L, Weng L, et al. Early screening on autism spectrum disorders in 18-24 months old children in Shenzhen: 3 years follow up study. *Chin J Child Health Care* 2012; **20**(4): 354–7.
32. Chen Y, Huang H, Zhao Y, et al. Epidemiological survey on autism spectrum disorders in 2 to 6 years old children in Ranghulu district in Daqing city. *China J Child Health Care* 2010; **18**(4): 331-3.
33. Nygren G, Cederlund M, Sandberg E, et al. The prevalence of autism spectrum disorders in toddlers: A population study of 2-year-old Swedish children. *Journal of Autism and Developmental Disorders* 2012; **42**(7): 1491-7.
34. Samadi SA, McConkey R. Screening for autism in Iranian preschoolers: Contrasting M-CHAT and a scale developed in Iran. *Journal of Autism and Developmental Disorders* 2015; **45**(9): 2908-16.
35. Wang X, Yang W-H, Jin Y, et al. Prevalence of autism spectrum disorders in preschool children of Guangzhou kindergartens. *Chinese Mental Health Journal* 2011; **25**(6): 401-8.
36. Surén P, Schjølberg S, Øyen AS, et al. The autism birth cohort (ABC): A study of autism spectrum disorders in MoBa. *Norsk Epidemiologi* 2014; **24**(1-2): 39-50.
37. Elberling H, Linneberg A, Rask CU, Houman T, Goodman R, Mette Skovgaard A. Psychiatric disorders in Danish children aged 5-7 years: A general population study of prevalence and risk factors from the Copenhagen Child Cohort (CCC 2000). *Nord J Psychiatry* 2016; **70**(2): 146-55.
38. Kim YS, Leventhal BL, Koh Y-J, et al. Prevalence of autism spectrum disorders in a total population sample. *The American Journal of Psychiatry* 2011; **168**(9): 904-12.
